# Targeting cardiac fibrosis in heart failure with preserved ejection fraction: mirage or miracle?

**DOI:** 10.15252/emmm.201910865

**Published:** 2020-09-21

**Authors:** Mark Sweeney, Ben Corden, Stuart A Cook

**Affiliations:** ^1^ MRC‐London Institute of Medical Sciences Hammersmith Hospital Campus London UK; ^2^ Wellcome Trust 4i/NIHR Clinical Research Fellow Imperial College London UK; ^3^ National Heart Research Institute Singapore National Heart Centre Singapore Singapore Singapore; ^4^ Cardiovascular and Metabolic Disorders Program Duke‐National University of Singapore Medical School Singapore Singapore; ^5^ National Heart and Lung Institute Imperial College London London UK

**Keywords:** CMR, fibroblast, fibrosis, heart failure, HFpEF, Cardiovascular System

## Abstract

Cardiac fibrosis is central to the pathology of heart failure, particularly heart failure with preserved ejection fraction (HFpEF). Irrespective of the underlying profibrotic condition (e.g. ageing, diabetes, hypertension), maladaptive cardiac fibrosis is defined by the transformation of resident fibroblasts to matrix‐secreting myofibroblasts. Numerous profibrotic factors have been identified at the molecular level (e.g. TGFβ, IL11, AngII), which activate gene expression programs for myofibroblast activation. A number of existing HF therapies indirectly target fibrotic pathways; however, despite multiple clinical trials in HFpEF, a specific clinically effective antifibrotic therapy remains elusive. Therapeutic inhibition of TGFβ, the master‐regulator of fibrosis, has unfortunately proven toxic and ineffective in clinical trials to date, and new approaches are needed. In this review, we discuss the pathophysiology and clinical implications of interstitial fibrosis in HFpEF. We provide an overview of trials targeting fibrosis in HFpEF to date and discuss the promise of potential new therapeutic approaches and targets in the context of underlying molecular mechanisms.

## Graphical abstract

This review discusses recent advances in novel therapeutic approaches against cardiac fibrosis in heart failure with preserved ejection fraction and their underlying molecular mechanisms.

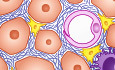

**Glossory**
Cardiac fibroblast: Cells resident within the myocardium which express mesenchymal markers and secrete ECM proteins.
Diabetic nephropathy: Kidney disease caused by the effects of hyperglycaemia on the structure and function of the glomerulus.
Dilated cardiomyopathy: Heart muscle disease, often with a genetic component, characterized by progressive dilation and dysfunction of the cardiac chambers leading to heart failure.
Extracellular matrix: A complex network of interconnected structural proteins, glycoproteins and enzymes which reside in the extracellular space and provide structural and biochemical support to surrounding cells.
Extracellular volume: Magnetic resonance imaging measurement technique which allows the quantification of diffuse collagen deposition within a tissue.
Fibrosis: The accumulation of excess extracellular matrix proteins within the extracellular space distorting the architecture and the function of the native tissue.
Heart failure with preserved ejection fraction: Clinical syndrome of heart failure where, although the routinely measured parameters of systolic function are normal, dysfunction of relaxation and passive filling results in heart failure symptoms.
Heart failure with reduced ejection fraction: Clinical syndrome of heart failure where the contractile force is reduced and the proportion of blood expelled with each contraction is below the normal range.
Heart failure: A syndrome of clinical signs and symptoms characterized by the inability of the heart to provide sufficient cardiac output to match the physiological demands of the body.
Hypertensive heart disease: Changes within the heart in response to chronic hypertension which includes cardiomyocyte hypertrophy and fibrosis which can result in heart failure.
Hypertrophic cardiomyopathy: A heart muscle condition, often inherited, resulting in ventricular hypertrophy in the absence of abnormal loading conditions.
Interstitial fibrosis: The accumulation of ECM in the absence of large‐scale cell death in the interstitial and perivascular spaces.
Myocardial infarction: Myocardial damage caused by insufficient blood supply to a myocardial region leading to cell death and fibrotic scar formation
Myofibroblast: Activated fibroblasts with a highly secretory and contractile phenotype which are responsible for the majority of ECM production in pathological states.
Oxidative stress: An imbalance between the production of reactive oxygen species within a tissue and its ability to clear these with antioxidants.
Replacement fibrosis: The deposition of ECM to replace dead or damaged cells and preserve the structural integrity of a tissue.
Transverse aortic constriction: An experimental surgical animal model of pressure overload involving creation of a stenosis in the transverse aorta causing increased pressure in the left ventricular cavity, resulting in hypertrophy, fibrosis and—ultimately—heart failure.
Ventricular remodelling: The changes that occur within the ventricular myocardium in response to pressure or volume overload leading to cardiomyocyte hypertrophy and accumulation of fibrotic tissue.

## Introduction

Heart failure (HF) is a major public health problem with an estimated worldwide prevalence of over 23 million (Bui *et al*, 2011; Ponikowski *et al*, 2014)—a figure projected to rise as populations age (Conrad *et al*, 2018). Despite advances in the treatment of HF over the last decades, mortality and morbidity remain high and HF is a major contributor to the global health economic burden (Lesyuk *et al*, 2018). It is increasingly clear that cardiac fibrosis plays a role in the aetiology of all forms of HF and in particular the pathophysiology of HF with preserved ejection fraction (HFpEF) (Moreo *et al*, 2009; González *et al*, 2018).

Fibrosis is an evolutionarily conserved physiological process intended to repair, replace and reinforce severely or chronically injured tissue when tissue regenerative and homeostatic mechanisms are exhausted. Fibrosis ultimately results in the accumulation of extracellular matrix (ECM) at the site of injury and the production of a “scar”. Short‐term fibrotic processes can be adaptive, but persistent activation of fibrotic pathways, as occur in HFpEF, results in excess accumulation of ECM and disruption of tissue function (Rockey *et al*, 2015). Much research has been carried out in the field of cardiovascular fibrosis, identifying potential antifibrotic targets that may provide new strategies to treat HF. In this review, we summarize the processes involved in the development of HFpEF focussing on interstitial cardiac fibrosis and its contribution to HF, discuss strategies which have been implemented to date—with limited success—and highlight the new opportunities.

## HFpEF

The classical definition of HF is “an inability of the heart to pump blood to the body at a rate commensurate with its needs, or to do so only at the cost of high filling pressures” (Braunwald, 1988) which presents clinically as a syndrome of exertional breathlessness, peripheral oedema and fatigue. HF can be further categorized as HF with reduced ejection fraction (HFrEF) or HFpEF (Ponikowski *et al*, 2016). In HFrEF, the systolic force generation of the heart is impaired, and consequently, the proportion of blood expelled with each contraction—the ejection fraction—is reduced. In HFpEF, routine parameters of systolic function are largely maintained but diastolic filling and relaxation are impaired (Ponikowski *et al*, 2016).

While several therapies exist for HFrEF—including beta‐blockers, drugs targeting the renin–angiotensin–aldosterone system (RAAS) and sodium‐glucose co‐transporter 2 (SGLT2) inhibitors—no treatment has yet been shown to be effective for HFpEF despite multiple randomized trials (Ponikowski *et al*, 2016; Pfeffer *et al*, 2019; Seferovic *et al*, 2019). This is of particular concern as HFpEF is common—estimated to be responsible for > 50% of HF cases (Vasan *et al*, 2018)—and is growing increasingly more so due to its association with ageing and comorbidities such as diabetes, renal dysfunction, hypertension, non‐alcoholic fatty liver disease and sarcopenia (Bekfani *et al*, 2016; Dunlay *et al*, 2017; Streng *et al*, 2018).

Echocardiographic and invasive haemodynamic measurements in HFpEF have described impairment in both diastolic function (Kasner *et al*, 2011; Hummel *et al*, 2017) and in non‐classical measures of systolic function, such as longitudinal contraction (Kraigher‐Krainer *et al*, 2014). Characteristic changes in diastolic function include impairment of ventricular relaxation (Zile *et al*, 2004) resulting in reduced ventricular compliance and reduced efficiency of ventricular filling during diastole (Hay *et al*, 2005; Westermann *et al*, 2008). Maintenance of adequate stroke volume in this setting necessitates elevation of ventricular filling pressures particularly during exercise when diastolic filling time is limited (Westermann *et al*, 2008). Neurohormonal systems including the sympathetic and RAAS are activated which promotes salt and water retention in the kidney. Over time, the increased circulating volume and high levels of AngII and aldosterone are maladaptive, increasing ventricular stretch, oncostatic pressure in the lungs and peripheries and exerting a potent prohypertrophic and profibrotic effect within the myocardium (Díez, 2004; Brown, 2013).

**Figure 1:**
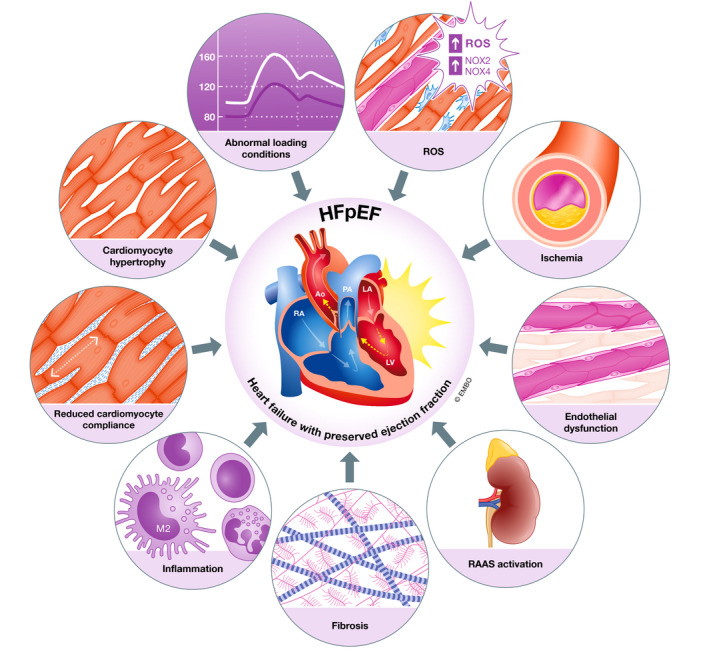
Illustation of the multiple interacting pathophysiological mechanisms responsible for development of HFpEF The development of HFpEF has multiple contributing pathophysiological mechanisms acting on the heart. The relative contribution of each of these factors varies depending on the underlying disease state; however, cardiac fibrosis is a common pathway present in almost all patients with symptomatic HFpEF.

HFpEF does not represent a single pathological process but is a complex disease, with many contributing pathophysiological mechanisms (Cohen *et al*, 2020) both within the cardiomyocyte, in the surrounding tissue (Fig 1) and in peripheral tissues (not discussed here). Endothelial dysfunction has repeatedly been associated with development of HFpEF and is predictive of diastolic dysfunction and subsequent HFpEF in asymptomatic patients (Yang *et al*, 2020). Healthy endothelium releases nitric oxide (NO) which is a key homeostatic mediator with effects on vascular smooth muscle cells, cardiomyocytes and fibroblasts and has been suggested to be a cornerstone of HFpEF pathophysiology (Paulus & Tschöpe, 2013). Cardiomyocyte hypertrophy (Takimoto *et al*, 2005), increased myocardial stiffness (Bishu *et al*, 2011) and cardiac fibrosis (Calderone *et al*, 1998) have all been associated with reduced NO signalling through decreased cyclic GMP production and inhibition of protein kinase G activity in various cell types (Paulus & Tschöpe, 2013).

Dysfunctional endothelial cells also express high levels of vascular adhesion molecules which promote the migration of inflammatory cells into the myocardium (Westermann *et al*, 2011). Inflammatory cell infiltration is augmented by local release of inflammatory mediators including IL‐1, IL‐6 and TNF‐ɑ in response to hypoxia or local tissue damage (Turner *et al*, 2007; Yu *et al*, 2012). Systemic inflammatory conditions including rheumatological conditions, diabetes or metabolic syndrome which are associated with HFpEF prime immune cells to initiate exaggerated inflammatory and fibrotic responses when recruited to tissues (Esposito *et al*, 2002; Umare *et al*, 2014). Inflammation within the myocardium increases oxidative stress, reduces cGMP production, damages the endothelium and impairs cardiomyocyte performance (Picchi *et al*, 2006; Waddingham *et al*, 2019). If persistent, inflammation can be associated with the emergence of profibrotic macrophages (Westermann *et al*, 2011; Peet *et al*, 2019) and infiltration of Th1 T cells (Nevers *et al*, 2017). These inflammatory cells express transforming growth factor β (TGFβ), interferon‐ɣ, Galectin‐3 (Gal‐3), connective tissue growth factor and angiotensin‐converting enzymes which activate cardiac fibroblasts (CF) thereby promoting the deposition of ECM and the occurrence of fibrosis.

At the level of cardiomyocytes, mechanical stretch, neurohormonal activation and oxidative stress lead to a hypertrophic response with increased sarcomere numbers, cardiomyocyte area, myocardial mass and impaired relaxation kinetics (Kojima *et al*, 1994; Okoshi *et al*, 2004). Post‐translational modification to sarcomeric proteins such as phosphorylation of titin occurs in response inflammatory and profibrotic signals which reduces the compliance of the cardiomyocyte during relaxation (Fukuda *et al*, 2005; Krüger *et al*, 2009). Oxidative stress within the heart is elevated in HFpEF (Vitiello *et al*, 2014) particularly in conditions such as obesity, hypertension and diabetes which can cause mitochondrial dysfunction (Sverdlov *et al*, 2016; Sorop *et al*, 2018), uncouple the electron transport chain (Boudina *et al*, 2007), upregulate reactive oxygen species (ROS)‐producing enzymes (Ide *et al*, 2000; Moris *et al*, 2017) and reduce antioxidant activity (Ballal *et al*, 2010). Oxidative stress impacts NO signalling, the phosphorylation state of sarcomeric proteins, calcium handling and hypertrophy within the cardiomyocyte. This results in increased myocardial stiffness, impaired energetic metabolism and a profibrotic, pro‐inflammatory secretome which contributes to and perpetuates the haemodynamic changes of HFpEF.

### Fibrosis in HFpEF

Among the multiple factors contributing to the development of HFpEF, fibrosis is a common pathway which exists regardless of aetiology. In patients with symptomatic HFpEF, extracellular fibrotic burden is more strongly correlated with diastolic dysfunction than is cardiomyocyte stiffness (Zile *et al*, 2015) and fibrosis is correlated with increased arrhythmias (Cho *et al*, 2018) hospitalization and mortality in HFpEF (Kanagala *et al*, 2019) making it an attractive therapeutic target. There is a prolonged asymptomatic phase prior to the development of HFpEF in which significant structural and haemodynamic changes accumulate within the heart but without limiting symptoms (Abhayaratna *et al*, 2006; Kosmala & Marwick, 2020). Although reducing cardiomyocyte stiffness, endothelial dysfunction and oxidative stress may provide beneficial effects in the early stages of disease, reversing fibrotic changes and positively remodelling the myocardium is crucial to improve cardiac function and ameliorate symptoms late in the disease as symptoms are beginning to develop (Kim *et al*, 2018).

Fibrotic tissue is predominantly composed of fibrillar collagens such as collagen I and collagen III which strongly influence the biomechanical properties of the ECM. Fibrillar collagens have high tensile strength providing structural support to the myocardium, however when present in excess, reduces myocardial compliance (de Souza, 2002). Collagen subtypes have differing elastic properties, and therefore, the ratio between collagen subtypes in addition to increased quantity is important for the physiological effects seen in the fibrotic heart. Collagen I accounts for 85–90% of collagen within the healthy heart with collagen III making up 5–10% and smaller contributions from other collagen subtypes (Weber, 1989; de Souza, 2002). Collagen I is less compliant when exposed to tension compared to collagen III which has more elastic properties (Collier *et al*, 2012; Asgari *et al*, 2017). An increased ratio of type I vs. type III collagens is seen in both animal and human models of pressure overload (Kasner *et al*, 2011; López *et al*, 2014; Echegaray *et al*, 2017) and is correlated with worsening diastolic function and increased symptoms (Kasner *et al*, 2011).

#### Fibrosis histology

**Figure 2:**
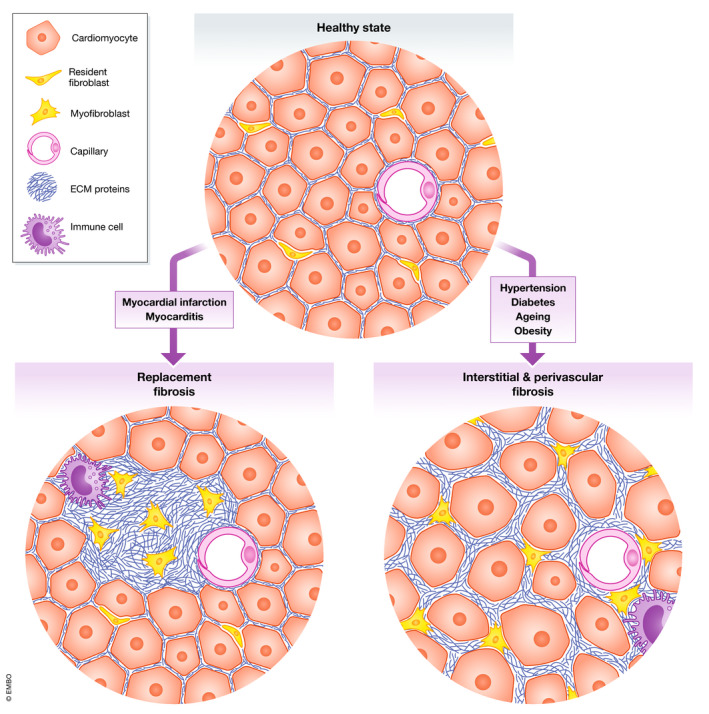
Histological differences between replacement fibrosis and interstitial/perivascular fibrosis Illustration of replacement vs. interstitial/perivascular fibrosis showing the differential spatial accumulation of ECM in the two forms of cardiac fibrosis along with altered cellular architecture, cardiomyocyte hypertrophy, inflammatory cell infiltration and activation of myofibroblasts.

Fibrotic changes in the heart can be broadly categorized into distinct but not mutually exclusive categories of (i) replacement or (ii) reactive/interstitial fibrosis (Fig 2). Replacement fibrosis is classically associated with myocardial infarction (MI) where cardiomyocyte cell death and muscle loss are replaced by ECM proteins to maintain the structural integrity of the heart wall. This is a crucial process to reinforce areas of the myocardium weakened by cardiomyocyte loss and prevent myocardial rupture. The resulting area of fibrotic scar is non‐contractile, non‐elastic tissue that does not contribute to force generation. Thus the size, composition and physical properties of the fibrotic scar have major implications for the development of HF.

So‐called “reactive fibrosis” is an alternative form of cardiac fibrosis which occurs in the absence of large‐scale cardiomyocyte death and will be the focus of the remainder of this review. There are two major histologically distinct forms of reactive fibrosis—interstitial and perivascular—which often coexist. Interstitial fibrosis involves the deposition of collagen‐rich ECM in the interstitial space between cells and is most commonly associated with chronic stressors that include abnormal loading conditions (e.g. hypertension, post‐MI or valve pathology) (Brilla *et al*, 2000; Treibel *et al*, 2018a) or profibrotic systemic conditions (Shimizu *et al*, 1993; Eschalier *et al*, 2014; Kobayashi *et al*, 2017). Perivascular fibrotic tissue is rich in inflammatory cell infiltrate and is more prominent in conditions where endothelial damage predominates such as hypertensive heart disease (HHD) or diabetes (Hinglais *et al*, 1994; López *et al*, 2006). ECM production in perivascular fibrosis may have a greater role for endothelial to mesenchymal transition (Endo‐MT; a debated process) (Zeisberg *et al*, 2007; Okayama *et al*, 2012), fibroblastic differentiation of pericytes (Kramann *et al*, 2015) and infiltration of inflammatory cells (Hinglais *et al*, 1994; Hara *et al*, 2002; Nevers *et al*, 2017). Perivascular fibrosis is also associated with abnormalities in coronary blood flow (Dai *et al*, 2012), and increased diffusion distance from the endothelium to cardiomyocytes reduces the diffusion of oxygen, fatty acids, glucose and signalling molecules such as NO (Nevers *et al*, 2017). However, differentiating the effects of interstitial and perivascular fibrosis is challenging as these processes typically coexist and for technical reasons are normally grouped together in analysis.

In human disease, myocardial interstitial fibrosis typically builds up over many years before manifesting clinically, leading to the important question of its reversibility. Fortunately, fibrosis does not exist as an inert, metabolically inactive tissue but rather undergoes continual remodelling controlled by the activity of fibroblasts, immune cells and proteolytic enzymes. The removal of pressure overload in animal studies results in positive myocardial remodelling with reduced interstitial collagen (Walther *et al*, 2001; Szardien *et al*, 2012). Similarly, human studies in patients with aortic stenosis have demonstrated a gradual reduction in interstitial cardiac fibrosis following aortic valve replacement (Villari *et al*, 1995; Treibel *et al*, 2018b). This positive remodelling has also been replicated using RAAS pathway inhibitors in hypertensive heart disease with associated improvement in cardiac haemodynamics (Brilla *et al*, 2000; Díez *et al*, 2002). Replacement fibrosis, unlike diffuse fibrosis, was not shown to resolve following treatment of aortic stenosis (Treibel *et al*, 2018b) which may be partly explained by increased collagen cross‐linking within areas of replacement fibrosis which render the tissue resistant to collagenase mediated degradation (Frangogiannis, 2019). Interstitial fibrosis tends to be less heavily cross‐linked than replacement fibrosis but increased cross‐linking of interstitial fibrosis is associated with diabetes (Liu *et al*, 2003) and hypertension (Norton *et al*, 1997; Badenhorst *et al*, 2003; López *et al*, 2016). High levels of collagen cross‐linking have been suggested as an explanation for lack of efficacy in trials of antifibrotics in HFpEF (Ravassa *et al*, 2018). Therefore, early identification and treatment of patients with HFpEF and cardiac fibrosis may be important for achieving optimal outcomes.

#### Monitoring of cardiac fibrosis in clinic

Given the heterogeneous pathophysiology of HFpEF, the ability to detect and monitor changes in fibrosis over time is optimal for identifying patients most likely to benefit from antifibrotic interventions and define the effectiveness of these treatments in clinical trials. The gold standard measure of cardiac fibrosis is histological analysis of endomyocardial biopsy (EMB) samples stained for collagen using picrosirius red under polarized light (Whittaker *et al*, 1994). However, a number of limitations make this method impractical for routine use. Primarily, EMB samples only a small fraction of the myocardium which can lead to sampling error depending on the site of the biopsy and the site of pathology. EMB is an invasive procedure and associated with procedural risks (From *et al*, 2011). The safest and most accessible site for EMB is the right side of the interventricular septum which may not correlate with left ventricular pathology.

Surrogate markers have been developed to non‐invasively quantify myocardial fibrosis and overcome some of the limitations of EMB. During ECM synthesis, collagen is released as a promolecule requiring cleavage of the amino and carboxyl‐terminals by collagen peptidase to form mature collagen fibrils. These cleaved terminal peptides can be measured in serum to give an indication of the quantity of collagen formation. To date, the carboxyl‐terminal of procollagen I (PICP) has shown the most promise in HFpEF. PICP is associated with raised collagen content on EMB, diastolic dysfunction and prognosis in HFpEF (Querejeta *et al*, 2000; López *et al*, 2015a,b). Alternative markers of collagen synthesis including the amino‐terminal of procollagen I (PINP) and III (PIIINP) have been detected in serum and are elevated in hypertensive patients (Díez *et al*, 1995) and those with hypertrophic cardiomyopathy (HCM) (Lombardi *et al*, 2003). However PINP and PIIINP have not been convincingly associated with histological measures of cardiac collagen in HFpEF (López *et al*, 2015a,b). Similarly, the C‐terminal telopeptide produced during the degradation of collagen I (CITP) can be measured in serum to provide a surrogate measure of collagen degradation which has shown an association with HFpEF symptoms (Martos *et al*, 2009). However, the extent to which these biomarkers, when measured peripherally, are representative of changes in myocardial collagen content is unclear. Studies sampling directly from the coronary sinus (CS) have yielded conflicting results regarding the cardiac contribution to the circulating levels of terminal peptides. PICP is elevated in CS samples from HHD patients (Querejeta *et al*, 2004); however, CS measurement of PINP, PIIINP and CITP do not show association with the burden of fibrosis measured by histology or cardiac magnetic resonance imaging (CMR) (Kupari *et al*, 2013; Nagao *et al*, 2018). Peripheral measurements of collagen biomarkers are more likely to represent a systemic profibrotic or inflammatory state than to specifically reflect the level of cardiac fibrosis. In spite of this, given the multisystemic nature of HFpEF, this does not preclude these markers from having role in prognosticating disease or guiding therapy (Krum *et al*, 2011) but caution must be exercised when drawing conclusions about cardiac collagen content from use of these biomarkers, and currently, they remain a research tool.

Advances in CMR have made the non‐invasive quantification and localization of fibrosis within the myocardium possible, and CMR compares favourably with histological measures (Diao *et al*, 2016). CMR has many potential advantages compared to invasive histology based assessment; sampling errors seen with EMB are reduced as the entire myocardium is imaged, CMR is non‐invasive and low risk allowing serial scans in the same patient to track progression or resolution of fibrosis over time, and structural and functional information can be collected as part of the same study.

Late gadolinium contrast enhancement (LGE) imaging has been used extensively to identify focal areas where volume of distribution is increased and contrast washout delayed which correlates with fibrotic areas histologically (Schelbert *et al*, 2010). Although LGE is primarily detects areas of replacement fibrosis, this still correlates with cardiac function in HFpEF across aetiologies including HHD (Rudolph *et al*, 2009; Krittayaphong *et al*, 2010), aortic stenosis (Nigri *et al*, 2009; Everett *et al*, 2020), diabetic cardiomyopathy (Kwong *et al*, 2008) and HCM (Bruder *et al*, 2010; Moravsky *et al*, 2013).

More recently, CMR measurement of the interstitial component of cardiac fibrosis has become possible due to the advent of T1 mapping (Löffler *et al*, 2019). T1 mapping uses precontrast and post‐contrast magnetic resonance measurements from the myocardium to provide a quantitative assessment of the extracellular volume (ECV) within an area of myocardium. ECV is highly correlated with collagen content measured histologically (Diao *et al*, 2016; Duca *et al*, 2016), functional measures of diastolic function (Rommel *et al*, 2016) and prognosis in HFpEF (Mascherbauer *et al*, 2013; Schelbert *et al*, 2017). Clinical trials of antifibrotic HFpEF therapies are starting to use CMR‐derived ECV measures in patient selection and as an outcome measure (Lewis *et al*, 2019). This may begin to address some of the long‐standing issues with patient selection for HFpEF clinical trials (Kelly *et al*, 2015) ensuring these therapies are targeted at the group of patients most likely to benefit.

## Cellular and molecular mechanisms of fibrosis

### Myofibroblasts—the cellular driver of fibrosis

A central event in the development of fibrotic changes within the heart is the accumulation of activated myofibroblasts at the site of injury (Moore‐Morris *et al*, 2014; Rockey *et al*, 2015; Kanisicak *et al*, 2016). Myofibroblasts exhibit two cardinal features: firstly, they secrete large amounts of ECM components, and secondly, via the expression of smooth muscle actin (SMA, otherwise known as ACTA2), they are contractile (Wynn, 2008; Rosenbloom *et al*, 2017). Together, these features result in the expansion of ECM, increased tissue stiffness and ventricular remodelling typical of cardiac fibrosis and HF.

The cellular origin of myofibroblasts *in vivo* has been debated extensively (Di Carlo & Peduto, 2018) with multiple candidates having been identified in the literature including epicardial or endothelial cells undergoing mesenchymal transition or migration of hematopoietic cells or pericytes into the interstitium (van Amerongen *et al*, 2008; Krenning *et al*, 2010; Widyantoro *et al*, 2010). Lineage tracking studies of myofibroblasts using periostin as a marker of myofibroblast activation have found that resident cardiac fibroblasts (CF) in the myocardium are the most significant contributor to the myofibroblasts population in cardiac injury with minimal input from extracardiac sources or endothelial structures (Acharya *et al*, 2012; Moore‐Morris *et al*, 2014; Kanisicak *et al*, 2016).

Recent cell sorting experiments suggest that resident CF make up approximately 10% of the total cell number within the heart which is significantly less than previous estimates when fibroblasts were considered to be the most abundant cell within the heart (Banerjee *et al*, 2007; Pinto *et al*, 2016; Tallquist & Molkentin, 2017). This lack of consensus on the origin, definition and relative proportion of ECM‐producing cells within the heart is in part due to the extensive heterogeneity among this cell type. Significant variation in synthetic function, morphology and gene expression exists among CF depending upon their origin, anatomical site within the heart and state of activation. A variety of cell surface and intracellular markers has been used to identify these cells including fibroblast‐specific protein (FSP1), DDR2, Sca‐1, Thy‐1, fibronectin and vimentin (Hudon‐David *et al*, 2007; Ivey & Tallquist, 2016). However, no single marker is sufficiently comprehensive or specific (Kong *et al*, 2013), and as a result, combinations of cell markers have been used to define CF, although likely incompletely.

Two markers which appear to be relatively more specific to CF are the platelet‐derived growth factor receptor α (PDGFRα) and the transcription factor Tcf21, both of which are expressed in the majority of myofibroblast‐forming cells (Smith *et al*, 2011; Acharya *et al*, 2012; Pinto *et al*, 2016). PDGFR‐**α** and Tcf21 are both expressed in the epicardial layer of the developing heart and are necessary for epithelial to mesenchymal transition of epicardial cells. Genetic knockout of Tcf21 leads to a paucity of CF within myocardium, and lineage tracking studies suggest an epicardial origin for a majority of resident CF (Smith *et al*, 2011; Acharya *et al*, 2012). An endothelial origin has been identified for a smaller proportion of resident CF using the endothelial marker Tie2 (Moore‐Morris *et al*, 2014) which comprise approximately 10% of the left ventricular fibroblasts and have indistinguishable behaviour in response to cardiac injury (Acharya *et al*, 2012; Tallquist & Molkentin, 2017).

Although activation of myofibroblasts is of central importance in most profibrotic settings, recent evidence—particularly in the setting of metabolic disease, has suggested that increased ECM synthesis may occur in the absence of myofibroblast activation. High glucose‐containing media increases collagen production from CF (Zhang *et al*, 2007; Gu *et al*, 2017) without myofibroblast activation. Animal studies in diabetic mice have indicated that ECM deposition is upregulated independently of myofibroblast activation and α‐SMA expression and is not dependent on TGFβ stimulation (Alex *et al*, 2018). Furthermore, fibroblasts isolated from the atria of humans with type 2 diabetes have increased expression of collagen I in the absence of TGFβ stimulation (Sedgwick *et al*, 2014) indicating that CF from diabetic individuals may possess an inherently profibrotic phenotype.

### Molecular processes in fibrosis

The molecular processes involved in fibrosis are expansive, complex and interacting, and a comprehensive description is beyond the scope of this review. However, certain groups of factors are frequently implicated in fibrosis and have consequently been explored as potential therapeutic targets. Chief among these is the TGFβ family of proteins, which are potent drivers of fibroblast‐to‐myofibroblast transition and powerful stimuli for ECM synthesis (Meng *et al*, 2016).

More recently, a search for factors acting downstream of TGFβ1 led to the re‐evaluation of interleukin‐11 (IL11) as a profibrotic molecule (Schafer *et al*, 2017). Similarly, Gal‐3 (Shen *et al*, 2018) and processes resulting in oxidative stress have emerged as new antifibrotic targets (Somanna *et al*, 2016). A new and alternative paradigm has been to target the fibroblast itself, aiming to deplete myofibroblast populations (Aghajanian *et al*, 2019). Below, we describe attempts to produce antifibrotic therapies against the more established targets of the TGFβ family and vasoactive peptides before reviewing more novel targeting of IL11, Gal‐3, oxidative stress or the activated myofibroblast itself.

## Established targets

### TGFβ inhibitors—effective but toxic

The TGFβ family of proteins are the most well established and potent activators of profibrotic effects in cells, have been implicated in virtually all forms of fibrosis and have been described as the “master regulators” of fibrosis (Meng *et al*, 2016). TGFβ is a ubiquitously expressed protein with an expansive range of biological effects including enhanced ECM synthesis, cell differentiation, apoptosis, angiogenesis and immune cell function depending on the site of action. Myocardial TGFβ expression is consistently upregulated in the heart of patients with HFpEF irrespective of aetiology including HCM (Li *et al*, 1997), HHD (Almendral *et al*, 2010) and aortic stenosis (Hein *et al*, 2003). There are three isoforms of TGFβ of which TGFβ1 is the most well studied in the context of fibrosis. TGFβ is secreted as an inactive peptide due to the presence of the bound latency‐associated peptide (LAP) which prevents it accessing its receptor (Taipale *et al*, 1994) and is further sequestered into the structure of the ECM by latency TGFβ binding proteins. Dissociation of LAP from TGFβ is an important step in the profibrotic response which occurs in response to tissue damage, inflammation or profibrotic signals and is mediated by a variety of factors which are upregulated in patients with HFpEF. These factors include MMP‐2, MMP‐9, ADAMTS16 and plasmin which have protease activity that cleaves LAP from the TGFβ molecule (Khalil *et al*, 1996; Yu & Stamenkovic, 2000; Wang *et al*, 2006a; Yao *et al*, 2020). Thrombospondins (Reed *et al*, 1995), ROS (Barcellos‐Hoff & Dix, 1996) and specific integrins (Munger *et al*, 1999; Wipff *et al*, 2007) induce a conformational change in the LAP which exposes the receptor binding site on the TGFβ molecule. Constituently, active forms of TGFβ, resistant to LAP inactivation, have been used in both large and small animal models to stimulate atrial and/or ventricular cardiac fibrosis resulting in HF, arrhythmias and reduced survival (Nakajima *et al*, 2000; Verheule *et al*, 2004; Accornero *et al*, 2015; Polejaeva *et al*, 2016).

Cellular signalling of TGFβ in fibrosis is mediated by the membrane‐bound TGFβRII and can involve the canonical, SMAD‐dependent pathway or non‐canonical, SMAD‐independent pathway. In the SMAD‐dependent pathway, TGFβ binding induces the formation of a heterotetrameric complex between with TGFβRII and TGFβRI (also known as ALK‐5) molecules. Formation of this complex activates the phosphorylation activity of the receptor and activates SMAD2 and SMAD3 (Wells *et al*, 1999), which dissociate from the receptor and complex with SMAD4 in the cytoplasm (Derynck & Zhang, 2003). This SMAD complex translocates to the nucleus where it binds to SMAD binding elements in the genome to act as a transcription factor independently (Dennler *et al*, 1998; Martin‐Malpartida *et al*, 2017) or in combination with multiple other transcription factors (Zhang *et al*, 1998; Mullen *et al*, 2011). Within the fibroblast, activation of the canonical pathway results in myofibroblast differentiation (Khalil *et al*, 2017) and upregulation of multiple profibrotic genes including collagen, smooth muscle actin and periostin, along with IL11 (Schafer *et al*, 2017). Non‐canonical signalling mediates similar profibrotic effects in fibroblasts (Chen *et al*, 2005; Dolivo *et al*, 2019), and significant cross‐talk exists between these pathways (Engel *et al*, 1999; Funaba *et al*, 2002). The mitogen‐activated protein kinase (MAPK) pathways—including ERK (Lee *et al*, 2007), p38 (Molkentin *et al*, 2017) and JNK (Yoshida *et al*, 2005)—are chief mediators of this non‐canonical response. Blocking this signalling using transgenic mice or specific inhibitors of the MAPK pathways reduces myofibroblast formation and ECM production (Gao *et al*, 2013; Xu *et al*, 2017).

TGFβ signalling within the cardiomyocyte also has effects on cardiac remodelling particularly via the non‐canonical p38 pathway (Gao *et al*, 2013; Xu *et al*, 2017) which influences release of profibrotic mediators in response to stress (Koitabashi *et al*, 2011) and upregulates genes related to cardiomyocyte hypertrophy (Matsumoto‐Ida *et al*, 2006). The canonical TGFβ signalling pathway within cardiomyocytes provides important survival signals and maintains contractility during cell stress (Wang *et al*, 2005; Umbarkar *et al*, 2019) which may underlie toxicity when inhibiting this pathway.

*In vivo*, transgenic mice with fibroblast‐specific disruption of TGFβR1/2 or SMAD3 protect against cardiac fibrosis and improve diastolic function in response to transverse aortic constriction (TAC) (Khalil *et al*, 2017). Similarly, mice with a single functional allele of the TGFβ gene are relatively resistant to age‐related cardiac fibrosis and have an increased lifespan compared to wild‐type mice (Brooks & Conrad, 2000) suggesting a potential therapeutic target. These findings have been replicated in preclinical studies over the last two decades employing either neutralizing monoclonal antibodies (mAb) against TGFβ (Kuwahara *et al*, 2002; Teekakirikul *et al*, 2010) or small molecule kinase inhibitors targeting TGFβRI (Kuwahara *et al*, 2002; Derangeon *et al*, 2017) which have markedly reduced cardiac fibrosis and improved LV compliance in rodent models of HFpEF. However, the multifunctional role of TGFβ provides a significant challenge—repeatedly encountered with anti‐TGFβ therapy in both animal and human studies—of on‐target toxicities that are dose‐limiting thus hindering treatment efficacy.

The problems encountered with anti‐TGFβ therapies are highlighted in *Tgfb1* knockout (KO) mice and humans. Mice have high embryonic lethality and those that survive to birth die between 3 and 5 week of age due to an excessive and widespread inflammatory response resulting in multiorgan failure (Shull *et al*, 1992; Kulkarni *et al*, 1993). In humans, biallelic loss of function mutations of TGFB1 gene results in severe colitis and neurological deficits with frequent seizures and cerebral atrophy (Kotlarz *et al*, 2018). Cardiovascular toxicity is common in trials targeting TGFβ which may relate, in part, to the protective role played by TGFβ in the cardiomyocyte. Umbarkar *et al*, using a tamoxifen inducible cardiomyocyte‐specific KO of *Smad4*, found that these mice developed LV dilation and HF following tamoxifen treatment (Umbarkar *et al*, 2019). Similar effects have been seen with global inhibition of the TGFβ pathway using mAbs (Frantz *et al*, 2008; Koitabashi *et al*, 2011), kinase inhibitors (Engebretsen *et al*, 2014) or global *Smad3* KO animals (Divakaran *et al*, 2009). Although surviving animals have reduced cardiac fibrosis in response to MI or TAC, in the acute phase there are increased rates of LV dilation and increased mortality (Divakaran *et al*, 2009).

Human studies have been undertaken using fresolimumab—a mAb which neutralizes all three isoforms of TGFβ; however, this has shown minimal benefit in renal fibrosis or scleroderma and was associated with the development of keratoacanthomas in the skin (Lacouture *et al*, 2015; Rice *et al*, 2015; Vincenti *et al*, 2017). Additionally, other pan‐TGFβ neutralizing antibodies have been associated with fatal haemorrhage in both mice and non‐human primates and have again demonstrated significant cardiovascular toxicity with inflammatory infiltration of the coronary vessels, aorta and heart valves (Mitra *et al*, 2020). Selective targeting of the TGFβ1 isoform has not improved the toxicity profile with dose‐related effects seen on epithelial hyperplasia, enteropathy and renal tubular inflammation in non‐human primate studies (Brennan *et al*, 2018). When translated to humans, lower doses were tolerated in trials of diabetic nephropathy; however, this was at the expense of efficacy as progression of renal dysfunction was not reduced (Voelker *et al*, 2017). Small molecule inhibitors targeting the TGFβRI have produced similar toxicity concerns including degenerative changes of heart valves and aorta and mucosal inflammation in the intestine (Anderton *et al*, 2011; Stauber *et al*, 2014). Recently, a therapeutic window has been identified for the use of the TGFβRI inhibitor, galunisertib, in the treatment of myelodysplasia; however, this requires a stringent intermittent dosing schedule to avoid cardiac toxicity (Kovacs *et al*, 2015; Santini *et al*, 2019) and efficacy as an antifibrotic has not been tested.

In summary, TGFβ signalling is strongly and irrefutably profibrotic *in vitro* and *in vivo* and targeting this pathway directly or indirectly has potent antifibrotic effects. However, the toxicity profile associated is consistently too high and is sufficiently dose‐limiting to render the treatment ineffective for human disease, to date.

### Renin/angiotensin/aldosterone system inhibitors—mainstay therapy in heart failure

Targeting the RAAS has been a pillar of HF treatment for over 30 years and is perhaps the seminal success of modern day disease‐modifying therapy in HFrEF (Swedberg, 1987; Pfeffer *et al*, 1992; Pitt *et al*, 1999). Classically, angiotensin II (AngII) is a profibrotic circulating factor produced by the serial actions of renin‐ and angiotensin‐converting enzymes (ACE) on angiotensinogen. In CF, AngII treatment stimulates myofibroblast formation and ECM production (Brilla *et al*, 1994; Siddesha *et al*, 2013). AngII signalling in the heart is mediated through G protein‐coupled receptors (GPCR) designated angiotensin receptor type 1 (AT1R) and type 2 (AT2R). The intracellular effect of AngII involves Gq‐mediated activation of phospholipase C, β‐arrestin signalling and transactivation of other membrane‐bound growth factor receptors. AT1R‐mediated β‐arrestin signalling (McDonald *et al*, 2000; Rakesh *et al*, 2010) and transactivation of PDGFR and epithelial growth factor receptor (EGFR) (Mondorf *et al*, 2000; Schellings *et al*, 2006) have all been shown to activate MAPK pathways in CF (Schorb *et al*, 1995). AT1R stimulation has also been shown to directly augment the SMAD‐dependent pathways of TGFβ signalling (Wang *et al*, 2006b) and in concert with endothelin‐1 (ET‐1) (Fujisaki *et al*, 1995) and largely indirectly—induces hypertrophic changes in cardiomyocytes (Gray *et al*, 1998). In rodents, AngII infusion is a well established *in vivo* model for stimulating cardiac fibrosis and has been used in many 100s of publications (Sun *et al*, 1997). This profibrotic of effect AngII is maintained even at subpressor doses which despite not increasing blood pressure results in fibrosis accumulation and diastolic dysfunction (Regan *et al*, 2015).

AT1R antagonists or ACE inhibitors (ACE‐I) have in multiple settings reduced ECM production, cardiac hypertrophy and HF in both cell culture and animal models (Pahor *et al*, 1991; Brilla *et al*, 1994; Ham *et al*, 2018). In short‐term human studies of HHD, following 6 months of ACE‐I treatment the myocardial collagen content on EMB and echocardiographic features of diastolic function were reduced compared to treatment with antihypertensive alone (Brilla *et al*, 2000). However, multiple clinical trials in HFpEF have failed to demonstrate a mortality benefit or reduction in hospitalization with AngII inhibition, suggesting that this approach is insufficient to block the activity of the multiple profibrotic pathways which are active in HFpEF and suggests a high degree of redundancy within this system (Yusuf *et al*, 2003; Cleland *et al*, 2006; Martin *et al*, 2018).

### Mineralocorticoid receptor antagonists—old drugs but effective

Aldosterone binds to the mineralocorticoid receptor (MR) in the cytoplasm and translocates to the nucleus where it complexes with a variety of co‐activators and is responsible for upregulating profibrotic genes. Additionally, aldosterone has multiple non‐transcriptional dependent effects which occur more rapidly and can occur independently of the MR (Mihailidou *et al*, 2004; Markos *et al*, 2005). In particular, AngII and aldosterone work synergistically to produce a potent profibrotic effect (Lemarié *et al*, 2009). Aldosterone augments AngII signalling by upregulation of MAPK pathways in both cardiomyocytes (Tsai *et al*, 2013; Somanna *et al*, 2015) and CF (Stockand & Meszaros, 2003; Lemarié *et al*, 2009) in a process dependent on G protein‐coupled receptor kinases (Cannavo *et al*, 2016). Aldosterone has been implicated in multiple other processes linked to HFpEF including production of ROS (Hayashi *et al*, 2008) and development of a pro‐inflammatory infiltrate within the heart during pressure overload by promoting differentiation of profibrotic “M2” macrophages (Rickard *et al*, 2009) and infiltration of profibrotic T cells (Li *et al*, 2017).

*In vivo* studies in HFpEF models have been promising with inhibition of this pathway using either cardiomyocyte‐specific KO of the MR (Lother *et al*, 2011; Rickard *et al*, 2012) or specific MR antagonists (MRA) (Nishioka *et al*, 2007; Leader *et al*, 2019) demonstrating reduced ECM production and improved LV function. Mechanistic studies in humans with MRA have mirrored these results showing a reduction in myocardial collagen accumulation histologically or using ECV on CMR (Table 1; Kosmala *et al*, 2011; McDiarmid *et al*, 2020).

**Table 1:** Clinical trials of drugs where mode of action includes the potential to target cardiac fibrosis that is shown here as an endpoint outcome

Treatment	Duration	Population	Measure of fibrosis	*N* Rx vs. placebo	Year	Fibrosis‐related outcome	PMID/NCT
Mineralocorticoid receptor antagonists							
Spironolactone	6 months	HFrEF	PINP/PIIINP	81 vs. 70	2000	Reduced PINP/PIIINP	11094035
Spironolactone	12 months	HFrEF—DCM	PICP CVF on EMB	13 vs. 0	2005	Reduced PICP/CVF	16275882
Spironolactone	3 months	IHD	PIIINP	98 vs. 98	2007	Reduced PIIINP	17921831
Eplerenone	6 months	HFpEF	PINP	22 vs. 22	2011	Reduced PINP Improved diastolic function	21807324
Spironolactone	6 months	HFpEF—obesity	PICP/PIIINP	58 vs. 55	2013	Reduced PICP/PIIINP Improved diastolic function	23343682
Spironolactone	6 months	HFpEF—female	PIIINP	24 vs. 24	2014	Reduced PIIINP Improved diastolic function	24905296
Spironolactone	Variable	HFpEF	PICP	167 vs. 161	2015	Reduced PICP Improved diastolic function	26459931
Canrenone	6 months	HFpEF	PIIINP	197 vs. 197	2017	Negative	28855452
Spironolactone	12 months	HCM	PINP/PIIINP LGE on CMR	26 vs. 27	2018	Negative	29604289
Spironolactone	12 months	HFpEF	PICP	190 vs. 180	2018	Reduced PCIP levels Improved diastolic function	29709099
Spironolactone	6 months	HFpEF	ECV on CMR	19 vs. 21	2019	Negative	31852424
Spironolactone	24 months	HCM	LGE on CMR	130 vs. 130	Ongoing	–	NCT02948998
Angiotensin inhibition							
Lisinopril	6 months	HHD	CVF on EMB	18 vs. 17	2000	Reduced CVF Improved diastolic function	10993857
Losartan	12 months	HHD	CVF on EMB	19 vs. 0	2002	Reduced CVF & improved diastolic function in severe fibrosis	12034658
Losartan	6 months	HFpEF—ESRF	PICP	13 vs. 13	2005	Reduced PICP	16471172
Enalapril	6 months	HFpEF—ESRF	PICP	13 vs. 13	2005	Negative	16471172
Irbesartan	12 months	HHD	PICP	56 vs. 58	2007	No difference PICP Improved diastolic function	17762662
Irbesartan	6 months	HFpEF	PIIINP	149 vs. 164	2011	Negative	21750125
Candesartan	3‐4 months	HFrEF—Anthracycline	ECV on CMR	38 vs. 32	2018	Reduced ECV	29106497
Ramipril	36 months	ARVC	MMP, TIMP	60 vs. 60	Ongoing	–	29574980
Valsartan	24 months	HCM	ECV on CMR	75 vs. 75	Ongoing	–	28454798
Vasodilators							
Sildenafil	6 months	HFpEF	PIIINP	113 vs. 103	2014	Negative	23478662
Isosorbide dinitrate	6 months	HFpEF	ECV on CMR	13 vs. 16	2017	Negative	28219917
Isosorbide dinitrate + Hydralazine	6 months	HFpEF	ECV on CMR	15 vs. 16	2017	Negative	28219917
Neprilysin inhibitors							
Sacubitril‐Valsartan	9 months	HFpEF	PIIINP/MMP2	149 vs. 152	2016	Negative	26754625
Diuretics							
Torasemide	8 months	HFrEF	CVF on EMB PICP	19 vs. 17	2004	Reduced in CVF Reduced PICP	15172408
Torasemide	8 months	HFpEF	PICP	77 vs. 78	2011	Negative	21906812
Torasemide	9 months	HFpEF	PICP	17 vs. 18	2017	Negative	28891228
Antihyperglycaemic							
Empagliflozin	6 months	T2DM	ECV on CMR	35 vs. 0	2019	Negative	31653956
Dapagliflozin	12 months	T2DM	ECV on CMR	30 vs. 30	Ongoing	–	NCT03782259
Metformin	24 months	HFpEF (metabolic syndrome)	TIMP1	27 vs. 27	Ongoing	–	24515256
Statins							
Rosuvastatin	6 months	HFrEF	PINP/PIIINP	32 vs. 37	2011	Negative	20085851
Atorvastatin	6 months	HFrEF	PIIINP	28 vs. 28	2012	Reduction in PIIINP levels	22154198
Other							
Vitamin D3	6 weeks	HFrEF	PINP/PIIINP	50 vs. 51	2013	Negative	23895820
Mirabegron	12 months	HFpEF	ECV on CMR	148 vs. 148	Ongoing	–	29932311
Supervised exercise program	4 months	Mixed	ECV on CMR	60 vs. 30	Ongoing	–	NCT03084679
Pirfenidone	12 months	HFpEF	ECV on CMR	65 vs. 65	Ongoing	–	31069575
Stem cell therapy							
Allogenic Mesenchymal Stromal Cells	12 months	HFrEF—Anthracycline	ECV on CMR	21 vs. 15	Ongoing	–	29910056

ARVC, arrhythmogenic right ventricular cardiomyopathy; CVF, collagen volume fraction; LGE, late gadolinium enhancement; NCT, ClinicalTrials.gov registry number; PMID, PubMed identification number; TIMP, tissue inhibitor of metalloproteases.

However—as with ACE‐I—rodent and intermediate phenotype studies have so far failed to translate to improved outcomes in large randomized controlled trials (RCT) of MRA in HFpEF (Edelmann *et al*, 2013; Pitt *et al*, 2014). A notable caveat is that post hoc analysis of the TOPCAT trial of MRA in HFpEF suggested that hospitalization and symptoms may be improved in subgroups of the population (Girerd *et al*, 2016) and that markers of collagen turnover, PICP and PIIINP are reduced (Kosmala *et al*, 2011; Ravassa *et al*, 2018; Xiang *et al*, 2019). Despite this, in the absence of positive prospective RCT data in HFpEF, conclusive evidence for a clinically meaningful cardiac antifibrotic effect of MRA remains elusive.

### Neprilysin inhibitors—the “new” old

The natriuretic peptides (NP), atrial natriuretic peptide and brain natriuretic peptide (BNP) are released by cardiomyocytes in response to stress including mechanical stretch or stimulation by profibrotic factors (Liang & Gardner, 1999; Pikkarainen *et al*, 2003). The effects of NP provide endogenous antifibrotic, vasodilatory and natriuretic effects which counters many of the deleterious effects of the RAAS (Kerkelä *et al*, 2015). Natriuretic peptide receptors A (NPRA), B (NPRB) and C (NPRC) are guanylyl cyclase‐coupled receptors, with NPRA being the most relevant in cardiovascular disease (Kerkelä *et al*, 2015). NP binding increases intracellular cGMP and decreases cAMP and IP_3_ which counters the profibrotic and hypertrophic signalling in fibroblasts and cardiomyocytes (Fujisaki *et al*, 1995). This protective role is highlighted by the increased fibrosis and LV hypertrophy which occurs animals with KO of BNP gene (*Nppb*) or NPRA gene (*Npr1*) in response to AngII infusion or TAC (Tamura *et al*, 2000; Patel *et al*, 2005; Parthasarathy *et al*, 2013).

Exogenous administration of recombinant human BNP has been trialled in patients with acute HF; however, no significant survival or rehospitalization benefit was demonstrated in these trials (O'Connor *et al*, 2011). The half‐life of circulating NPs is under 20 mins as the molecules are readily degraded by the widely expressed membrane‐bound peptidase, neprilysin (Charles *et al*, 1996). Consequently, NPs can be used only as a continuous infusion, unsuitable for use in chronic HF. Inhibitors of the neprilysin peptidase have instead been employed to prolong the half‐life of endogenously produced NPs and have shown promise. Treatment with the combination of angiotensin receptor blockers (ARB) and neprilysin inhibitors in diabetic mice results in reduced interstitial fibrosis and cardiomyocyte hypertrophy (Suematsu *et al*, 2016). Further *in vitro* experiments have shown that, in contrast to angiotensin receptor inhibition which primarily reduces fibroblast proliferation, the NP system more potently inhibits myofibroblasts activation in response to profibrotic stimuli therefore providing a complimentary antifibrotic effects on the CF (Burke *et al*, 2019).

Clinical trials of the ARB—neprilysin inhibitor (ARNI) combination of valsartan and sacubitril, have demonstrated significant efficacy in reducing symptoms, hospitalization and mortality in HFrEF in addition to standard therapy, including RAAS inhibition (McMurray *et al*, 2014). Subgroup analysis showed that an effect on fibrosis may be responsible, in part, for the improvement in outcomes: MMP‐2 and MMP‐9 levels were lower in treated patients compared to controls, and PINP was also reduced in the treatment group (Zile *et al*, 2019). However disappointingly, a recent RCT in HFpEF failed to meet its primary endpoints of reducing hospitalization for HF or death from cardiovascular causes (Solomon *et al*, 2019). Exploratory subgroup analysis of this trial has yielded some interesting results in particular a significant improvement in the primary outcome in women (Solomon *et al*, 2019) which is particularly intriguing given the high burden of HFpEF in women (Vasan *et al*, 2018), and this finding may stimulate further investigation into sex‐specific differences in the development of cardiac fibrosis.

## New directions

Given the issues that have emerged with established fibrosis targets, including the lack of clinical benefit in multiple large clinical trials in HFpEF and the toxicities associated with TGFβ therapy, once the front runner, there is a need to identify alternative approaches and targets for fibrosis. This may include augmenting the effects of currently used treatments, repurposing of drugs which have been proven to be effective in other fibrotic diseases and novel approaches and targets which may provide a much needed alternative antifibrotic treatment.

### Alternative angiotensin inhibitors

Traditional RAAS inhibition has limited effect in treating cardiac fibrosis in HFpEF; hence, the role of ACE‐independent AngII production has been explored as an alternative strategy. Chymases are proteolytic enzymes released predominantly by neutrophils but also by cardiomyocytes and fibroblasts (Urata *et al*, 1990). Chymases activate angiotensinogen via an alternative pathway independent of ACE bypassing the effect of ACE‐I (Prosser *et al*, 2009; Ahmad *et al*, 2016; Froogh *et al*, 2017). Furthermore, there is evidence that this alternative pathway for AngII production can occur intracellularly without the need for the membrane‐bound receptor thereby also evading the action of ARBs (Ferrario *et al*, 2005; Baker & Kumar, 2006). This *intracrine* pathway may be especially important in diabetes (Singh *et al*, 2008) and in hypertension where chymase‐dependent intracellular AngII synthesis is upregulated (Tadevosyan *et al*, 2017).

In animal models, chymase inhibitors reduce TGFβ expression, ECM matrix deposition and improve diastolic parameters in the setting of myocarditis or tachycardia‐mediated fibrosis (Matsumoto *et al*, 2003; Palaniyandi *et al*, 2007; Wei *et al*, 2010) despite having no significant effect on blood pressure in rodents or humans (Kirimura *et al*, 2005; Kanefendt *et al*, 2019). Early clinical trials of oral chymase inhibitors are ongoing, they appear safe in phase I studies (Kanefendt *et al*, 2019), and this could represent a promising future additive therapy to target the angiotensin pathway.

### Small molecule inhibitors of generic fibrosis pathways

Treatment of idiopathic pulmonary fibrosis (IPF) has been transformed by the use of the small molecule inhibitors pirfenidone and nintedanib (King *et al*, 2014; Flaherty *et al*, 2019), and tranilast has been used in the treatment of asthma and keloid scars for over 30 years in Japan (Darakhshan & Pour, 2015). All three drugs also have antifibrotic effects outside the lung in animals (Seniutkin *et al*, 2018; Susutlertpanya *et al*, 2019), and repurposing these drugs for the treatment of cardiac fibrosis is being actively explored.

Pirfenidone and tranilast, through unclear mechanisms (Aimo *et al*, 2020), both reduce the expression and secretion of TGFβ in CF *in vitro* and subsequently reduce myofibroblast transformation in response to profibrotic stimulation (Martin *et al*, 2005; Shi *et al*, 2011). *In vivo* studies in mice with TAC, AngII infusion, diabetes and doxorubicin induced models of cardiomyopathy demonstrated a protective effect of pirfenidone on myocardial collagen accumulation and LV function without the early increased mortality which is seen in the acute phase with *Tgfb1* KOs or TGFβRI inhibitors (Giri *et al*, 2004; Yamazaki *et al*, 2012; Wang *et al*, 2013). Similarly, tranilast reduced the expression of TGFβ in canine models of atrial fibrosis induced by tachypacing and prevented the development of atrial arrhythmias (Nakatani *et al*, 2013).

Pirfenidone and tranilast are already approved for use in humans, and toxicity profiles are well understood. Long‐term pirfenidone treatment requires surveillance for hepatotoxicity; however, it is generally well tolerated and tranilast has limited toxicities (Lancaster *et al*, 2017). The PIROUETTE trial is a clinical study currently ongoing in the UK to investigate the antifibrotic effect of pirfenidone in HFpEF using CMR measures of fibrosis (Lewis *et al*, 2019). Importantly, this trial is selecting patients with HFpEF based on fibrosis burden using ECV on CMR which will provide a stratified population with proven cardiac fibrosis in contrast to the more heterogeneous populations normally component of HFpEF trials (McMurray & O'Connor, 2014).

Nintedanib, a tyrosine kinase inhibitor, has a wide range of targets including the vascular endothelial growth factor receptor, PDGF and fibroblast growth factor receptor (Hilberg *et al*, 2008). Its effects on the fibroblast prevent ECM production and myofibroblast activation (Hostettler *et al*, 2014; Rangarajan *et al*, 2016). In animal studies of pulmonary hypertension, nintedanib reduces right heart fibrosis and remodelling (Rol *et al*, 2019). However, studies specifically in HFpEF have yet to be done.

### Galectin‐3 inhibitors

Gal‐3 is a member of the lectin family of carbohydrate binding molecules which stimulates ECM production and myofibroblast activation when applied to CF (Shen *et al*, 2018). The effects of Gal‐3 increase macrophages infiltration into the myocardium (Sharma *et al*, 2004) and increase oxidative stress secondary to the upregulation of NADPH oxidase 4 (NOX4) (He *et al*, 2017) and downregulation of antioxidant molecules (Ibarrola *et al*, 2018).

Serum levels of Gal‐3 are elevated in HF, and levels are correlated with ventricular dysfunction, arrhythmias and mortality (Ho *et al*, 2012; Wu *et al*, 2015). Multiple profibrotic *in vivo* models increase the expression of Gal‐3 in the heart including TAC, AngII or aldosterone infusion (Calvier *et al*, 2013; Song *et al*, 2015; Frunza *et al*, 2016). The initial work on Gal‐3 by Sharma *et al* (2004) demonstrated that exogenous infusion of Gal‐3 into the pericardial space results in extensive deposition of myocardial collagen and LV dysfunction in rats. Interference with Gal‐3 signalling by transgenic KO or specific Gal‐3 inhibitors blunts the fibrotic response and prevents LV deterioration in rodent models of pressure overload, hypertension and obesity‐related cardiac fibrosis (Yu *et al*, 2013; Calvier *et al*, 2015; Martínez‐Martínez *et al*, 2015).

As yet, there have been no clinical trials investigating the effect of Gal‐3 inhibitors in HF. However, trials in a number of non‐cardiac organ systems are ongoing including pulmonary fibrosis (NCT02257177) non‐alcoholic steatohepatitis (NCT02421094) and psoriasis (NCT02407041).

### Oxidative stress

Oxidative stress is increased in the heart in all forms of HF, and increased ROS is associated with decompensation of HF (Hill & Singal, 1996; Mallat *et al*, 1998), activation of the MAPK pathways (Tanaka *et al*, 2001; Qin *et al*, 2003) and increased interstitial fibrosis (Cheng *et al*, 2003; Lijnen *et al*, 2006). Mice with transgenic knockout of the ROS scavenger, superoxide dismutase, rapidly develop cardiac fibrosis and die within 10 days of life (Li *et al*, 1995). In contrast, overexpression of this enzyme prevents the development of cardiac fibrosis in aged mice (Kwak *et al*, 2015). The main sources of ROS in HF are derived from mitochondria dysfunction, NADPH oxidases (NOX, particularly NOX2 and 4), nitric oxide synthase and xanthine oxidases (Ide *et al*, 2000; Moris *et al*, 2017). Inhibition of the NOX enzymes in particular has emerged as an intriguing antifibrotic target. NOX activity has been found to be upregulated in the explanted hearts of patients with end‐stage HF (Heymes *et al*, 2003; Nediani *et al*, 2007). Multiple profibrotic factors including AngII (Byrne *et al*, 2003; Johar *et al*, 2006), ET‐1 (Duerrschmidt *et al*, 2000) and aldosterone (Johar *et al*, 2006) infusion as well as TAC (Kai *et al*, 2006; Ago *et al*, 2010) upregulate the activity of NOX enzymes resulting in increased ROS production in mice.

NOX2 and NOX4 are the major isoforms responsible for ROS production in the heart and are expressed in cardiomyocytes, endothelial cells and fibroblasts (Lassègue *et al*, 2012; Matsushima *et al*, 2016). Transgenic global KO of *Nox2* reduces cardiac fibrosis in response to AngII or aldosterone infusion compared to wild‐type animals (Byrne *et al*, 2003; Johar *et al*, 2006). However, NOX2 has an important role in the bactericidal effects of phagocytic cells, and hence, NOX2 deficiency results in granulomatous disease in both mice and humans, making it a challenging therapeutic target (O'Neill *et al*, 2015).

*Nox4* KO mice are phenotypically normal displaying only moderately increased body weight and exhibit no notable immune dysfunction (Carnesecchi *et al*, 2011). At a cellular level in CF, NOX4 expression is elevated in response to TGFβ signalling (Cucoranu *et al*, 2005) and knockdown of NOX4 activity using siRNA or small molecule inhibitors reduces fibrosis and myofibroblasts differentiation in response to TGFβ or AngII stimulation (Cucoranu *et al*, 2005; Chan *et al*, 2013; Somanna *et al*, 2016). TAC‐mediated myocardial fibrosis is enhanced in mice by transgenic overexpression of *Nox4* (Kuroda *et al*, 2010), and cardiomyocyte‐specific KO is protective against myocardial fibrosis and left ventricular dysfunction (Kuroda *et al*, 2010; Zhao *et al*, 2015). However, conflicting results have emerged, suggesting that pleiotropic roles of NOX4 in angiogenesis and fatty acid oxidation may be adaptive in the stressed heart and that inhibition of *Nox4* may be detrimental in some contexts (Zhang *et al*, 2010; Nabeebaccus *et al*, 2017; Schnelle *et al*, 2019), and it may demonstrate on‐target toxicities in other systems, such as promoting atherosclerosis (Schürmann *et al*, 2015).

Recent translational trials of the NOX1/4 inhibitor GK137831 in humans were well tolerated in diabetic nephropathy however failed to show a significant reduction in albuminuria at relatively low dose of the drug (Reutens *et al*, 2019; NCT02010242). Further studies in diabetic nephropathy (Reutens *et al*, 2019) and idiopathic pulmonary fibrosis (NCT03865927) are planned, and trials of specific NOX inhibitors in cardiac fibrosis may follow depending on the tolerability and success of these trials.

### SGLT2 inhibitors

SGLT2 inhibitors (SGLT2‐I) are a class of antidiabetic drugs that demonstrated unexpectedly beneficial effects in HF during the EMPA‐Reg trial in diabetes management (Zinman *et al*, 2015). This has subsequently been confirmed to be a class effect in alternative SGLT2 inhibitor trials (Neal *et al*, 2017; McMurray *et al*, 2019), and much interest has since surrounded the use of these drugs in the management of fibrosis and HFpEF, with or without comorbid diabetes mellitus.

SGLT2 is a membrane transporter acting primarily in the proximal convoluted tubule of the kidney to reabsorb glucose and sodium (Kalra, 2014). Mouse studies demonstrated a reduction in cardiac collagen content and improved indices of diastolic function with SGLT2‐I in both diabetic (Habibi *et al*, 2017; Ye *et al*, 2017; Li *et al*, 2019) and non‐diabetic animals (Lee *et al*, 2019; Oh *et al*, 2019).

However, the mechanism responsible for these cardiovascular effects remains elusive particularly as the SGLT2 receptor is not expressed within the heart (Di Franco *et al*, 2017). A recent study in mice demonstrated that NOX4 expression was reduced following SGLT2‐I treatment with an associated reduction in myocardial oxidative stress (Li *et al*, 2019) and improvement in both systolic and diastolic function in the diabetic mouse heart (Osorio *et al*, 2012; Kusaka *et al*, 2016).

There are also a number of potentially beneficial off‐target effects which may play a role in the antifibrotic effects of SGLT2 inhibition. This includes the sodium hydrogen exchanger (Baartscheer *et al*, 2017) and the SGLT1 channels (Zhou *et al*, 2003; Di Franco *et al*, 2017) both of which are expressed within the heart, are involved in myocardial hypertrophy and fibrosis and are targeted by SGLT2‐I. A multitude of beneficial systemic effects are also associated with SGLT2‐Is including reduction in blood pressure, blood sugar and reduced renal RAAS expression in diabetic mice (Georgianos & Agarwal, 2019; Woods *et al*, 2019), and it is likely that the beneficial effects on cardiac health are multifactorial (Chin *et al*, 2019).

Clinical studies in HFpEF have demonstrated an improvement in LV mass and diastolic dysfunction following 3 months of SGLT2‐I treatment (Verma *et al*, 2016). However, ECV measured using CMR was not improved after 6 months of SGLT2 inhibition (Hsu *et al*, 2019). RCTs are currently underway in patients with HFpEF (NCT03619213, NCT03057951), and further mechanistic studies will be vital to improve understanding into the mechanisms involved in these apparent antifibrotic effects.

### Interleukin‐11

IL11 was recently identified as a critical regulator of the TGFβ pathway and cardiac fibrosis: in the absence of IL11 activity, TGFβ cannot exert a profibrotic effect on human cardiac fibroblasts (Schafer *et al*, 2017). Cell culture experiments with human atrial fibroblasts found that stimulation with TGFβ increases the expression of IL11 mRNA by 8.4‐fold on average, making IL11 the most highly upregulated gene downstream of TGFβ/SMAD signalling in CFs. Similar findings have been reported for lung fibroblasts, hepatic stellate cells and coronary artery VSMCs (Lebastchi *et al*, 2011; Schafer *et al*, 2017; Ng *et al*, 2019; Widjaja *et al*, 2019b).

IL11 is a member of the interleukin‐6 (IL6) family of cytokines but has distinct properties from other family members. In the heart, the IL11 receptor (IL11RA1) is highly expressed on fibroblasts (Schafer *et al*, 2017), and following binding of IL11 to its receptor, it then binds gp130 and results in dimerization of a hexameric receptor complex. Canonical gp130 signalling—crucial for IL6 signalling—occurs via the Jak‐STAT pathway and stimulates pro‐inflammatory gene transcription. In contrast, IL11 exerts its main effects in human and mouse fibroblasts at the post‐transcriptional level, via sustained activation of the non‐canonical ERK signalling pathway, with little evidence for a role of STAT although it is mildly phosphorylated (Heinrich *et al*, 2003; Schafer *et al*, 2017). In IL11 stimulated fibroblasts, collagen, ACTA2, periostin and MMP2 are strongly upregulated at the protein level but—surprisingly—there is no detectable change in their respective mRNA expression levels. The mechanism underlying this effect is not yet clear, but there is evidence that IL11 stimulates downstream targets of ERK which activate translation, including 40S ribosomal protein S6 kinase and eukaryotic translation initiation factor 4E (Schafer *et al*, 2017). Glutamyl‐prolyl‐tRNA synthetase, which mediates translation of proline‐rich proteins such as collagen (and IL11 itself), may also play a role in the post‐transcriptional control mechanism (preprint: Wu *et al*, 2019).

In rodents, IL11 is highly expressed in the heart after MI (Obana *et al*, 2010) or pressure overload (Schafer *et al*, 2017). *In vivo,* overexpression of IL11 specifically within fibroblasts produces extensive fibrosis across multiple organs including the heart, lungs and kidney along with a HF phenotype (Schafer *et al*, 2017; Ng *et al*, 2019). In contrast, mice with germline KO of the mouse *Il11ra1* or following treatment with an IL11 neutralizing mAb exhibit resistance to cardiac fibrosis in response to pressure overload or AngII infusion (Schafer *et al*, 2017).

These data contrast with previous work that showed recombinant human IL11 (rhIL11) is protective and antifibrotic in the mouse heart (Obana *et al*, 2010, 2012). However, the use of non‐species‐specific rhIL11 in these earlier studies is of central importance because more recent work (preprint: Widjaja *et al*, 2019a) has shown that rhIL11 unexpectedly functions as an inhibitor of endogenous IL11 in mice and rhIL11 does not activate mouse fibroblast ERK signalling (Schafer *et al*, 2017). In contrast, administration of recombinant *mouse* IL11 is strongly profibrotic in mice *in vivo* and to mouse fibroblasts *in vitro* (Schafer *et al*, 2017). In humans, circulating levels of IL11 are elevated in patients with HF, increase with progressive worsening of HF symptoms and are correlated with cardiovascular events including HF hospitalization, stroke, MI and death (Ye *et al*, 2019).

There is an intriguing side story to IL11, relating to its effect when injected to humans. RhIL11 was developed in the 1990s as a drug (Neumega) for treating chemotherapy‐associated thrombocytopenia as it was opportunistically found to increase platelet counts when injected at high doses, although IL11 has no detectable physiological role for normal platelet production (Nandurkar *et al*, 1997). Notable side effects seen in patients receiving rhIL11 include cardiac arrhythmia including AF, pulmonary congestion, dilutional anaemia and raised brain natriuretic peptide (Smith, 2000; Bhatia *et al*, 2007; Liu *et al*, 2019). In a recent study of leukaemic patients receiving rhIL11 therapy, all patients exhibited increased BNP levels, 80% reached BNP levels consistent with a diagnosis of HF and 16% developed a clinical HF syndrome (Smith, 2000; Bhatia *et al*, 2007; Liu *et al*, 2019).

### Targeting the fibroblast

As myofibroblasts play a central role in the development of fibrosis, the ability to selectively target‐specific populations of fibroblasts is an intriguing potential method to treat fibrotic diseases. The developing oncology treatment, chimeric antigen receptor T‐cell (CAR‐T cell) therapy, uses re‐engineered cytotoxic T cells to target specifically selected surface markers to deplete a defined cell population (June *et al*, 2018). CAR‐T therapy has been used effectively in the clinic to treat B‐cell leukaemias and lymphomas resistant to standard therapy by targeting CD19‐positive cells (Porter *et al*, 2011; Schuster *et al*, 2017; Park *et al*, 2018). In a recent study, Aghajanian *et al* (2019) repurposed this technology to selectively target fibroblasts. Using RNA sequencing data from the tissue of heart transplant donors and recipients, they identified a surface marker minimally expressed in the normal heart or extracardiac tissue but significantly upregulated in CF of humans with HCM and DCM (Tillmanns *et al*, 2015; Nagaraju *et al*, 2019). The marker, fibroblast activation protein (FAP), has previously been shown to be present on activated fibroblasts within malignant tumours (Cortez *et al*, 2014; Kilvaer *et al*, 2015) and is present in activated CF in mice following AngII/phenylephrine infusion. Selective elimination of FAP‐positive cells using this treatment reduced cardiac fibrosis in mice within the 8‐week treatment period and improved cardiac function (Aghajanian *et al*, 2019). Although still at a very early stage of preclinical development, the potential to deplete the activated fibroblast population may be a powerful tool to treat fibrosis within the myocardium and elsewhere.

Another potential method of targeting the CF is to use defined transcription factors to promote fibroblast transdifferentiation towards a cardiomyocyte phenotype, thereby reducing ECM production and potentially augmenting cardiac contraction (Ieda *et al*, 2010). Delivery of these factors using retroviruses or adeno‐associated viral vectors has been trialled successfully in a number of mouse models of HF following MI. Direct injection of the vector into the peri‐infarct area induces the expression of transcription factors GATA4, MEF2C and Tbx5 within fibroblasts and reprograms these cells towards a cardiomyocyte phenotype (Qian *et al*, 2012; Song *et al*, 2012). The result is depletion of the fibroblast population, reduced collagen accumulation and improved cardiac function (Qian *et al*, 2012; Yoo *et al*, 2018). This approach remains very much in its infancy and safety concerns remain regarding the use and specificity of viral vectors as well as the arrhythmogenic potential of generating new cardiomyocytes. However, this work again highlights the diversity of the novel tools being explored to treat cardiac fibrosis.

## Conclusion

Cardiac fibrosis is central to the pathogenesis of HF, particularly HFpEF, and addressing the lack of available treatments for HFpEF is a priority given the rising demographics of obese, diabetic, hypertensive and ageing populations around the world. The cellular and molecular processes which lead to fibrosis are intricate and overlapping. Some of the pathways and treatment strategies discussed in this review have been understood and used for many decades but none specifically target cardiac fibrosis. It is an exciting time in the field of cardiac fibrosis as several emerging targets and approaches show promise and could be developed to treat, and perhaps even reverse cardiac fibrosis, but this can only be assessed through clinical trials. Given redundancies, it is possible that combination therapies that target multiple components of the fibrotic pathway will be more effective than any single therapy but polypharmacy comes with polytoxicity and this is particularly troublesome in elderly patients. It is important to remember also that HFpEF is a multisystem disorder, and therapies that alleviate skeletal muscle, renal and metabolic dysfunction as well as cardiac dysfunction are likely to have greatest clinical efficacy.

Ultimately, fibrosis depends on activation of the fibroblast and its transformation into a matrix‐secreting and pro‐inflammatory myofibroblast. This central pathology is a point of convergence for all upstream stimuli: from mechanical stretch to endocrine or paracrine factors. We end by suggesting that targeting non‐redundant pathways for myofibroblast activation represents the most promising means of treating fibrosis. While the days of attempting to target TGFβ activation, either directly or indirectly, are likely limited due to dose‐limiting, on‐target toxicities—new opportunities are available in the form of cell therapy and novel targets, which should be explored.

## Conflict of interest

MS and BC have no conflicts of interest to disclose. SAC is a co‐inventor on a number of patent applications relating to the role of IL11 in human diseases that include the published patents: WO2017103108, WO2017103108 A2, WO 2018/109174 A2, WO 2018/109170 A2. SAC is also a co‐founder, director and shareholder of Enleofen Bio PTE LTD, a Singapore‐based biotechnology.

## For more information

(i) https://www.escardio.org/Sub-specialty-communities/Heart-Failure-Association-of-the-ESC-(HFA)
(ii) https://www.bsh.org.uk/
(iii) https://www.heart.org/en/health-topics/heart-failure
(iv) https://clinicaltrials.gov

**Pending issues**

(i) Identify non‐redundant mediators of cardiac fibrosis which can be therapeutically targeted with an acceptable safety profile.
(ii) Develop therapies to deplete matrix‐secreting cardiac myofibroblasts that do not adversely affect homeostatic functions of resident cardiac fibroblasts.
(iii) Large animal and first‐in-man studies for preclinical targets including IL11, gal‐3 or NOX inhibition, among others.
(iv) Dissect the interplay of fibrosis and inflammation in the heart to prioritize nodal points of disease pathogenesis and cross‐talk.
(v) Identify cross‐tissue mediators of fibro‐inflammation to enable treatment of the HFpEF, multiorgan syndrome rather than cardiac‐specific pathology.

## References

[emmm201910865-bib-0001] Abhayaratna WP , Marwick TH , Smith WT , Becker NG (2006) Characteristics of left ventricular diastolic dysfunction in the community: an echocardiographic survey. Heart 92: 1259–1264 16488928 10.1136/hrt.2005.080150 PMC1861192

[emmm201910865-bib-0002] Accornero F , van Berlo JH , Correll RN , Elrod JW , Sargent MA , York A , Rabinowitz JE , Leask A , Molkentin JD (2015) Genetic analysis of connective tissue growth factor as an effector of transforming growth factor β signaling and cardiac remodeling. Mol Cell Biol 35: 2154 25870108 10.1128/MCB.00199-15 PMC4438237

[emmm201910865-bib-0003] Acharya A , Baek ST , Huang G , Eskiocak B , Goetsch S , Sung CY , Banfi S , Sauer MF , Olsen GS , Duffield JS *et al* (2012) The bHLH transcription factor Tcf21 is required for lineage‐specific EMT of cardiac fibroblast progenitors. Development 139: 2139–2149 22573622 10.1242/dev.079970 PMC3357908

[emmm201910865-bib-0004] Aghajanian H , Kimura T , Rurik JG , Hancock AS , Leibowitz MS , Li L , Scholler J , Monslow J , Lo A , Han W *et al* (2019) Targeting cardiac fibrosis with engineered T cells. Nature 573: 430–433 31511695 10.1038/s41586-019-1546-z PMC6752964

[emmm201910865-bib-0005] Ago T , Kuroda J , Pain J , Fu C , Li H , Sadoshima J (2010) Upregulation of Nox4 by hypertrophic stimuli promotes apoptosis and mitochondrial dysfunction in cardiac myocytes. Circ Res 106: 1253–1264 20185797 10.1161/CIRCRESAHA.109.213116 PMC2855780

[emmm201910865-bib-0006] Ahmad S , Varagic J , VonCannon JL , Groban L , Collawn JF , Dell'Italia LJ , Ferrario CM (2016) Primacy of cardiac chymase over angiotensin converting enzyme as an angiotensin‐(1‐12) metabolizing enzyme. Biochem Biophys Res Commun 478: 559–564 27465904 10.1016/j.bbrc.2016.07.100 PMC5207032

[emmm201910865-bib-0007] Aimo A , Cerbai E , Bartolucci G , Adamo L , Barison A , Lo Surdo G , Biagini S , Passino C , Emdin M (2020) Pirfenidone is a cardioprotective drug: mechanisms of action and preclinical evidence. Pharmacol Res 155: 104694 32061664 10.1016/j.phrs.2020.104694

[emmm201910865-bib-0008] Alex L , Russo I , Holoborodko V , Frangogiannis NG (2018) Characterization of a mouse model of obesity‐related fibrotic cardiomyopathy that recapitulates features of human heart failure with preserved ejection fraction. Am J Physiol Heart Circ Physiol 315: H934–H949 30004258 10.1152/ajpheart.00238.2018 PMC6230908

[emmm201910865-bib-0009] Almendral JL , Shick V , Rosendorff C , Atlas SA (2010) Association between transforming growth factor‐beta(1) and left ventricular mass and diameter in hypertensive patients. J Am Soc Hypertens 4: 135–141 20470998 10.1016/j.jash.2010.02.007

[emmm201910865-bib-0010] van Amerongen MJ , Bou‐Gharios G , Popa ER , van Ark J , Petersen AH , van Dam GM , van Luyn MJA , Harmsen MC (2008) Bone marrow‐derived myofibroblasts contribute functionally to scar formation after myocardial infarction. J. Pathol. 214: 377–386 18095257 10.1002/path.2281

[emmm201910865-bib-0011] Anderton MJ , Mellor HR , Bell A , Sadler C , Pass M , Powell S , Steele SJ , Roberts RR , Heier A (2011) Induction of heart valve lesions by small‐molecule ALK5 inhibitors. Toxicol Pathol 39: 916–924 21859884 10.1177/0192623311416259

[emmm201910865-bib-0012] Asgari M , Latifi N , Heris HK , Vali H , Mongeau L (2017) *In vitro* fibrillogenesis of tropocollagen type III in collagen type I affects its relative fibrillar topology and mechanics. Sci Rep 7: 1392 28469139 10.1038/s41598-017-01476-y PMC5431193

[emmm201910865-bib-0013] Baartscheer A , Schumacher CA , Wüst RCI , Fiolet JWT , Stienen GJM , Coronel R , Zuurbier CJ (2017) Empagliflozin decreases myocardial cytoplasmic Na(+) through inhibition of the cardiac Na(+)/H(+) exchanger in rats and rabbits. Diabetologia 60: 568–573 27752710 10.1007/s00125-016-4134-x PMC6518059

[emmm201910865-bib-0014] Badenhorst D , Maseko M , Tsotetsi OJ , Naidoo A , Brooksbank R , Norton GR , Woodiwiss AJ (2003) Cross‐linking influences the impact of quantitative changes in myocardial collagen on cardiac stiffness and remodelling in hypertension in rats. Cardiovasc Res 57: 632–641 12618225 10.1016/s0008-6363(02)00733-2

[emmm201910865-bib-0015] Baker KM , Kumar R (2006) Intracellular angiotensin II induces cell proliferation independent of AT1 receptor. Am J Physiol Cell Physiol 291: C995–C1001 16774988 10.1152/ajpcell.00238.2006

[emmm201910865-bib-0016] Ballal K , Wilson CR , Harmancey R , Taegtmeyer H (2010) Obesogenic high fat western diet induces oxidative stress and apoptosis in rat heart. Mol Cell Biochem 344: 221–230 20676734 10.1007/s11010-010-0546-y PMC3656656

[emmm201910865-bib-0017] Banerjee I , Fuseler JW , Price RL , Borg TK , Baudino TA (2007) Determination of cell types and numbers during cardiac development in the neonatal and adult rat and mouse. Am J Physiol Heart Circ Physiol 293: H1883–H1891 17604329 10.1152/ajpheart.00514.2007

[emmm201910865-bib-0018] Barcellos‐Hoff MH , Dix TA (1996) Redox‐mediated activation of latent transforming growth factor‐beta 1. Mol Endocrinol 10: 1077–1083 8885242 10.1210/mend.10.9.8885242

[emmm201910865-bib-0019] Bekfani T , Pellicori P , Morris DA , Ebner N , Valentova M , Steinbeck L , Wachter R , Elsner S , Sliziuk V , Schefold JC *et al* (2016) Sarcopenia in patients with heart failure with preserved ejection fraction: Impact on muscle strength, exercise capacity and quality of life. Int J Cardiol 222: 41–46 27454614 10.1016/j.ijcard.2016.07.135

[emmm201910865-bib-0020] Bhatia M , Davenport V , Cairo MS (2007) The role of interleukin‐11 to prevent chemotherapy‐induced thrombocytopenia in patients with solid tumors, lymphoma, acute myeloid leukemia and bone marrow failure syndromes. Leuk Lymphoma 48: 9–15 17325843 10.1080/10428190600909115

[emmm201910865-bib-0021] Bishu K , Hamdani N , Mohammed SF , Kruger M , Ohtani T , Ogut O , Brozovich FV , Burnett JC Jr , Linke WA , Redfield MM (2011) Sildenafil and B‐type natriuretic peptide acutely phosphorylate titin and improve diastolic distensibility *in vivo* . Circulation 124: 2882–2891 22144574 10.1161/CIRCULATIONAHA.111.048520 PMC3412357

[emmm201910865-bib-0022] Boudina S , Sena S , Theobald H , Sheng X , Wright JJ , Hu XX , Aziz S , Johnson JI , Bugger H , Zaha VG *et al* (2007) Mitochondrial energetics in the heart in obesity‐related diabetes: direct evidence for increased uncoupled respiration and activation of uncoupling proteins. Diabetes 56: 2457–2466 17623815 10.2337/db07-0481

[emmm201910865-bib-0023] Braunwald E (1988) Heart disease: a text book of cardiovascular medicine, 3rd edn Philidelphia, PA: Elsevier/Saunders

[emmm201910865-bib-0024] Brennan FR , Cavagnaro J , McKeever K , Ryan PC , Schutten MM , Vahle J , Weinbauer GF , Marrer‐Berger E , Black LE (2018) Safety testing of monoclonal antibodies in non‐human primates: case studies highlighting their impact on human risk assessment. MAbs 10: 1–17 28991509 10.1080/19420862.2017.1389364 PMC5800363

[emmm201910865-bib-0025] Brilla CG , Zhou G , Matsubara L , Weber KT (1994) Collagen metabolism in cultured adult rat cardiac fibroblasts: response to angiotensin II and aldosterone. J Mol Cell Cardiol 26: 809–820 7966349 10.1006/jmcc.1994.1098

[emmm201910865-bib-0026] Brilla CG , Funck RC , Rupp H (2000) Lisinopril‐mediated regression of myocardial fibrosis in patients with hypertensive heart disease. Circulation 102: 1388–1393 10993857 10.1161/01.cir.102.12.1388

[emmm201910865-bib-0027] Brooks WW , Conrad CH (2000) Myocardial fibrosis in transforming growth factor beta(1)heterozygous mice. J Mol Cell Cardiol 32: 187–195 10722796 10.1006/jmcc.1999.1065

[emmm201910865-bib-0028] Brown NJ (2013) Contribution of aldosterone to cardiovascular and renal inflammation and fibrosis. Nat Rev Nephrol 9: 459–469 23774812 10.1038/nrneph.2013.110 PMC3922409

[emmm201910865-bib-0029] Bruder O , Wagner A , Jensen CJ , Schneider S , Ong P , Kispert E‐M , Nassenstein K , Schlosser T , Sabin GV , Sechtem U *et al* (2010) Myocardial scar visualized by cardiovascular magnetic resonance imaging predicts major adverse events in patients with hypertrophic cardiomyopathy. J Am Coll Cardiol 56: 875–887 20667520 10.1016/j.jacc.2010.05.007

[emmm201910865-bib-0030] Bui AL , Horwich TB , Fonarow GC (2011) Epidemiology and risk profile of heart failure. Nat Rev Cardiol 8: 30–41 21060326 10.1038/nrcardio.2010.165 PMC3033496

[emmm201910865-bib-0031] Burke RM , Lighthouse JK , Mickelsen DM , Small EM (2019) Sacubitril/valsartan decreases cardiac fibrosis in left ventricle pressure overload by restoring PKG signaling in cardiac fibroblasts. Circ Heart Fail 12: e005565 30998392 10.1161/CIRCHEARTFAILURE.118.005565 PMC6530564

[emmm201910865-bib-0032] Byrne JA , Grieve DJ , Bendall JK , Li J‐M , Gove C , Lambeth JD , Cave AC , Shah AM (2003) Contrasting roles of NADPH oxidase isoforms in pressure‐overload versus angiotensin II‐induced cardiac hypertrophy. Circ Res 93: 802–805 14551238 10.1161/01.RES.0000099504.30207.F5

[emmm201910865-bib-0033] Calderone A , Thaik CM , Takahashi N , Chang DL , Colucci WS (1998) Nitric oxide, atrial natriuretic peptide, and cyclic GMP inhibit the growth‐promoting effects of norepinephrine in cardiac myocytes and fibroblasts. J Clin Invest 101: 812–818 9466976 10.1172/JCI119883 PMC508629

[emmm201910865-bib-0034] Calvier L , Miana M , Reboul P , Cachofeiro V , Martinez‐Martinez E , de Boer RA , Poirier F , Lacolley P , Zannad F , Rossignol P *et al* (2013) Galectin‐3 mediates aldosterone‐induced vascular fibrosis. Arterioscler Thromb Vasc Biol 33: 67–75 23117656 10.1161/ATVBAHA.112.300569

[emmm201910865-bib-0035] Calvier L , Martinez‐Martinez E , Miana M , Cachofeiro V , Rousseau E , Sádaba JR , Zannad F , Rossignol P , López‐Andrés N (2015) The impact of Galectin‐3 inhibition on aldosterone‐induced cardiac and renal injuries. JACC Heart Fail 3: 59–67 25458174 10.1016/j.jchf.2014.08.002

[emmm201910865-bib-0036] Cannavo A , Liccardo D , Eguchi A , Elliott KJ , Traynham CJ , Ibetti J , Eguchi S , Leosco D , Ferrara N , Rengo G *et al* (2016) Myocardial pathology induced by aldosterone is dependent on non‐canonical activities of G protein‐coupled receptor kinases. Nat Commun 7: 10877 26932512 10.1038/ncomms10877 PMC4778065

[emmm201910865-bib-0037] Carnesecchi S , Deffert C , Donati Y , Basset O , Hinz B , Preynat‐Seauve O , Guichard C , Arbiser JL , Banfi B , Pache J‐C *et al* (2011) A key role for NOX4 in epithelial cell death during development of lung fibrosis. Antioxid Redox Signal 15: 607–619 21391892 10.1089/ars.2010.3829 PMC3163392

[emmm201910865-bib-0038] Chan EC , Peshavariya HM , Liu G‐S , Jiang F , Lim S‐Y , Dusting GJ (2013) Nox4 modulates collagen production stimulated by transforming growth factor β1 *in vivo* and *in vitro* . Biochem Biophys Res Commun 430: 918–925 23261430 10.1016/j.bbrc.2012.11.138

[emmm201910865-bib-0039] Charles CJ , Espiner EA , Nicholls MG , Richards AM , Yandle TG , Protter A , Kosoglou T (1996) Clearance receptors and endopeptidase 24.11: equal role in natriuretic peptide metabolism in conscious sheep. Am J Physiol 271: R373–R380 8770137 10.1152/ajpregu.1996.271.2.R373

[emmm201910865-bib-0040] Chen Y , Shi‐Wen X , van Beek J , Kennedy L , McLeod M , Renzoni EA , Bou‐Gharios G , Wilcox‐Adelman S , Goetinck PF , Eastwood M *et al* (2005) Matrix contraction by dermal fibroblasts requires transforming growth factor‐beta/activin‐linked kinase 5, heparan sulfate‐containing proteoglycans, and MEK/ERK: insights into pathological scarring in chronic fibrotic disease. Am J Pathol 167: 1699–1711 16314481 10.1016/s0002-9440(10)61252-7 PMC1613194

[emmm201910865-bib-0041] Cheng T‐H , Cheng P‐Y , Shih N‐L , Chen I‐B , Wang DL , Chen J‐J (2003) Involvement of reactive oxygen species in angiotensin II‐induced endothelin‐1 gene expression in rat cardiac fibroblasts. J Am Coll Cardiol 42: 1845–1854 14642698 10.1016/j.jacc.2003.06.010

[emmm201910865-bib-0042] Chin KL , Ofori‐Asenso R , Hopper I , von Lueder TG , Reid CM , Zoungas S , Wang BH , Liew D (2019) Potential mechanisms underlying the cardiovascular benefits of sodium glucose cotransporter 2 inhibitors: a systematic review of data from preclinical studies. Cardiovasc Res 115: 266–276 30475996 10.1093/cvr/cvy295

[emmm201910865-bib-0043] Cho JH , Zhang R , Aynaszyan S , Holm K , Goldhaber JI , Marbán E , Cingolani E (2018) Ventricular arrhythmias underlie sudden death in rats with heart failure and preserved ejection fraction. Circ Arrhythm Electrophysiol 11: e006452 30030266 10.1161/CIRCEP.118.006452 PMC6091890

[emmm201910865-bib-0044] Cleland JG , Tendera M , Adamus J , Freemantle N , Polonski L , Taylor J (2006) The perindopril in elderly people with chronic heart failure (PEP‐CHF) study. Eur Heart J 27: 2338–2345 16963472 10.1093/eurheartj/ehl250

[emmm201910865-bib-0045] Cohen JB , Schrauben SJ , Zhao L , Basso MD , Cvijic ME , Li Z , Yarde M , Wang Z , Bhattacharya PT , Chirinos DA *et al* (2020) Clinical phenogroups in heart failure with preserved ejection fraction: detailed phenotypes, prognosis, and response to spironolactone. JACC Heart Fail 8: 172–184 31926856 10.1016/j.jchf.2019.09.009 PMC7058514

[emmm201910865-bib-0046] Collier P , Watson CJ , van Es MH , Phelan D , McGorrian C , Tolan M , Ledwidge MT , McDonald KM , Baugh JA (2012) Getting to the heart of cardiac remodeling; how collagen subtypes may contribute to phenotype. J Mol Cell Cardiol 52: 148–153 22008391 10.1016/j.yjmcc.2011.10.002

[emmm201910865-bib-0047] Conrad N , Judge A , Tran J , Mohseni H , Hedgecott D , Crespillo AP , Allison M , Hemingway H , Cleland JG , McMurray JJV *et al* (2018) Temporal trends and patterns in heart failure incidence: a population‐based study of 4 million individuals. Lancet 391: 572–580 29174292 10.1016/S0140-6736(17)32520-5 PMC5814791

[emmm201910865-bib-0048] Cortez E , Roswall P , Pietras K (2014) Functional subsets of mesenchymal cell types in the tumor microenvironment. Semin Cancer Biol 25: 3–9 24412106 10.1016/j.semcancer.2013.12.010

[emmm201910865-bib-0049] Cucoranu I , Clempus R , Dikalova A , Phelann PJ Ariyan S , Dikalov S , Sorescu D (2005) NAD(P)H oxidase 4 mediates transforming growth factor‐β1–induced differentiation of cardiac fibroblasts into myofibroblasts. Circ Res 97: 900–907 16179589 10.1161/01.RES.0000187457.24338.3D

[emmm201910865-bib-0050] Dai Z , Aoki T , Fukumoto Y , Shimokawa H (2012) Coronary perivascular fibrosis is associated with impairment of coronary blood flow in patients with non‐ischemic heart failure. J Cardiol 60: 416–421 22867802 10.1016/j.jjcc.2012.06.009

[emmm201910865-bib-0051] Darakhshan S , Pour AB (2015) Tranilast: a review of its therapeutic applications. Pharmacol Res 91: 15–28 25447595 10.1016/j.phrs.2014.10.009

[emmm201910865-bib-0052] Dennler S , Itoh S , Vivien D , ten Dijke P , Huet S , Gauthier JM (1998) Direct binding of Smad3 and Smad4 to critical TGF beta‐inducible elements in the promoter of human plasminogen activator inhibitor‐type 1 gene. EMBO J 17: 3091–3100 9606191 10.1093/emboj/17.11.3091 PMC1170648

[emmm201910865-bib-0053] Derangeon M , Montnach J , Cerpa CO , Jagu B , Patin J , Toumaniantz G , Girardeau A , Huang CLH , Colledge WH , Grace AA *et al* (2017) Transforming growth factor β receptor inhibition prevents ventricular fibrosis in a mouse model of progressive cardiac conduction disease. Cardiovasc Res 113: 464–474 28339646 10.1093/cvr/cvx026

[emmm201910865-bib-0054] Derynck R , Zhang YE (2003) Smad‐dependent and Smad‐independent pathways in TGF‐β family signalling. Nature 425: 577–584 14534577 10.1038/nature02006

[emmm201910865-bib-0055] Di Carlo SE , Peduto L (2018) The perivascular origin of pathological fibroblasts. J Clin Invest 128: 54–63 29293094 10.1172/JCI93558 PMC5749494

[emmm201910865-bib-0056] Di Franco A , Cantini G , Tani A , Coppini R , Zecchi‐Orlandini S , Raimondi L , Luconi M , Mannucci E (2017) Sodium‐dependent glucose transporters (SGLT) in human ischemic heart: a new potential pharmacological target. Int J Cardiol 243: 86–90 28526540 10.1016/j.ijcard.2017.05.032

[emmm201910865-bib-0057] Diao K‐Y , Yang Z‐G , Xu H‐Y , Liu X , Zhang Q , Shi K , Jiang L , Xie L‐J , Wen L‐Y , Guo Y‐K (2016) Histologic validation of myocardial fibrosis measured by T1 mapping: a systematic review and meta‐analysis. J Cardiovasc Magn Reson 18: 92 27955698 10.1186/s12968-016-0313-7 PMC5154013

[emmm201910865-bib-0058] Díez J , Laviades C , Mayor G , Gil MJ , Monreal I (1995) Increased serum concentrations of procollagen peptides in essential hypertension. Relation to cardiac alterations. Circulation 91: 1450–1456 7867186 10.1161/01.cir.91.5.1450

[emmm201910865-bib-0059] Díez J , Querejeta R , López B , González A , Larman M , Martínez Ubago JL (2002) Losartan‐dependent regression of myocardial fibrosis is associated with reduction of left ventricular chamber stiffness in hypertensive patients. Circulation 105: 2512–2517 12034658 10.1161/01.cir.0000017264.66561.3d

[emmm201910865-bib-0060] Díez J (2004) Profibrotic effects of angiotensin II in the heart. Hypertension 43: 1164–1165 15117918 10.1161/01.HYP.0000128620.57061.67

[emmm201910865-bib-0061] Divakaran V , Adrogue J , Ishiyama M , Entman ML , Haudek S , Sivasubramanian N , Mann DL (2009) Adaptive and maladptive effects of SMAD3 signaling in the adult heart after hemodynamic pressure overloading. Circ Heart Fail 2: 633–642 19919989 10.1161/CIRCHEARTFAILURE.108.823070 PMC3064555

[emmm201910865-bib-0062] Dolivo DM , Larson SA , Dominko T (2019) Crosstalk between mitogen‐activated protein kinase inhibitors and transforming growth factor‐β signaling results in variable activation of human dermal fibroblasts. Int J Mol Med 43: 325–335 30365043 10.3892/ijmm.2018.3949 PMC6257852

[emmm201910865-bib-0063] Duca F , Kammerlander Andreas A , Zotter‐Tufaro C , Aschauer S , Schwaiger Marianne L , Marzluf Beatrice A , Bonderman D , Mascherbauer J (2016) Interstitial fibrosis, functional status, and outcomes in heart failure with preserved ejection fraction. Circ Cardiovasc Imaging 9: e005277 27974408 10.1161/CIRCIMAGING.116.005277

[emmm201910865-bib-0064] Duerrschmidt N , Wippich N , Goettsch W , Broemme HJ , Morawietz H (2000) Endothelin‐1 induces NAD(P)H oxidase in human endothelial cells. Biochem Biophys Res Commun 269: 713–717 10720482 10.1006/bbrc.2000.2354

[emmm201910865-bib-0065] Dunlay SM , Roger VL , Redfield MM (2017) Epidemiology of heart failure with preserved ejection fraction. Nat Rev Cardiol 14: 591–602 28492288 10.1038/nrcardio.2017.65

[emmm201910865-bib-0066] Echegaray K , Andreu I , Lazkano A , Villanueva I , Saenz A , Elizalde MR , Echeverria T , Lopez B , Garro A , Gonzalez A *et al* (2017) Role of myocardial collagen in severe aortic stenosis with preserved ejection fraction and symptoms of heart failure. Rev Esp Cardiol 70: 832–840 28215921 10.1016/j.rec.2016.12.038

[emmm201910865-bib-0067] Edelmann F , Wachter R , Schmidt AG , Kraigher‐Krainer E , Colantonio C , Kamke W , Duvinage A , Stahrenberg R , Durstewitz K , Löffler M *et al* (2013) Effect of spironolactone on diastolic function and exercise capacity in patients with heart failure with preserved ejection fraction: the aldo‐DHF randomized controlled trial. JAMA 309: 781–791 23443441 10.1001/jama.2013.905

[emmm201910865-bib-0068] Engebretsen KVT , Skårdal K , Bjørnstad S , Marstein HS , Skrbic B , Sjaastad I , Christensen G , Bjørnstad JL , Tønnessen T (2014) Attenuated development of cardiac fibrosis in left ventricular pressure overload by SM16, an orally active inhibitor of ALK5. J Mol Cell Cardiol 76: 148–157 25169971 10.1016/j.yjmcc.2014.08.008

[emmm201910865-bib-0069] Engel ME , McDonnell MA , Law BK , Moses HL (1999) Interdependent SMAD and JNK signaling in transforming growth factor‐beta‐mediated transcription. J Biol Chem 274: 37413–37420 10601313 10.1074/jbc.274.52.37413

[emmm201910865-bib-0070] Eschalier R , Rossignol P , Kearney‐Schwartz A , Adamopoulos C , Karatzidou K , Fay R , Mandry D , Marie P‐Y , Zannad F (2014) Features of cardiac remodeling, associated with blood pressure and fibrosis biomarkers, are frequent in subjects with abdominal obesity. Hypertension 63: 740–746 24446063 10.1161/HYPERTENSIONAHA.113.02419

[emmm201910865-bib-0071] Esposito K , Nappo F , Marfella R , Giugliano G , Giugliano F , Ciotola M , Quagliaro L , Ceriello A , Giugliano D (2002) Inflammatory cytokine concentrations are acutely increased by hyperglycemia in humans. Circulation 106: 2067–2072 12379575 10.1161/01.cir.0000034509.14906.ae

[emmm201910865-bib-0072] Everett RJ , Treibel TA , Fukui M , Lee H , Rigolli M , Singh A , Bijsterveld P , Tastet L , Musa TA , Dobson L *et al* (2020) Extracellular myocardial volume in patients with aortic stenosis. J Am Coll Cardiol 75: 304–316 31976869 10.1016/j.jacc.2019.11.032 PMC6985897

[emmm201910865-bib-0073] Ferrario CM , Jessup J , Chappell MC , Averill DB , Brosnihan KB , Tallant EA , Diz DI , Gallagher PE (2005) Effect of angiotensin‐converting enzyme inhibition and angiotensin II receptor blockers on cardiac angiotensin‐converting enzyme 2. Circulation 111: 2605–2610 15897343 10.1161/CIRCULATIONAHA.104.510461

[emmm201910865-bib-0074] Flaherty KR , Wells AU , Cottin V , Devaraj A , Walsh SLF , Inoue Y , Richeldi L , Kolb M , Tetzlaff K , Stowasser S *et al* (2019) Nintedanib in progressive fibrosing interstitial lung diseases. N Engl J Med 381: 1718–1727 31566307 10.1056/NEJMoa1908681

[emmm201910865-bib-0075] Frangogiannis NG (2019) Can myocardial fibrosis be reversed? J Am Coll Cardiol 73: 2283–2285 31072571 10.1016/j.jacc.2018.10.094

[emmm201910865-bib-0076] Frantz S , Hu K , Adamek A , Wolf J , Sallam A , Maier SK , Lonning S , Ling H , Ertl G , Bauersachs J (2008) Transforming growth factor beta inhibition increases mortality and left ventricular dilatation after myocardial infarction. Basic Res Cardiol 103: 485–492 18651091 10.1007/s00395-008-0739-7

[emmm201910865-bib-0077] From AM , Maleszewski JJ , Rihal CS (2011) Current status of endomyocardial biopsy. Mayo Clin Proc 86: 1095–1102 22033254 10.4065/mcp.2011.0296 PMC3203000

[emmm201910865-bib-0078] Froogh G , Pinto JT , Le Y , Kandhi S , Aleligne Y , Huang A , Sun D (2017) Chymase‐dependent production of angiotensin II: an old enzyme in old hearts. Am J Physiol Heart Circ Physiol 312: H223–H231 27815252 10.1152/ajpheart.00534.2016 PMC5336578

[emmm201910865-bib-0079] Frunza O , Russo I , Saxena A , Shinde AV , Humeres C , Hanif W , Rai V , Su Y , Frangogiannis NG (2016) Myocardial Galectin‐3 expression is associated with remodeling of the pressure‐overloaded heart and may delay the hypertrophic response without affecting survival, dysfunction, and cardiac fibrosis. Am J Pathol 186: 1114–1127 26948424 10.1016/j.ajpath.2015.12.017 PMC4861760

[emmm201910865-bib-0080] Fujisaki H , Ito H , Hirata Y , Tanaka M , Hata M , Lin M , Adachi S , Akimoto H , Marumo F , Hiroe M (1995) Natriuretic peptides inhibit angiotensin II‐induced proliferation of rat cardiac fibroblasts by blocking endothelin‐1 gene expression. J Clin Invest 96: 1059–1065 7635942 10.1172/JCI118092 PMC185295

[emmm201910865-bib-0081] Fukuda N , Wu Y , Nair P , Granzier HL (2005) Phosphorylation of titin modulates passive stiffness of cardiac muscle in a titin isoform‐dependent manner. J Gen Physiol 125: 257–271 15738048 10.1085/jgp.200409177 PMC2234012

[emmm201910865-bib-0082] Funaba M , Zimmerman CM , Mathews LS (2002) Modulation of Smad2‐mediated signaling by extracellular signal‐regulated kinase. J Biol Chem 277: 41361–41368 12193595 10.1074/jbc.M204597200

[emmm201910865-bib-0083] Gao X , Wu G , Gu X , Fu L , Mei C (2013) Kruppel‐like factor 15 modulates renal interstitial fibrosis by ERK/MAPK and JNK/MAPK pathways regulation. Kidney Blood Press Res 37: 631–640 24356553 10.1159/000355743

[emmm201910865-bib-0084] Georgianos PI , Agarwal R (2019) Ambulatory blood pressure reduction with SGLT‐2 inhibitors: dose‐response meta‐analysis and comparative evaluation with low‐dose hydrochlorothiazide. Diabetes Care 42: 693–700 30894383 10.2337/dc18-2207 PMC6429633

[emmm201910865-bib-0085] Girerd N , Ferreira JP , Rossignol P , Zannad F (2016) A tentative interpretation of the TOPCAT trial based on randomized evidence from the brain natriuretic peptide stratum analysis. Eur J Heart Fail 18: 1411–1414 27612005 10.1002/ejhf.621

[emmm201910865-bib-0086] Giri SN , Al‐Bayati MA , Du X , Schelegle E , Mohr FC , Margolin SB (2004) Amelioration of doxorubicin‐induced cardiac and renal toxicity by pirfenidone in rats. Cancer Chemother Pharmacol 53: 141–150 14564477 10.1007/s00280-003-0703-z

[emmm201910865-bib-0087] González A , Schelbert EB , Díez J , Butler J (2018) Myocardial interstitial fibrosis in heart failure: biological and translational perspectives. J Am Coll Cardiol 71: 1696–1706 29650126 10.1016/j.jacc.2018.02.021

[emmm201910865-bib-0088] Gray MO , Long CS , Kalinyak JE , Li HT , Karliner JS (1998) Angiotensin II stimulates cardiac myocyte hypertrophy via paracrine release of TGF‐beta 1 and endothelin‐1 from fibroblasts. Cardiovasc Res 40: 352–363 9893729 10.1016/s0008-6363(98)00121-7

[emmm201910865-bib-0089] Gu X , Fang T , Kang P , Hu J , Yu Y , Li Z , Cheng X , Gao Q (2017) Effect of ALDH2 on high glucose‐induced cardiac fibroblast oxidative stress, apoptosis, and fibrosis. Oxid Med Cell Longev 2017: 9257967 29129988 10.1155/2017/9257967 PMC5654254

[emmm201910865-bib-0090] Habibi J , Aroor AR , Sowers JR , Jia G , Hayden MR , Garro M , Barron B , Mayoux E , Rector RS , Whaley‐Connell A *et al* (2017) Sodium glucose transporter 2 (SGLT2) inhibition with empagliflozin improves cardiac diastolic function in a female rodent model of diabetes. Cardiovasc Diabetol 16: 9 28086951 10.1186/s12933-016-0489-z PMC5237274

[emmm201910865-bib-0091] Ham O , Jin W , Lei L , Huang HH , Tsuji K , Huang M , Roh J , Rosenzweig A , Lu HAJ (2018) Pathological cardiac remodeling occurs early in CKD mice from unilateral urinary obstruction, and is attenuated by Enalapril. Sci Rep 8: 16087 30382174 10.1038/s41598-018-34216-x PMC6208335

[emmm201910865-bib-0092] Hara M , Ono K , Hwang M‐W , Iwasaki A , Okada M , Nakatani K , Sasayama S , Matsumori A (2002) Evidence for a role of mast cells in the evolution to congestive heart failure. J Exp Med 195: 375–381 11828013 10.1084/jem.20002036 PMC2193589

[emmm201910865-bib-0093] Hay I , Rich J , Ferber P , Burkhoff D , Maurer MS (2005) Role of impaired myocardial relaxation in the production of elevated left ventricular filling pressure. Am J Physiol Heart Circ Physiol 288: H1203–H1208 15498827 10.1152/ajpheart.00681.2004

[emmm201910865-bib-0094] Hayashi H , Kobara M , Abe M , Tanaka N , Gouda E , Toba H , Yamada H , Tatsumi T , Nakata T , Matsubara H (2008) Aldosterone nongenomically produces NADPH oxidase‐dependent reactive oxygen species and induces myocyte apoptosis. Hypertens Res 31: 363–375 18360057 10.1291/hypres.31.363

[emmm201910865-bib-0095] He J , Li X , Luo H , Li T , Zhao L , Qi Q , Liu Y , Yu Z (2017) Galectin‐3 mediates the pulmonary arterial hypertension‐induced right ventricular remodeling through interacting with NADPH oxidase 4. J Am Soc Hypertens 11: 275–289.e2 28431936 10.1016/j.jash.2017.03.008

[emmm201910865-bib-0096] Hein S , Arnon E , Kostin S , Schönburg M , Elsässer A , Polyakova V , Bauer EP , Klövekorn W‐P , Schaper J (2003) Progression from compensated hypertrophy to failure in the pressure‐overloaded human heart: structural deterioration and compensatory mechanisms. Circulation 107: 984–991 12600911 10.1161/01.cir.0000051865.66123.b7

[emmm201910865-bib-0097] Heinrich PC , Behrmann I , Haan S , Hermanns HM , Müller‐Newen G , Schaper F (2003) Principles of interleukin (IL)‐6‐type cytokine signalling and its regulation. Biochem J 374: 1–20 12773095 10.1042/BJ20030407 PMC1223585

[emmm201910865-bib-0098] Heymes C , Bendall JK , Ratajczak P , Cave AC , Samuel J‐L , Hasenfuss G , Shah AM (2003) Increased myocardial NADPH oxidase activity in human heart failure. J Am Coll Cardiol 41: 2164–2171 12821241 10.1016/s0735-1097(03)00471-6

[emmm201910865-bib-0099] Hilberg F , Roth GJ , Krssak M , Kautschitsch S , Sommergruber W , Tontsch‐Grunt U , Garin‐Chesa P , Bader G , Zoephel A , Quant J *et al* (2008) BIBF 1120: triple angiokinase inhibitor with sustained receptor blockade and good antitumor efficacy. Cancer Res 68: 4774–4782 18559524 10.1158/0008-5472.CAN-07-6307

[emmm201910865-bib-0100] Hill MF , Singal PK (1996) Antioxidant and oxidative stress changes during heart failure subsequent to myocardial infarction in rats. Am J Pathol 148: 291–300 8546218 PMC1861605

[emmm201910865-bib-0101] Hinglais N , Heudes D , Nicoletti A , Mandet C , Laurent M , Bariéty J , Michel JB (1994) Colocalization of myocardial fibrosis and inflammatory cells in rats. Lab Invest 70: 286–294 8139269

[emmm201910865-bib-0102] Ho JE , Liu C , Lyass A , Courchesne P , Pencina MJ , Vasan RS , Larson MG , Levy D (2012) Galectin‐3, a marker of cardiac fibrosis, predicts incident heart failure in the community. J Am Coll Cardiol 60: 1249–1256 22939561 10.1016/j.jacc.2012.04.053 PMC3512095

[emmm201910865-bib-0103] Hostettler KE , Zhong J , Papakonstantinou E , Karakiulakis G , Tamm M , Seidel P , Sun Q , Mandal J , Lardinois D , Lambers C *et al* (2014) Anti‐fibrotic effects of nintedanib in lung fibroblasts derived from patients with idiopathic pulmonary fibrosis. Respir Res 15: 157 25496490 10.1186/s12931-014-0157-3 PMC4273482

[emmm201910865-bib-0104] Hsu JC , Wang CY , Su MM , Lin LY , Yang WS (2019) Effect of empagliflozin on cardiac function, adiposity, and diffuse fibrosis in patients with type 2 diabetes mellitus. Sci Rep 9: 15348 31653956 10.1038/s41598-019-51949-5 PMC6814842

[emmm201910865-bib-0105] Hudon‐David F , Bouzeghrane F , Couture P , Thibault G (2007) Thy‐1 expression by cardiac fibroblasts: lack of association with myofibroblast contractile markers. J Mol Cell Cardiol 42: 991–1000 17395197 10.1016/j.yjmcc.2007.02.009

[emmm201910865-bib-0106] Hummel YM , Liu LCY , Lam CSP , Fonseca‐Munoz DF , Damman K , Rienstra M , van der Meer P , Rosenkranz S , van Veldhuisen DJ , Voors AA *et al* (2017) Echocardiographic estimation of left ventricular and pulmonary pressures in patients with heart failure and preserved ejection fraction: a study utilizing simultaneous echocardiography and invasive measurements. Eur J Heart Fail 19: 1651–1660 28984057 10.1002/ejhf.957

[emmm201910865-bib-0107] Ibarrola J , Arrieta V , Sádaba R , Martinez‐Martinez E , Garcia‐Peña A , Alvarez V , Fernández‐Celis A , Gainza A , Santamaría E , Fernández‐Irigoyen J *et al* (2018) Galectin‐3 down‐regulates antioxidant peroxiredoxin‐4 in human cardiac fibroblasts: a new pathway to induce cardiac damage. Clin Sci 132: 1471–1485 29674526 10.1042/CS20171389

[emmm201910865-bib-0108] Ide T , Tsutsui H , Kinugawa S , Suematsu N , Hayashidani S , Ichikawa K , Utsumi H , Machida Y , Egashira K , Takeshita A (2000) Direct evidence for increased hydroxyl radicals originating from superoxide in the failing myocardium. Circ Res 86: 152–157 10666410 10.1161/01.res.86.2.152

[emmm201910865-bib-0109] Ieda M , Fu J‐D , Delgado‐Olguin P , Vedantham V , Hayashi Y , Bruneau BG , Srivastava D (2010) Direct reprogramming of fibroblasts into functional cardiomyocytes by defined factors. Cell 142: 375–386 20691899 10.1016/j.cell.2010.07.002 PMC2919844

[emmm201910865-bib-0110] Ivey MJ , Tallquist MD (2016) Defining the cardiac fibroblast. Circ J 80: 2269–2276 27746422 10.1253/circj.CJ-16-1003 PMC5588900

[emmm201910865-bib-0111] Johar S , Cave AC , Narayanapanicker A , Grieve DJ , Shah AM (2006) Aldosterone mediates angiotensin II‐induced interstitial cardiac fibrosis via a Nox2‐containing NADPH oxidase. FASEB J 20: 1546–1548 16720735 10.1096/fj.05-4642fje

[emmm201910865-bib-0112] June CH , O'Connor RS , Kawalekar OU , Ghassemi S , Milone MC (2018) CAR T cell immunotherapy for human cancer. Science 359: 1361–1365 29567707 10.1126/science.aar6711

[emmm201910865-bib-0113] Kai H , Mori T , Tokuda K , Takayama N , Tahara N , Takemiya K , Kudo H , Sugi Y , Fukui D , Yasukawa H *et al* (2006) Pressure overload‐induced transient oxidative stress mediates perivascular inflammation and cardiac fibrosis through angiotensin II. Hypertens Res 29: 711–718 17249527 10.1291/hypres.29.711

[emmm201910865-bib-0114] Kalra S (2014) Sodium Glucose Co‐Transporter‐2 (SGLT2) inhibitors: a review of their basic and clinical pharmacology. Diabetes Ther 5: 355–366 25424969 10.1007/s13300-014-0089-4 PMC4269649

[emmm201910865-bib-0115] Kanagala P , Cheng ASH , Singh A , Khan JN , Gulsin GS , Patel P , Gupta P , Arnold JR , Squire IB , Ng LL *et al* (2019) Relationship between focal and diffuse fibrosis assessed by CMR and clinical outcomes in heart failure with preserved ejection fraction. JACC Cardiovasc Imaging 12: 2291–2301 30772227 10.1016/j.jcmg.2018.11.031

[emmm201910865-bib-0116] Kanefendt F , Thuss U , Becka M , Boxnick S , Berse M , Schultz A , Otto C (2019) Pharmacokinetics, safety, and tolerability of the novel chymase inhibitor BAY 1142524 in healthy male volunteers. Clin Pharmacol Drug Dev 8: 467–479 29878583 10.1002/cpdd.579

[emmm201910865-bib-0117] Kanisicak O , Khalil H , Ivey MJ , Karch J , Maliken BD , Correll RN , Brody MJ , Sc JL , Aronow BJ , Tallquist MD *et al* (2016) Genetic lineage tracing defines myofibroblast origin and function in the injured heart. Nat Commun 7: 12260 27447449 10.1038/ncomms12260 PMC5512625

[emmm201910865-bib-0118] Kasner M , Westermann D , Lopez B , Gaub R , Escher F , Kühl U , Schultheiss H‐P , Tschöpe C (2011) Diastolic tissue doppler indexes correlate with the degree of collagen expression and cross‐linking in heart failure and normal ejection fraction. J Am Coll Cardiol 57: 977 21329845 10.1016/j.jacc.2010.10.024

[emmm201910865-bib-0119] Kelly JP , Mentz RJ , Mebazaa A , Voors AA , Butler J , Roessig L , Fiuzat M , Zannad F , Pitt B , O'Connor CM *et al* (2015) Patient selection in heart failure with preserved ejection fraction clinical trials. J Am Coll Cardiol 65: 1668–1682 25908073 10.1016/j.jacc.2015.03.043 PMC4713836

[emmm201910865-bib-0120] Kerkelä R , Ulvila J , Magga J (2015) Natriuretic peptides in the regulation of cardiovascular physiology and metabolic events. J Am Heart Assoc 4: e002423 26508744 10.1161/JAHA.115.002423 PMC4845118

[emmm201910865-bib-0121] Khalil N , Corne S , Whitman C , Yacyshyn H (1996) Plasmin regulates the activation of cell‐associated latent TGF‐beta 1 secreted by rat alveolar macrophages after *in vivo* bleomycin injury. Am J Respir Cell Mol Biol 15: 252–259 8703482 10.1165/ajrcmb.15.2.8703482

[emmm201910865-bib-0122] Khalil H , Kanisicak O , Prasad V , Correll RN , Fu X , Schips T , Vagnozzi RJ , Liu R , Huynh T , Lee S‐J *et al* (2017) Fibroblast‐specific TGF‐β‐Smad2/3 signaling underlies cardiac fibrosis. J Clin Invest 127: 3770–3783 28891814 10.1172/JCI94753 PMC5617658

[emmm201910865-bib-0123] Kilvaer TK , Khanehkenari MR , Hellevik T , Al‐Saad S , Paulsen E‐E , Bremnes RM , Busund L‐T , Donnem T , Martinez IZ (2015) Cancer associated fibroblasts in stage I‐IIIA NSCLC: prognostic impact and their correlations with tumor molecular markers. PLoS ONE 10: e0134965 26252379 10.1371/journal.pone.0134965 PMC4529239

[emmm201910865-bib-0124] Kim GH , Uriel N , Burkhoff D (2018) Reverse remodelling and myocardial recovery in heart failure. Nat Rev Cardiol 15: 83–96 28933783 10.1038/nrcardio.2017.139

[emmm201910865-bib-0125] King TE , Bradford WZ , Castro‐Bernardini S , Fagan EA , Glaspole I , Glassberg MK , Gorina E , Hopkins PM , Kardatzke D , Lancaster L *et al* (2014) A Phase 3 trial of pirfenidone in patients with idiopathic pulmonary fibrosis. N Engl J Med 370: 2083–2092 24836312 10.1056/NEJMoa1402582

[emmm201910865-bib-0126] Kirimura K , Takai S , Jin D , Muramatsu M , Kishi K , Yoshikawa K , Nakabayashi M , Mino Y , Miyazaki M (2005) Role of chymase‐dependent angiotensin II formation in regulating blood pressure in spontaneously hypertensive rats. Hypertens Res 28: 457–464 16156510 10.1291/hypres.28.457

[emmm201910865-bib-0127] Kobayashi H , Kobayashi Y , Yokoe I , Akashi Y , Takei M , Giles JT (2017) Magnetic resonance imaging‐detected myocardial inflammation and fibrosis in rheumatoid arthritis: associations with disease characteristics and N‐terminal pro‐brain natriuretic peptide levels. Arthritis Care Res 69: 1304–1311 10.1002/acr.23138 27813364

[emmm201910865-bib-0128] Koitabashi N , Danner T , Zaiman AL , Pinto YM , Rowell J , Mankowski J , Zhang D , Nakamura T , Takimoto E , Kass DA (2011) Pivotal role of cardiomyocyte TGF‐beta signaling in the murine pathological response to sustained pressure overload. J Clin Invest 121: 2301–2312 21537080 10.1172/JCI44824 PMC3104748

[emmm201910865-bib-0129] Kojima M , Shiojima I , Yamazaki T , Komuro I , Zou Z , Wang Y , Mizuno T , Ueki K , Tobe K , Kadowaki T (1994) Angiotensin II receptor antagonist TCV‐116 induces regression of hypertensive left ventricular hypertrophy *in vivo* and inhibits the intracellular signaling pathway of stretch‐mediated cardiomyocyte hypertrophy *in vitro* . Circulation 89: 2204–2211 8181146 10.1161/01.cir.89.5.2204

[emmm201910865-bib-0130] Kong P , Christia P , Saxena A , Su Y , Frangogiannis NG (2013) Lack of specificity of fibroblast‐specific protein 1 in cardiac remodeling and fibrosis. Am J Physiol Heart Circ Physiol 305: H1363–H1372 23997102 10.1152/ajpheart.00395.2013 PMC3840245

[emmm201910865-bib-0131] Kosmala W , Przewlocka‐Kosmala M , Szczepanik‐Osadnik H , Mysiak A , O'Moore‐Sullivan T , Marwick TH (2011) A randomized study of the beneficial effects of aldosterone antagonism on LV function, structure, and fibrosis markers in metabolic syndrome. JACC Cardiovasc Imaging 4: 1239–1249 22172779 10.1016/j.jcmg.2011.08.014

[emmm201910865-bib-0132] Kosmala W , Marwick TH (2020) Asymptomatic left ventricular diastolic dysfunction: predicting progression to symptomatic heart failure. JACC Cardiovasc Imaging 13: 215–227 31005530 10.1016/j.jcmg.2018.10.039

[emmm201910865-bib-0133] Kotlarz D , Marquardt B , Barøy T , Lee WS , Konnikova L , Hollizeck S , Magg T , Lehle AS , Walz C , Borggraefe I *et al* (2018) Human TGF‐β1 deficiency causes severe inflammatory bowel disease and encephalopathy. Nat Genet 50: 344–348 29483653 10.1038/s41588-018-0063-6 PMC6309869

[emmm201910865-bib-0134] Kovacs RJ , Maldonado G , Azaro A , Fernández MS , Romero FL , Sepulveda‐Sánchez JM , Corretti M , Carducci M , Dolan M , Gueorguieva I *et al* (2015) Cardiac safety of TGF‐β receptor I kinase inhibitor LY2157299 monohydrate in cancer patients in a first‐in‐human dose study. Cardiovasc Toxicol 15: 309–323 25488804 10.1007/s12012-014-9297-4 PMC4575352

[emmm201910865-bib-0135] Kraigher‐Krainer E , Shah AM , Gupta DK , Santos A , Claggett B , Pieske B , Zile MR , Voors AA , Lefkowitz MP , Packer M *et al* (2014) Impaired systolic function by strain imaging in heart failure with preserved ejection fraction. J Am Coll Cardiol 63: 447–456 24184245 10.1016/j.jacc.2013.09.052 PMC7195816

[emmm201910865-bib-0136] Kramann R , Schneider RK , DiRocco DP , Machado F , Fleig S , Bondzie PA , Henderson JM , Ebert BL , Humphreys BD (2015) Perivascular Gli1 + progenitors are key contributors to injury‐induced organ fibrosis. Cell Stem Cell 16: 51–66 25465115 10.1016/j.stem.2014.11.004 PMC4289444

[emmm201910865-bib-0137] Krenning G , Zeisberg EM , Kalluri R (2010) The origin of fibroblasts and mechanism of cardiac fibrosis. J Cell Physiol 225: 631–637 20635395 10.1002/jcp.22322 PMC3098503

[emmm201910865-bib-0138] Krittayaphong R , Boonyasirinant T , Chaithiraphan V , Maneesai A , Saiviroonporn P , Nakyen S , Thanapiboonpol P , Yindeengam A , Udompanturak S (2010) Prognostic value of late gadolinium enhancement in hypertensive patients with known or suspected coronary artery disease. Int J Cardiovasc Imaging 26(Suppl 1): 123–131 10.1007/s10554-009-9574-7 20049536

[emmm201910865-bib-0139] Krüger M , Kötter S , Grützner A , Lang P , Andresen C , Redfield MM , Butt E , dos Remedios CG , Linke WA (2009) Protein kinase G modulates human myocardial passive stiffness by phosphorylation of the titin springs. Circ Res 104: 87–94 19023132 10.1161/CIRCRESAHA.108.184408

[emmm201910865-bib-0140] Krum H , Elsik M , Schneider HG , Ptaszynska A , Black M , Carson PE , Komajda M , Massie BM , McKelvie RS , McMurray JJ *et al* (2011) Relation of peripheral collagen markers to death and hospitalization in patients with heart failure and preserved ejection fraction. Circ Heart Fail 4: 561–568 21750125 10.1161/CIRCHEARTFAILURE.110.960716

[emmm201910865-bib-0141] Kulkarni AB , Huh CG , Becker D , Geiser A , Lyght M , Flanders KC , Roberts AB , Sporn MB , Ward JM , Karlsson S (1993) Transforming growth factor beta 1 null mutation in mice causes excessive inflammatory response and early death. Proc Natl Acad Sci USA 90: 770–774 8421714 10.1073/pnas.90.2.770 PMC45747

[emmm201910865-bib-0142] Kupari M , Laine M , Turto H , Lommi J , Werkkala K (2013) Circulating collagen metabolites, myocardial fibrosis and heart failure in aortic valve stenosis. J Heart Valve Dis 22: 166–176 23798204

[emmm201910865-bib-0143] Kuroda J , Ago T , Matsushima S , Zhai P , Schneider MD , Sadoshima J (2010) NADPH oxidase 4 (Nox4) is a major source of oxidative stress in the failing heart. Proc Natl Acad Sci USA 107: 15565–15570 20713697 10.1073/pnas.1002178107 PMC2932625

[emmm201910865-bib-0144] Kusaka H , Koibuchi N , Hasegawa Y , Ogawa H , Kim‐Mitsuyama S (2016) Empagliflozin lessened cardiac injury and reduced visceral adipocyte hypertrophy in prediabetic rats with metabolic syndrome. Cardiovasc Diabetol 15: 157 27835975 10.1186/s12933-016-0473-7 PMC5106779

[emmm201910865-bib-0145] Kuwahara F , Kai H , Tokuda K , Kai M , Takeshita A , Egashira K , Imaizumi T (2002) Transforming growth factor‐β function blocking prevents myocardial fibrosis and diastolic dysfunction in pressure‐overloaded rats. Circulation 106: 130–135 12093782 10.1161/01.cir.0000020689.12472.e0

[emmm201910865-bib-0146] Kwak H‐B , Lee Y , Kim J‐H , Van Remmen H , Richardson AG , Lawler JM (2015) MnSOD overexpression reduces fibrosis and pro‐apoptotic signaling in the aging mouse heart. J Gerontol A Biol Sci Med Sci 70: 533–544 25016531 10.1093/gerona/glu090 PMC4462657

[emmm201910865-bib-0147] Kwong RY , Sattar H , Wu H , Vorobiof G , Gandla V , Steel K , Siu S , Brown KA (2008) Incidence and prognostic implication of unrecognized myocardial scar characterized by cardiac magnetic resonance in diabetic patients without clinical evidence of myocardial infarction. Circulation 118: 1011–1020 18725488 10.1161/CIRCULATIONAHA.107.727826 PMC2743310

[emmm201910865-bib-0148] Lacouture ME , Morris JC , Lawrence DP , Tan AR , Olencki TE , Shapiro GI , Dezube BJ , Berzofsky JA , Hsu FJ , Guitart J (2015) Cutaneous keratoacanthomas/squamous cell carcinomas associated with neutralization of transforming growth factor β by the monoclonal antibody fresolimumab (GC1008). Cancer Immunol Immunother 64: 437–446 25579378 10.1007/s00262-015-1653-0 PMC6730642

[emmm201910865-bib-0149] Lancaster LH , de Andrade JA , Zibrak J , Padilla ML , Albera C , Nathan SD , Wijsenbeek MS , Stauffer JL , Kirchgaessler K‐U , Costabel U (2017) Pirfenidone safety and adverse event management in idiopathic pulmonary fibrosis. Eur Respir Rev 26: 170057 29212837 10.1183/16000617.0057-2017 PMC9488585

[emmm201910865-bib-0150] Lassègue B , San Martín A , Griendling KK (2012) Biochemistry, physiology, and pathophysiology of NADPH oxidases in the cardiovascular system. Circ Res 110: 1364–1390 22581922 10.1161/CIRCRESAHA.111.243972 PMC3365576

[emmm201910865-bib-0151] Leader CJ , Moharram M , Coffey S , Sammut IA , Wilkins GW , Walker RJ (2019) Myocardial global longitudinal strain: an early indicator of cardiac interstitial fibrosis modified by spironolactone, in a unique hypertensive rat model. PLoS ONE 14: e0220837 31404095 10.1371/journal.pone.0220837 PMC6690508

[emmm201910865-bib-0152] Lebastchi AH , Qin L , Khan SF , Zhou J , Geirsson A , Kim RW , Li W , Tellides G (2011) Activation of human vascular cells decreases their expression of transforming growth factor‐beta. Atherosclerosis 219: 417–424 21862019 10.1016/j.atherosclerosis.2011.07.121 PMC3226933

[emmm201910865-bib-0153] Lee MK , Pardoux C , Hall MC , Lee PS , Warburton D , Qing J , Smith SM , Derynck R (2007) TGF‐beta activates Erk MAP kinase signalling through direct phosphorylation of ShcA. EMBO J 26: 3957–3967 17673906 10.1038/sj.emboj.7601818 PMC1994119

[emmm201910865-bib-0154] Lee H‐C , Shiou Y‐L , Jhuo S‐J , Chang C‐Y , Liu P‐L , Jhuang W‐J , Dai Z‐K , Chen W‐Y , Chen Y‐F , Lee A‐S (2019) The sodium‐glucose co‐transporter 2 inhibitor empagliflozin attenuates cardiac fibrosis and improves ventricular hemodynamics in hypertensive heart failure rats. Cardiovasc Diabetol 18: 45 30935417 10.1186/s12933-019-0849-6 PMC6444638

[emmm201910865-bib-0155] Lemarié CA , Simeone SMC , Nikonova A , Ebrahimian T , Deschênes M‐E , Coffman TM , Paradis P , Schiffrin EL (2009) Aldosterone‐induced activation of signaling pathways requires activity of angiotensin type 1a receptors. Circ Res 105: 852–859 19762686 10.1161/CIRCRESAHA.109.196576

[emmm201910865-bib-0156] Lesyuk W , Kriza C , Kolominsky‐Rabas P (2018) Cost‐of‐illness studies in heart failure: a systematic review 2004–2016. BMC Cardiovasc Disord 18: 74 29716540 10.1186/s12872-018-0815-3 PMC5930493

[emmm201910865-bib-0157] Lewis GA , Schelbert EB , Naish JH , Bedson E , Dodd S , Eccleson H , Clayton D , Jimenez BD , McDonagh T , Williams SG *et al* (2019) Pirfenidone in heart failure with preserved ejection fraction‐rationale and design of the PIROUETTE trial. Cardiovasc Drugs Ther 33: 461–470 31069575 10.1007/s10557-019-06876-y PMC6689029

[emmm201910865-bib-0158] Li Y , Huang TT , Carlson EJ , Melov S , Ursell PC , Olson JL , Noble LJ , Yoshimura MP , Berger C , Chan PH *et al* (1995) Dilated cardiomyopathy and neonatal lethality in mutant mice lacking manganese superoxide dismutase. Nat Genet 11: 376–381 7493016 10.1038/ng1295-376

[emmm201910865-bib-0159] Li RK , Li G , Mickle DA , Weisel RD , Merante F , Luss H , Rao V , Christakis GT , Williams WG (1997) Overexpression of transforming growth factor‐beta1 and insulin‐like growth factor‐I in patients with idiopathic hypertrophic cardiomyopathy. Circulation 96: 874–881 9264495 10.1161/01.cir.96.3.874

[emmm201910865-bib-0160] Li C , Sun X‐N , Zeng M‐R , Zheng X‐J , Zhang Y‐Y , Wan Q , Zhang W‐C , Shi C , Du L‐J , Ai T‐J *et al* (2017) Mineralocorticoid receptor deficiency in T cells attenuates pressure overload‐induced cardiac hypertrophy and dysfunction through modulating T‐cell activation. Hypertension 70: 137–147 28559389 10.1161/HYPERTENSIONAHA.117.09070

[emmm201910865-bib-0161] Li C , Zhang J , Xue M , Li X , Han F , Liu X , Xu L , Lu Y , Cheng Y , Li T *et al* (2019) SGLT2 inhibition with empagliflozin attenuates myocardial oxidative stress and fibrosis in diabetic mice heart. Cardiovasc Diabetol 18: 15 30710997 10.1186/s12933-019-0816-2 PMC6359811

[emmm201910865-bib-0162] Liang F , Gardner DG (1999) Mechanical strain activates BNP gene transcription through a p38/NF‐kappaB‐dependent mechanism. J Clin Invest 104: 1603–1612 10587524 10.1172/JCI7362 PMC409860

[emmm201910865-bib-0163] Lijnen P , Papparella I , Petrov V , Semplicini A , Fagard R (2006) Angiotensin II‐stimulated collagen production in cardiac fibroblasts is mediated by reactive oxygen species. J Hypertens 24: 757–766 16531806 10.1097/01.hjh.0000217860.04994.54

[emmm201910865-bib-0164] Liu J , Masurekar MR , Vatner DE , Jyothirmayi GN , Regan TJ , Vatner SF , Meggs LG , Malhotra A (2003) Glycation end‐product cross‐link breaker reduces collagen and improves cardiac function in aging diabetic heart. Am J Physiol Heart Circ Physiol 285: H2587–H2591 12946933 10.1152/ajpheart.00516.2003

[emmm201910865-bib-0165] Liu N‐W , Huang X , Liu S , Liu W‐J , Wang H , Wang W , Lu Y (2019) Elevated BNP caused by recombinant human interleukin‐11 treatment in patients with chemotherapy‐induced thrombocytopenia. Support Care Cancer 27: 4293–4298 30877597 10.1007/s00520-019-04734-z PMC6803615

[emmm201910865-bib-0166] Löffler AI , Pan JA , Balfour PC Jr , Shaw PW , Yang Y , Nasir M , Auger DA , Epstein FH , Kramer CM , Gan L‐M *et al* (2019) Frequency of coronary microvascular dysfunction and diffuse myocardial fibrosis (measured by cardiovascular magnetic resonance) in patients with heart failure and preserved left ventricular ejection fraction. Am J Cardiol 124: 1584–1589 31575425 10.1016/j.amjcard.2019.08.011 PMC6894613

[emmm201910865-bib-0167] Lombardi R , Betocchi S , Losi MA , Tocchetti CG , Aversa M , Miranda M , D'Alessandro G , Cacace A , Ciampi Q , Chiariello M (2003) Myocardial collagen turnover in hypertrophic cardiomyopathy. Circulation 108: 1455–1460 12952838 10.1161/01.CIR.0000090687.97972.10

[emmm201910865-bib-0168] López B , González A , Querejeta R , Larman M , Díez J (2006) Alterations in the pattern of collagen deposition may contribute to the deterioration of systolic function in hypertensive patients with heart failure. J Am Coll Cardiol 48: 89–96 16814653 10.1016/j.jacc.2006.01.077

[emmm201910865-bib-0169] López B , González A , Querejeta R , Larman M , Rábago G , Díez J (2014) Association of cardiotrophin‐1 with myocardial fibrosis in hypertensive patients with heart failure. Hypertension 63: 483–489 24366078 10.1161/HYPERTENSIONAHA.113.02654

[emmm201910865-bib-0170] López B , González A , Querejeta R , Zubillaga E , Larman M , Díez J (2015a) Galectin‐3 and histological, molecular and biochemical aspects of myocardial fibrosis in heart failure of hypertensive origin. Eur J Heart Fail 17: 385–392 25684565 10.1002/ejhf.246

[emmm201910865-bib-0171] López B , González A , Ravassa S , Beaumont J , Moreno MU , San José G , Querejeta R , Díez J (2015b) Circulating biomarkers of myocardial fibrosis: the need for a reappraisal. J Am Coll Cardiol 65: 2449–2456 26046739 10.1016/j.jacc.2015.04.026

[emmm201910865-bib-0172] López B , Ravassa S , González A , Zubillaga E , Bonavila C , Bergés M , Echegaray K , Beaumont J , Moreno MU , San José G *et al* (2016) Myocardial collagen cross‐linking is associated with heart failure hospitalization in patients with hypertensive heart failure. J Am Coll Cardiol 67: 251 26796388 10.1016/j.jacc.2015.10.063

[emmm201910865-bib-0173] Lother A , Berger S , Gilsbach R , Rösner S , Ecke A , Barreto F , Bauersachs J , Schütz G , Hein L (2011) Ablation of mineralocorticoid receptors in myocytes but not in fibroblasts preserves cardiac function. Hypertension 57: 746–754 21321305 10.1161/HYPERTENSIONAHA.110.163287

[emmm201910865-bib-0174] Mallat Z , Philip I , Lebret M , Chatel D , Maclouf J , Tedgui A (1998) Elevated levels of 8‐iso‐prostaglandin F2alpha in pericardial fluid of patients with heart failure: a potential role for *in vivo* oxidant stress in ventricular dilatation and progression to heart failure. Circulation 97: 1536–1539 9593557 10.1161/01.cir.97.16.1536

[emmm201910865-bib-0175] Markos F , Healy V , Harvey BJ (2005) Aldosterone rapidly activates Na+/H+ exchange in M‐1 cortical collecting duct cells via a PKC‐MAPK pathway. Nephron Physiol 99: 1–9 10.1159/000081796 15637466

[emmm201910865-bib-0176] Martin J , Kelly DJ , Mifsud SA , Zhang Y , Cox AJ , See F , Krum H , Wilkinson‐Berka J , Gilbert RE (2005) Tranilast attenuates cardiac matrix deposition in experimental diabetes: role of transforming growth factor‐beta. Cardiovasc Res 65: 694–701 15664396 10.1016/j.cardiores.2004.10.041

[emmm201910865-bib-0177] Martin N , Manoharan K , Thomas J , Davies C , Lumbers RT (2018) Beta‐blockers and inhibitors of the renin‐angiotensin aldosterone system for chronic heart failure with preserved ejection fraction. Cochrane Database Syst Rev 6: CD012721 29952095 10.1002/14651858.CD012721.pub2 PMC6513293

[emmm201910865-bib-0178] Martínez‐Martínez E , López‐Ándres N , Jurado‐López R , Rousseau E , Bartolomé Mará V , Fernández‐Celis A , Rossignol P , Islas F , Antequera A , Prieto S *et al* (2015) Galectin‐3 participates in cardiovascular remodeling associated with obesity. Hypertension 66: 961–969 26351031 10.1161/HYPERTENSIONAHA.115.06032

[emmm201910865-bib-0179] Martin‐Malpartida P , Batet M , Kaczmarska Z , Freier R , Gomes T , Aragón E , Zou Y , Wang Q , Xi Q , Ruiz L *et al* (2017) Structural basis for genome wide recognition of 5‐bp GC motifs by SMAD transcription factors. Nat Commun 8: 2070 29234012 10.1038/s41467-017-02054-6 PMC5727232

[emmm201910865-bib-0180] Martos R , Baugh J , Ledwidge M , O'Loughlin C , Murphy NF , Conlon C , Patle A , Donnelly SC , McDonald K (2009) Diagnosis of heart failure with preserved ejection fraction: improved accuracy with the use of markers of collagen turnover. Eur J Heart Fail 11: 191–197 19168518 10.1093/eurjhf/hfn036 PMC2639413

[emmm201910865-bib-0181] Mascherbauer J , Marzluf BA , Tufaro C , Pfaffenberger S , Graf A , Wexberg P , Panzenböck A , Jakowitsch J , Bangert C , Laimer D *et al* (2013) Cardiac magnetic resonance postcontrast T1 time is associated with outcome in patients with heart failure and preserved ejection fraction. Circ Cardiovasc Imaging 6: 1056–1065 24036385 10.1161/CIRCIMAGING.113.000633

[emmm201910865-bib-0182] Matsumoto T , Wada A , Tsutamoto T , Ohnishi M , Isono T , Kinoshita M (2003) Chymase inhibition prevents cardiac fibrosis and improves diastolic dysfunction in the progression of heart failure. Circulation 107: 2555–2558 12742989 10.1161/01.CIR.0000074041.81728.79

[emmm201910865-bib-0183] Matsumoto‐Ida M , Takimoto Y , Aoyama T , Akao M , Takeda T , Kita T (2006) Activation of TGF‐β1‐TAK1‐p38 MAPK pathway in spared cardiomyocytes is involved in left ventricular remodeling after myocardial infarction in rats. Am J Physiol Heart Circ Physiol 290: H709–H715 16183734 10.1152/ajpheart.00186.2005

[emmm201910865-bib-0184] Matsushima S , Kinugawa S , Sadoshima J , Tsutsui H (2016) NADPH oxidase 4 is a major source of mitochondrial reactive oxygen species in the heart. J Card Fail 22: S159

[emmm201910865-bib-0185] McDiarmid AK , Swoboda PP , Erhayiem B , Bounford KA , Bijsterveld P , Tyndall K , Fent GJ , Garg P , Dobson LE , Musa TA *et al* (2020) Myocardial effects of aldosterone antagonism in heart failure with preserved ejection fraction. J Am Heart Assoc 9: e011521 31852424 10.1161/JAHA.118.011521 PMC6988171

[emmm201910865-bib-0186] McDonald PH , Chow CW , Miller WE , Laporte SA , Field ME , Lin FT , Davis RJ , Lefkowitz RJ (2000) Beta‐arrestin 2: a receptor‐regulated MAPK scaffold for the activation of JNK3. Science 290: 1574–1577 11090355 10.1126/science.290.5496.1574

[emmm201910865-bib-0187] McMurray JJV , O'Connor C (2014) Lessons from the TOPCAT Trial. N Engl J Med 370: 1453–1454 24716685 10.1056/NEJMe1401231

[emmm201910865-bib-0188] McMurray JJV , Packer M , Desai AS , Gong J , Lefkowitz MP , Rizkala AR , Rouleau JL , Shi VC , Solomon SD , Swedberg K *et al* (2014) Angiotensin‐neprilysin inhibition versus enalapril in heart failure. N Engl J Med 371: 993–1004 25176015 10.1056/NEJMoa1409077

[emmm201910865-bib-0189] McMurray JJV , Solomon SD , Inzucchi SE , Køber L , Kosiborod MN , Martinez FA , Ponikowski P , Sabatine MS , Anand IS , Bělohlávek J *et al* (2019) Dapagliflozin in patients with heart failure and reduced ejection fraction. N Engl J Med 381: 1995–2008 31535829 10.1056/NEJMoa1911303

[emmm201910865-bib-0190] Meng X‐M , Nikolic‐Paterson DJ , Lan HY (2016) TGF‐β: the master regulator of fibrosis. Nat Rev Nephrol 12: 325–338 27108839 10.1038/nrneph.2016.48

[emmm201910865-bib-0191] Mihailidou AS , Mardini M , Funder JW (2004) Rapid, nongenomic effects of aldosterone in the heart mediated by epsilon protein kinase C. Endocrinology 145: 773–780 14605011 10.1210/en.2003-1137

[emmm201910865-bib-0192] Mitra MS , Lancaster K , Adedeji AO , Palanisamy GS , Dave RA , Zhong F , Holdren MH , Turley SJ , Liang W‐C , Wu Y *et al* (2020) A potent Pan‐TGFβ neutralizing monoclonal antibody elicits cardiovascular toxicity in mice and cynomolgus monkeys. Toxicol Sci 175: 24–34 32077954 10.1093/toxsci/kfaa024

[emmm201910865-bib-0193] Molkentin JD , Bugg D , Ghearing N , Dorn LE , Kim P , Sargent MA , Gunaje J , Otsu K , Davis J (2017) Fibroblast‐specific genetic manipulation of p38 mitogen‐activated protein kinase *in vivo* reveals its central regulatory role in fibrosis. Circulation 136: 549–561 28356446 10.1161/CIRCULATIONAHA.116.026238 PMC5548661

[emmm201910865-bib-0194] Mondorf UF , Geiger H , Herrero M , Zeuzem S , Piiper A (2000) Involvement of the platelet‐derived growth factor receptor in angiotensin II‐induced activation of extracellular regulated kinases 1 and 2 in human mesangial cells. FEBS Lett 472: 129–132 10781819 10.1016/s0014-5793(00)01433-2

[emmm201910865-bib-0195] Moore‐Morris T , Guimaraes‐Camboa N , Banerjee I , Zambon AC , Kisseleva T , Velayoudon A , Stallcup WB , Gu Y , Dalton ND , Cedenilla M *et al* (2014) Resident fibroblast lineages mediate pressure overload‐induced cardiac fibrosis. J Clin Invest 124: 2921–2934 24937432 10.1172/JCI74783 PMC4071409

[emmm201910865-bib-0196] Moravsky G , Ofek E , Rakowski H , Butany J , Williams L , Ralph‐Edwards A , Wintersperger BJ , Crean A (2013) Myocardial fibrosis in hypertrophic cardiomyopathy: accurate reflection of histopathological findings by CMR. JACC Cardiovasc Imaging 6: 587–596 23582356 10.1016/j.jcmg.2012.09.018

[emmm201910865-bib-0197] Moreo A , Ambrosio G , De Chiara B , Pu M , Tran T , Mauri F , Raman SV (2009) Influence of myocardial fibrosis on left ventricular diastolic function. Circ Cardiovasc Imaging 2: 437–443 19920041 10.1161/CIRCIMAGING.108.838367 PMC2782553

[emmm201910865-bib-0198] Moris D , Spartalis M , Tzatzaki E , Spartalis E , Karachaliou G‐S , Triantafyllis AS , Karaolanis GI , Tsilimigras DI , Theocharis S (2017) The role of reactive oxygen species in myocardial redox signaling and regulation. Ann Transl Med 5: 324 28861421 10.21037/atm.2017.06.17 PMC5566737

[emmm201910865-bib-0199] Mullen AC , Orlando DA , Newman JJ , Lovén J , Kumar RM , Bilodeau S , Reddy J , Guenther MG , DeKoter RP , Young RA (2011) Master transcription factors determine cell‐type‐specific responses to TGF‐β signaling. Cell 147: 565–576 22036565 10.1016/j.cell.2011.08.050 PMC3212730

[emmm201910865-bib-0200] Munger JS , Huang X , Kawakatsu H , Griffiths MJ , Dalton SL , Wu J , Pittet JF , Kaminski N , Garat C , Matthay MA *et al* (1999) The integrin alpha v beta 6 binds and activates latent TGF beta 1: a mechanism for regulating pulmonary inflammation and fibrosis. Cell 96: 319–328 10025398 10.1016/s0092-8674(00)80545-0

[emmm201910865-bib-0201] Nabeebaccus AA , Zoccarato A , Hafstad AD , Santos CX , Aasum E , Brewer AC , Zhang M , Beretta M , Yin X , West JA *et al* (2017) Nox4 reprograms cardiac substrate metabolism via protein O‐GlcNAcylation to enhance stress adaptation. JCI Insight 2: e96184 10.1172/jci.insight.96184 PMC5752273 29263294

[emmm201910865-bib-0202] Nagao K , Inada T , Tamura A , Kajitani K , Shimamura K , Yukawa H , Aida K , Sowa N , Nishiga M , Horie T *et al* (2018) Circulating markers of collagen types I, III, and IV in patients with dilated cardiomyopathy: relationships with myocardial collagen expression. ESC Heart Fail 5: 1044–1051 30273997 10.1002/ehf2.12360 PMC6301156

[emmm201910865-bib-0203] Nagaraju CK , Robinson EL , Abdesselem M , Trenson S , Dries E , Gilbert G , Janssens S , Van Cleemput J , Rega F , Meyns B *et al* (2019) Myofibroblast phenotype and reversibility of fibrosis in patients with end‐stage heart failure. J Am Coll Cardiol 73: 2267–2282 31072570 10.1016/j.jacc.2019.02.049

[emmm201910865-bib-0204] Nakajima H , Nakajima HO , Salcher O , Dittie AS , Dembowsky K , Jing S , Field LJ (2000) Atrial but not ventricular fibrosis in mice expressing a mutant transforming growth factor‐beta(1) transgene in the heart. Circ Res 86: 571–579 10720419 10.1161/01.res.86.5.571

[emmm201910865-bib-0205] Nakatani Y , Nishida K , Sakabe M , Kataoka N , Sakamoto T , Yamaguchi Y , Iwamoto J , Mizumaki K , Fujiki A , Inoue H (2013) Tranilast prevents atrial remodeling and development of atrial fibrillation in a canine model of atrial tachycardia and left ventricular dysfunction. J Am Coll Cardiol 61: 582–588 23273396 10.1016/j.jacc.2012.11.014

[emmm201910865-bib-0206] Nandurkar HH , Robb L , Tarlinton D , Barnett L , Köntgen F , Begley CG (1997) Adult mice with targeted mutation of the interleukin‐11 receptor (IL11Ra) display normal hematopoiesis. Blood 90: 2148–2159 9310465

[emmm201910865-bib-0207] Neal B , Perkovic V , Mahaffey KW , de Zeeuw D , Fulcher G , Erondu N , Shaw W , Law G , Desai M , Matthews DR *et al* (2017) Canagliflozin and cardiovascular and renal events in type 2 diabetes. N Engl J Med 377: 644–657 28605608 10.1056/NEJMoa1611925

[emmm201910865-bib-0208] Nediani C , Borchi E , Giordano C , Baruzzo S , Ponziani V , Sebastiani M , Nassi P , Mugelli A , d'Amati G , Cerbai E (2007) NADPH oxidase‐dependent redox signaling in human heart failure: relationship between the left and right ventricle. J Mol Cell Cardiol 42: 826–834 17346742 10.1016/j.yjmcc.2007.01.009

[emmm201910865-bib-0209] Nevers T , Salvador AM , Velazquez F , Ngwenyama N , Carrillo‐Salinas FJ , Aronovitz M , Blanton RM , Alcaide P (2017) Th1 effector T cells selectively orchestrate cardiac fibrosis in nonischemic heart failure. J Exp Med 214: 3311–3329 28970239 10.1084/jem.20161791 PMC5679176

[emmm201910865-bib-0210] Ng B , Dong J , D'Agostino G , Viswanathan S , Widjaja AA , Lim W‐W , Ko NSJ , Tan J , Chothani SP , Huang B *et al* (2019) Interleukin‐11 is a therapeutic target in idiopathic pulmonary fibrosis. Sci Transl Med 11: e1237 10.1126/scitranslmed.aaw1237 31554736

[emmm201910865-bib-0211] Nigri M , Azevedo CF , Rochitte CE , Schraibman V , Tarasoutchi F , Pommerantzeff PM , Brandão CM , Sampaio RO , Parga JR , Avila LF *et al* (2009) Contrast‐enhanced magnetic resonance imaging identifies focal regions of intramyocardial fibrosis in patients with severe aortic valve disease: correlation with quantitative histopathology. Am Heart J 157: 361–368 19185646 10.1016/j.ahj.2008.09.012

[emmm201910865-bib-0212] Nishioka T , Suzuki M , Onishi K , Takakura N , Inada H , Yoshida T , Hiroe M , Imanaka‐Yoshida K (2007) Eplerenone attenuates myocardial fibrosis in the angiotensin II‐induced hypertensive mouse: involvement of tenascin‐C induced by aldosterone‐mediated inflammation. J Cardiovasc Pharmacol 49: 261–268 17513943 10.1097/FJC.0b013e318033dfd4

[emmm201910865-bib-0213] Norton GR , Tsotetsi J , Trifunovic B , Hartford C , Candy GP , Woodiwiss AJ (1997) Myocardial stiffness is attributed to alterations in cross‐linked collagen rather than total collagen or phenotypes in spontaneously hypertensive rats. Circulation 96: 1991–1998 9323091 10.1161/01.cir.96.6.1991

[emmm201910865-bib-0214] Obana M , Maeda M , Takeda K , Hayama A , Mohri T , Yamashita T , Nakaoka Y , Komuro I , Takeda K , Matsumiya G *et al* (2010) Therapeutic activation of signal transducer and activator of transcription 3 by interleukin‐11 ameliorates cardiac fibrosis after myocardial infarction. Circulation 121: 684–691 20100971 10.1161/CIRCULATIONAHA.109.893677

[emmm201910865-bib-0215] Obana M , Miyamoto K , Murasawa S , Iwakura T , Hayama A , Yamashita T , Shiragaki M , Kumagai S , Miyawaki A , Takewaki K *et al* (2012) Therapeutic administration of IL‐11 exhibits the postconditioning effects against ischemia‐reperfusion injury via STAT3 in the heart. Am J Physiol Heart Circ Physiol 303: H569–H577 22707562 10.1152/ajpheart.00060.2012

[emmm201910865-bib-0216] O'Connor CM , Starling RC , Hernandez AF , Armstrong PW , Dickstein K , Hasselblad V , Heizer GM , Komajda M , Massie BM , McMurray JJV *et al* (2011) Effect of nesiritide in patients with acute decompensated heart failure. N Engl J Med 365: 32–43 21732835 10.1056/NEJMoa1100171

[emmm201910865-bib-0217] Oh CM , Cho S , Jang JY , Kim H , Chun S , Choi M , Park S , Ko YG (2019) Cardioprotective potential of an SGLT2 inhibitor against doxorubicin‐induced heart failure. Korean Circ J 49: 1183–1195 31456369 10.4070/kcj.2019.0180 PMC6875592

[emmm201910865-bib-0218] Okayama K , Azuma J , Dosaka N , Iekushi K , Sanada F , Kusunoki H , Iwabayashi M , Rakugi H , Taniyama Y , Morishita R (2012) Hepatocyte growth factor reduces cardiac fibrosis by inhibiting endothelial‐mesenchymal transition. Hypertension 59: 958–965 22392903 10.1161/HYPERTENSIONAHA.111.183905

[emmm201910865-bib-0219] Okoshi MP , Yan X , Okoshi K , Nakayama M , Schuldt AJT , O'Connell TD , Simpson PC , Lorell BH (2004) Aldosterone directly stimulates cardiac myocyte hypertrophy. J Card Fail 10: 511–518 15599842 10.1016/j.cardfail.2004.03.002

[emmm201910865-bib-0220] O'Neill S , Brault J , Stasia M‐J , Knaus UG (2015) Genetic disorders coupled to ROS deficiency. Redox Biol 6: 135–156 26210446 10.1016/j.redox.2015.07.009 PMC4550764

[emmm201910865-bib-0221] Osorio H , Coronel I , Arellano A , Pacheco U , Bautista R , Franco M , Escalante B (2012) Sodium‐glucose cotransporter inhibition prevents oxidative stress in the kidney of diabetic rats. Oxid Med Cell Longev 2012: 542042 23227274 10.1155/2012/542042 PMC3512343

[emmm201910865-bib-0222] Pahor M , Bernabei R , Sgadari A , Gambassi G Jr , Lo Giudice P , Pacifici L , Ramacci MT , Lagrasta C , Olivetti G , Carbonin P (1991) Enalapril prevents cardiac fibrosis and arrhythmias in hypertensive rats. Hypertension 18: 148–157 1885222 10.1161/01.hyp.18.2.148

[emmm201910865-bib-0223] Palaniyandi SS , Nagai Y , Watanabe K , Ma M , Veeraveedu PT , Prakash P , Kamal FA , Abe Y , Yamaguchi K , Tachikawa H *et al* (2007) Chymase inhibition reduces the progression to heart failure after autoimmune myocarditis in rats. Exp Biol Med 232: 1213–1221 10.3181/0703-RM-85 17895529

[emmm201910865-bib-0224] Park JH , Rivière I , Gonen M , Wang X , Sénéchal B , Curran KJ , Sauter C , Wang Y , Santomasso B , Mead E *et al* (2018) Long‐term follow‐up of CD19 CAR therapy in acute lymphoblastic leukemia. N Engl J Med 378: 449–459 29385376 10.1056/NEJMoa1709919 PMC6637939

[emmm201910865-bib-0225] Parthasarathy A , Gopi V , Umadevi S , Simna A , Sheik MJ , Divya H , Vellaichamy E (2013) Suppression of atrial natriuretic peptide/natriuretic peptide receptor‐A‐mediated signaling upregulates angiotensin‐II‐induced collagen synthesis in adult cardiac fibroblasts. Mol Cell Biochem 378: 217–228 23526266 10.1007/s11010-013-1612-z

[emmm201910865-bib-0226] Patel JB , Valencik ML , Pritchett AM , Burnett JC , McDonald JA , Redfield MM (2005) Cardiac‐specific attenuation of natriuretic peptide A receptor activity accentuates adverse cardiac remodeling and mortality in response to pressure overload. Am J Physiol Heart Circ Physiol 289: H777–H784 15778276 10.1152/ajpheart.00117.2005

[emmm201910865-bib-0227] Paulus WJ , Tschöpe C (2013) A novel paradigm for heart failure with preserved ejection fraction: comorbidities drive myocardial dysfunction and remodeling through coronary microvascular endothelial inflammation. J Am Coll Cardiol 62: 263–271 23684677 10.1016/j.jacc.2013.02.092

[emmm201910865-bib-0228] Peet C , Ivetic A , Bromage DI , Shah AM (2019) Cardiac monocytes and macrophages after myocardial infarction. Cardiovasc Res 116: 1101–1112 10.1093/cvr/cvz336 PMC7177720 31841135

[emmm201910865-bib-0229] Pfeffer MA , Braunwald E , Moyé LA , Basta L , Brown EJ , Cuddy TE , Davis BR , Geltman EM , Goldman S , Flaker GC *et al* (1992) Effect of captopril on mortality and morbidity in patients with left ventricular dysfunction after myocardial infarction. N Engl J Med 327: 669–677 1386652 10.1056/NEJM199209033271001

[emmm201910865-bib-0230] Pfeffer MA , Shah AM , Borlaug BA (2019) Heart failure with preserved ejection fraction in perspective. Circ Res 124: 1598–1617 31120821 10.1161/CIRCRESAHA.119.313572 PMC6534165

[emmm201910865-bib-0231] Picchi A , Gao X , Belmadani S , Potter BJ , Focardi M , Chilian WM , Zhang C (2006) Tumor necrosis factor‐alpha induces endothelial dysfunction in the prediabetic metabolic syndrome. Circ Res 99: 69–77 16741160 10.1161/01.RES.0000229685.37402.80

[emmm201910865-bib-0232] Pikkarainen S , Tokola H , Kerkela R , Majalahti‐Palviainen T , Vuolteenaho O , Ruskoaho H (2003) Endothelin‐1‐specific activation of B‐type natriuretic peptide gene via p38 mitogen‐activated protein kinase and nuclear ETS factors. J Biol Chem 278: 3969–3975 12446726 10.1074/jbc.M205616200

[emmm201910865-bib-0233] Pinto AR , Ilinykh A , Ivey MJ , Kuwabara JT , D'Antoni ML , Debuque R , Chandran A , Wang L , Arora K , Rosenthal NA *et al* (2016) Revisiting cardiac cellular composition. Circ Res 118: 400–409 26635390 10.1161/CIRCRESAHA.115.307778 PMC4744092

[emmm201910865-bib-0234] Pitt B , Zannad F , Remme WJ , Cody R , Castaigne A , Perez A , Palensky J , Wittes J (1999) The effect of spironolactone on morbidity and mortality in patients with severe heart failure. N Engl J Med 341: 709–717 10471456 10.1056/NEJM199909023411001

[emmm201910865-bib-0235] Pitt B , Pfeffer MA , Assmann SF , Boineau R , Anand IS , Claggett B , Clausell N , Desai AS , Diaz R , Fleg JL *et al* (2014) Spironolactone for heart failure with preserved ejection fraction. N Engl J Med 370: 1383–1392 24716680 10.1056/NEJMoa1313731

[emmm201910865-bib-0236] Polejaeva IA , Ranjan R , Davies CJ , Regouski M , Hall J , Olsen AL , Meng Q , Rutigliano HM , Dosdall DJ , Angel NA *et al* (2016) Increased susceptibility to atrial fibrillation secondary to atrial fibrosis in transgenic goats expressing transforming growth factor‐β1. J Cardiovasc Electrophysiol 27: 1220–1229 27447370 10.1111/jce.13049 PMC5065395

[emmm201910865-bib-0237] Ponikowski P , Anker SD , AlHabib KF , Cowie MR , Force TL , Hu S , Jaarsma T , Krum H , Rastogi V , Rohde LE *et al* (2014) Heart failure: preventing disease and death worldwide. ESC Heart Fail 1: 4–25 28834669 10.1002/ehf2.12005

[emmm201910865-bib-0238] Ponikowski P , Voors AA , Anker SD , Bueno H , Cleland JGF , Coats AJS , Falk V , González‐Juanatey JR , Harjola V‐P , Jankowska EA *et al* (2016) 2016 ESC Guidelines for the diagnosis and treatment of acute and chronic heart failure: The Task Force for the diagnosis and treatment of acute and chronic heart failure of the European Society of Cardiology (ESC) Developed with the special contribution of the Heart Failure Association (HFA) of the ESC. Eur Heart J 37: 2129–2200 27206819 10.1093/eurheartj/ehw128

[emmm201910865-bib-0239] Porter DL , Levine BL , Kalos M , Bagg A , June CH (2011) Chimeric antigen receptor‐modified T cells in chronic lymphoid leukemia. N Engl J Med 365: 725–733 21830940 10.1056/NEJMoa1103849 PMC3387277

[emmm201910865-bib-0240] Prosser HCG , Forster ME , Richards AM , Pemberton CJ (2009) Cardiac chymase converts rat proAngiotensin‐12 (PA12) to angiotensin II: effects of PA12 upon cardiac haemodynamics. Cardiovasc Res 82: 40–50 19147651 10.1093/cvr/cvp003

[emmm201910865-bib-0241] Qian L , Huang Y , Spencer CI , Foley A , Vedantham V , Liu L , Conway SJ , Fu J‐D , Srivastava D (2012) *In vivo* reprogramming of murine cardiac fibroblasts into induced cardiomyocytes. Nature 485: 593–598 22522929 10.1038/nature11044 PMC3369107

[emmm201910865-bib-0242] Qin F , Shite J , Liang C‐S (2003) Antioxidants attenuate myocyte apoptosis and improve cardiac function in CHF: association with changes in MAPK pathways. Am J Physiol Heart Circ Physiol 285: H822–H832 12714335 10.1152/ajpheart.00015.2003

[emmm201910865-bib-0243] Querejeta R , Varo N , López B , Larman M , Artiñano E , Etayo JC , Ubago JLM , Gutierrez‐Stampa M , Emparanza JI , Gil MJ *et al* (2000) Serum carboxy‐terminal propeptide of procollagen type I is a marker of myocardial fibrosis in hypertensive heart disease. Circulation 101: 1729–1735 10758057 10.1161/01.cir.101.14.1729

[emmm201910865-bib-0244] Querejeta R , López B , González A , Sánchez E , Larman M , Martínez Ubago JL , Díez J (2004) Increased collagen type I synthesis in patients with heart failure of hypertensive origin. Circulation 110: 1263–1268 15313958 10.1161/01.CIR.0000140973.60992.9A

[emmm201910865-bib-0245] Rakesh K , Yoo B , Kim I‐M , Salazar N , Kim K‐S , Rockman HA (2010) beta‐Arrestin‐biased agonism of the angiotensin receptor induced by mechanical stress. Sci Signal 3: ra46 20530803 10.1126/scisignal.2000769 PMC2981501

[emmm201910865-bib-0246] Rangarajan S , Kurundkar A , Kurundkar D , Bernard K , Sanders YY , Ding Q , Antony VB , Zhang J , Zmijewski J , Thannickal VJ (2016) Novel mechanisms for the antifibrotic action of nintedanib. Am J Respir Cell Mol Biol 54: 51–59 26072676 10.1165/rcmb.2014-0445OC PMC4742925

[emmm201910865-bib-0247] Ravassa S , Trippel T , Bach D , Bachran D , González A , López B , Wachter R , Hasenfuss G , Delles C , Dominiczak AF *et al* (2018) Biomarker‐based phenotyping of myocardial fibrosis identifies patients with heart failure with preserved ejection fraction resistant to the beneficial effects of spironolactone: results from the Aldo‐DHF trial. Eur J Heart Fail 20: 1290–1299 29709099 10.1002/ejhf.1194

[emmm201910865-bib-0248] Reed MJ , Iruela‐Arispe L , O'Brien ER , Truong T , LaBell T , Bornstein P , Sage EH (1995) Expression of thrombospondins by endothelial cells. Injury is correlated with TSP‐1. Am J Pathol 147: 1068–1080 7573352 PMC1870996

[emmm201910865-bib-0249] Regan JA , Mauro AG , Carbone S , Marchetti C , Gill R , Mezzaroma E , Valle Raleigh J , Salloum FN , Van Tassell BW , Abbate A *et al* (2015) A mouse model of heart failure with preserved ejection fraction due to chronic infusion of a low subpressor dose of angiotensin II. Am J Physiol Heart Circ Physiol 309: H771–H778 26188021 10.1152/ajpheart.00282.2015 PMC4591411

[emmm201910865-bib-0250] Reutens AT , Jandeleit‐Dahm K , Thomas M , Bach LA , Colman PG , Davis TME , D'Emden M , Ekinci EI , Fulcher G , Hamblin PS *et al* (2019) A physician‐initiated double‐blind, randomised, placebo‐controlled, phase 2 study evaluating the efficacy and safety of inhibition of NADPH oxidase with the first‐in‐class Nox‐1/4 inhibitor, GKT137831, in adults with type 1 diabetes and persistently elevated urinary albumin excretion: protocol and statistical considerations. Contemp Clin Trials 90: 105892 31740428 10.1016/j.cct.2019.105892

[emmm201910865-bib-0251] Rice LM , Padilla CM , McLaughlin SR , Mathes A , Ziemek J , Goummih S , Nakerakanti S , York M , Farina G , Whitfield ML *et al* (2015) Fresolimumab treatment decreases biomarkers and improves clinical symptoms in systemic sclerosis patients. J Clin Invest 125: 2795–2807 26098215 10.1172/JCI77958 PMC4563675

[emmm201910865-bib-0252] Rickard AJ , Morgan J , Tesch G , Funder JW , Fuller PJ , Young MJ (2009) Deletion of mineralocorticoid receptors from macrophages protects against deoxycorticosterone/salt‐induced cardiac fibrosis and increased blood pressure. Hypertension 54: 537–543 19635989 10.1161/HYPERTENSIONAHA.109.131110

[emmm201910865-bib-0253] Rickard AJ , Morgan J , Bienvenu LA , Fletcher EK , Cranston GA , Shen JZ , Reichelt ME , Delbridge LM , Young MJ (2012) Cardiomyocyte mineralocorticoid receptors are essential for deoxycorticosterone/salt‐mediated inflammation and cardiac fibrosis. Hypertension 60: 1443–1450 23108646 10.1161/HYPERTENSIONAHA.112.203158

[emmm201910865-bib-0254] Rockey DC , Bell PD , Hill JA (2015) Fibrosis–a common pathway to organ injury and failure. N Engl J Med 372: 1138–1149 25785971 10.1056/NEJMra1300575

[emmm201910865-bib-0255] Rol N , de Raaf MA , Sun XQ , Kuiper VP , da SilvaGonçalvesBos D , Happé C , Kurakula K , Dickhoff C , Thuillet R , Tu L *et al* (2019) Nintedanib improves cardiac fibrosis but leaves pulmonary vascular remodelling unaltered in experimental pulmonary hypertension. Cardiovasc Res 115: 432–439 30032282 10.1093/cvr/cvy186

[emmm201910865-bib-0256] Rommel K‐P , von Roeder M , Latuscynski K , Oberueck C , Blazek S , Fengler K , Besler C , Sandri M , Lücke C , Gutberlet M *et al* (2016) Extracellular volume fraction for characterization of patients with heart failure and preserved ejection fraction. J Am Coll Cardiol 67: 1815–1825 27081022 10.1016/j.jacc.2016.02.018

[emmm201910865-bib-0257] Rosenbloom J , Macarak E , Piera‐Velazquez S , Jimenez SA (2017) Human fibrotic diseases: current challenges in fibrosis research. Methods Mol Biol 1627: 1–23 28836191 10.1007/978-1-4939-7113-8_1

[emmm201910865-bib-0258] Rudolph A , Abdel‐Aty H , Bohl S , Boyé P , Zagrosek A , Dietz R , Schulz‐Menger J (2009) Noninvasive detection of fibrosis applying contrast‐enhanced cardiac magnetic resonance in different forms of left ventricular hypertrophy relation to remodeling. J Am Coll Cardiol 53: 284–291 19147047 10.1016/j.jacc.2008.08.064

[emmm201910865-bib-0259] Santini V , Valcárcel D , Platzbecker U , Komrokji RS , Cleverly AL , Lahn MM , Janssen J , Zhao Y , Chiang A , Giagounidis A *et al* (2019) Phase II study of the ALK5 inhibitor galunisertib in very low‐, low‐, and intermediate‐risk myelodysplastic syndromes. Clin Cancer Res 25: 6976–6985 31481511 10.1158/1078-0432.CCR-19-1338

[emmm201910865-bib-0260] Schafer S , Viswanathan S , Widjaja AA , Lim W‐W , Moreno‐Moral A , DeLaughter DM , Ng B , Patone G , Chow K , Khin E *et al* (2017) IL‐11 is a crucial determinant of cardiovascular fibrosis. Nature 552: 110–115 29160304 10.1038/nature24676 PMC5807082

[emmm201910865-bib-0261] Schelbert EB , Hsu L‐Y , Anderson SA , Mohanty BD , Karim SM , Kellman P , Aletras AH , Arai AE (2010) Late gadolinium‐enhancement cardiac magnetic resonance identifies postinfarction myocardial fibrosis and the border zone at the near cellular level in *ex vivo* rat heart. Circ Cardiovasc Imaging 3: 743–752 20847191 10.1161/CIRCIMAGING.108.835793 PMC3398602

[emmm201910865-bib-0262] Schelbert EB , Fridman Y , Wong TC , Abu Daya H , Piehler KM , Kadakkal A , Miller CA , Ugander M , Maanja M , Kellman P *et al* (2017) Temporal relation between myocardial fibrosis and heart failure with preserved ejection fraction: association with baseline disease severity and subsequent outcome. JAMA Cardiol 2: 995–1006 28768311 10.1001/jamacardio.2017.2511 PMC5710176

[emmm201910865-bib-0263] Schellings MWM , Baumann M , van Leeuwen REW , Duisters RFJJ , Janssen SHP , Schroen B , Peutz‐Kootstra CJ , Heymans S , Pinto YM (2006) Imatinib attenuates end‐organ damage in hypertensive homozygous TGR(mRen2)27 rats. Hypertension 47: 467–474 16432052 10.1161/01.HYP.0000202487.68969.f7

[emmm201910865-bib-0264] Schnelle M , Sawyer I , Anilkumar N , Mohamed BA , Richards DA , Toischer K , Zhang M , Catibog N , Sawyer G , Mongue‐Din H *et al* (2019) NADPH oxidase‐4 promotes eccentric cardiac hypertrophy in response to volume overload. Cardiovasc Res 10.1093/cvr/cvz331 PMC7797217 31821410

[emmm201910865-bib-0265] Schorb W , Conrad KM , Singer HA , Dostal DE , Baker KM (1995) Angiotensin II is a potent stimulator of MAP‐kinase activity in neonatal rat cardiac fibroblasts. J Mol Cell Cardiol 27: 1151–1160 7473773 10.1016/0022-2828(95)90051-9

[emmm201910865-bib-0266] Schürmann C , Rezende F , Kruse C , Yasar Y , Löwe O , Fork C , van de Sluis B , Bremer R , Weissmann N , Shah AM *et al* (2015) The NADPH oxidase Nox4 has anti‐atherosclerotic functions. Eur Heart J 36: 3447–3456 26385958 10.1093/eurheartj/ehv460 PMC4751217

[emmm201910865-bib-0267] Schuster SJ , Svoboda J , Chong EA , Nasta SD , Mato AR , Anak Ö , Brogdon JL , Pruteanu‐Malinici I , Bhoj V , Landsburg D *et al* (2017) Chimeric antigen receptor T cells in refractory B‐cell lymphomas. N Engl J Med 377: 2545–2554 29226764 10.1056/NEJMoa1708566 PMC5788566

[emmm201910865-bib-0268] Sedgwick B , Riches K , Bageghni SA , O'Regan DJ , Porter KE , Turner NA (2014) Investigating inherent functional differences between human cardiac fibroblasts cultured from nondiabetic and Type 2 diabetic donors. Cardiovasc Pathol 23: 204–210 24746387 10.1016/j.carpath.2014.03.004

[emmm201910865-bib-0269] Seferovic PM , Ponikowski P , Anker SD , Bauersachs J , Chioncel O , Cleland JGF , de Boer RA , Drexel H , Ben Gal T , Hill L *et al* (2019) Clinical practice update on heart failure 2019: pharmacotherapy, procedures, devices and patient management. An expert consensus meeting report of the Heart Failure Association of the European Society of Cardiology. Eur J Heart Fail 21: 1169–1186 31129923 10.1002/ejhf.1531

[emmm201910865-bib-0270] Seniutkin O , Furuya S , Luo YS , Cichocki JA , Fukushima H , Kato Y , Sugimoto H , Matsumoto T , Uehara T , Rusyn I (2018) Effects of pirfenidone in acute and sub‐chronic liver fibrosis, and an initiation‐promotion cancer model in the mouse. Toxicol Appl Pharmacol 339: 1–9 29197520 10.1016/j.taap.2017.11.024

[emmm201910865-bib-0271] Sharma UC , Pokharel S , van Brakel TJ , van Berlo JH , Cleutjens JPM , Schroen B , André S , Crijns HJGM , Gabius H , Maessen J *et al* (2004) Galectin‐3 marks activated macrophages in failure‐prone hypertrophied hearts and contributes to cardiac dysfunction. Circulation 110: 3121–3128 15520318 10.1161/01.CIR.0000147181.65298.4D

[emmm201910865-bib-0272] Shen H , Wang J , Min J , Xi W , Gao Y , Yin L , Yu Y , Liu K , Xiao J , Zhang Y *et al* (2018) Activation of TGF‐β1/α‐SMA/Col I profibrotic pathway in fibroblasts by Galectin‐3 contributes to atrial fibrosis in experimental models and patients. Cell Physiol Biochem 47: 851–863 29807358 10.1159/000490077

[emmm201910865-bib-0273] Shi Q , Liu X , Bai Y , Cui C , Li J , Li Y , Hu S , Wei Y (2011) *In vitro* effects of pirfenidone on cardiac fibroblasts: proliferation, myofibroblast differentiation, migration and cytokine secretion. PLoS ONE 6: e28134 22132230 10.1371/journal.pone.0028134 PMC3223242

[emmm201910865-bib-0274] Shimizu M , Umeda K , Sugihara N , Yoshio H , Ino H , Takeda R , Okada Y , Nakanishi I (1993) Collagen remodelling in myocardia of patients with diabetes. J Clin Pathol 46: 32–36 7679418 10.1136/jcp.46.1.32 PMC501107

[emmm201910865-bib-0275] Shull MM , Ormsby I , Kier AB , Pawlowski S , Diebold RJ , Yin M , Allen R , Sidman C , Proetzel G , Calvin D (1992) Targeted disruption of the mouse transforming growth factor‐beta 1 gene results in multifocal inflammatory disease. Nature 359: 693–699 1436033 10.1038/359693a0 PMC3889166

[emmm201910865-bib-0276] Siddesha JM , Valente AJ , Sakamuri SSVP , Yoshida T , Gardner JD , Somanna N , Takahashi C , Noda M , Chandrasekar B (2013) Angiotensin II stimulates cardiac fibroblast migration via the differential regulation of matrixins and RECK. J Mol Cell Cardiol 65: 9–18 24095877 10.1016/j.yjmcc.2013.09.015 PMC3896127

[emmm201910865-bib-0277] Singh VP , Le B , Khode R , Baker KM , Kumar R (2008) Intracellular angiotensin II production in diabetic rats is correlated with cardiomyocyte apoptosis, oxidative stress, and cardiac fibrosis. Diabetes 57: 3297–3306 18829990 10.2337/db08-0805 PMC2584136

[emmm201910865-bib-0278] Smith JW II (2000) Tolerability and side‐effect profile of rhIL‐11. Oncology 14: 41–47 11033837

[emmm201910865-bib-0279] Smith CL , Baek ST , Sung CY , Tallquist MD (2011) Epicardial‐derived cell epithelial‐to‐mesenchymal transition and fate specification require PDGF receptor signaling. Circ Res 108: e15–e26 21512159 10.1161/CIRCRESAHA.110.235531 PMC3134964

[emmm201910865-bib-0280] Solomon SD , McMurray JJV , Anand IS , Ge J , Lam CSP , Maggioni AP , Martinez F , Packer M , Pfeffer MA , Pieske B *et al* (2019) Angiotensin‐neprilysin inhibition in heart failure with preserved ejection fraction. N Engl J Med 381: 1609–1620 31475794 10.1056/NEJMoa1908655

[emmm201910865-bib-0281] Somanna NK , Yariswamy M , Garagliano JM , Siebenlist U , Mummidi S , Valente AJ , Chandrasekar B (2015) Aldosterone‐induced cardiomyocyte growth, and fibroblast migration and proliferation are mediated by TRAF3IP2. Cell Signal 27: 1928–1938 26148936 10.1016/j.cellsig.2015.07.001

[emmm201910865-bib-0282] Somanna NK , Valente AJ , Krenz M , Fay WP , Delafontaine P , Chandrasekar B (2016) The Nox1/4 dual inhibitor GKT137831 or Nox4 knockdown inhibits angiotensin‐II‐induced adult mouse cardiac fibroblast proliferation and migration. AT1 physically associates with Nox4. J Cell Physiol 231: 1130–1141 26445208 10.1002/jcp.25210 PMC5237386

[emmm201910865-bib-0283] Song K , Nam Y‐J , Luo X , Qi X , Tan W , Huang GN , Acharya A , Smith CL , Tallquist MD , Neilson EG *et al* (2012) Heart repair by reprogramming non‐myocytes with cardiac transcription factors. Nature 485: 599–604 22660318 10.1038/nature11139 PMC3367390

[emmm201910865-bib-0284] Song X , Qian X , Shen M , Jiang R , Wagner MB , Ding G , Chen G , Shen B (2015) Protein kinase C promotes cardiac fibrosis and heart failure by modulating Galectin‐3 expression. Biochim Biophys Acta 1853: 513–521 25489662 10.1016/j.bbamcr.2014.12.001

[emmm201910865-bib-0285] Sorop O , Heinonen I , van Kranenburg M , van de Wouw J , de Beer VJ , Nguyen ITN , Octavia Y , van Duin RWB , Stam K , van Geuns R‐J *et al* (2018) Multiple common comorbidities produce left ventricular diastolic dysfunction associated with coronary microvascular dysfunction, oxidative stress, and myocardial stiffening. Cardiovasc Res 114: 954–964 29432575 10.1093/cvr/cvy038 PMC5967461

[emmm201910865-bib-0286] de Souza RR (2002) Aging of myocardial collagen. Biogerontology 3: 325–335 12510171 10.1023/a:1021312027486

[emmm201910865-bib-0287] Stauber AJ , Credille KM , Truex LL , Ehlhardt WJ , Young JK (2014) Nonclinical safety evaluation of a transforming growth factor β Receptor I kinase inhibitor in fischer 344 rats and beagle dogs. J Clin Toxicol 04: 3

[emmm201910865-bib-0288] Stockand JD , Meszaros JG (2003) Aldosterone stimulates proliferation of cardiac fibroblasts by activating Ki‐RasA and MAPK1/2 signaling. Am J Physiol Heart Circ Physiol 284: H176–H184 12388314 10.1152/ajpheart.00421.2002

[emmm201910865-bib-0289] Streng KW , Nauta JF , Hillege HL , Anker SD , Cleland JG , Dickstein K , Filippatos G , Lang CC , Metra M , Ng LL *et al* (2018) Non‐cardiac comorbidities in heart failure with reduced, mid‐range and preserved ejection fraction. Int J Cardiol 271: 132–139 30482453 10.1016/j.ijcard.2018.04.001

[emmm201910865-bib-0290] Suematsu Y , Miura S‐I , Goto M , Matsuo Y , Arimura T , Kuwano T , Imaizumi S , Iwata A , Yahiro E , Saku K (2016) LCZ696, an angiotensin receptor–neprilysin inhibitor, improves cardiac function with the attenuation of fibrosis in heart failure with reduced ejection fraction in streptozotocin‐induced diabetic mice. Eur J Heart Fail 18: 386–393 26749570 10.1002/ejhf.474

[emmm201910865-bib-0291] Sun Y , Ramires FJA , Weber KT (1997) Fibrosis of atria and great vessels in response to angiotensin II or aldosterone infusion. Cardiovasc Res 35: 138–147 9302358 10.1016/s0008-6363(97)00097-7

[emmm201910865-bib-0292] Susutlertpanya W , Wakuda H , Otani N , Kuramoto T , Li L , Kuranari M , Sekiguchi A , Kudo H , Uchida T , Imai H *et al* (2019) Histological evaluation of nintedanib in non‐alcoholic steatohepatitis mice. Life Sci 228: 251–257 31078545 10.1016/j.lfs.2019.05.014

[emmm201910865-bib-0293] Sverdlov AL , Elezaby A , Qin F , Behring JB , Luptak I , Calamaras TD , Siwik DA , Miller EJ , Liesa M , Shirihai OS *et al* (2016) Mitochondrial reactive oxygen species mediate cardiac structural, functional, and mitochondrial consequences of diet‐induced metabolic heart disease. J Am Heart Assoc 5: e002555 26755553 10.1161/JAHA.115.002555 PMC4859372

[emmm201910865-bib-0294] Swedberg K (1987) Effects of enalapril on mortality in severe congestive heart failure. N Engl J Med 316: 1429–1435 2883575 10.1056/NEJM198706043162301

[emmm201910865-bib-0295] Szardien S , Nef HM , Voss S , Troidl C , Liebetrau C , Hoffmann J , Rauch M , Mayer K , Kimmich K , Rolf A *et al* (2012) Regression of cardiac hypertrophy by granulocyte colony‐stimulating factor‐stimulated interleukin‐1β synthesis. Eur Heart J 33: 595–605 22106340 10.1093/eurheartj/ehr434

[emmm201910865-bib-0296] Tadevosyan A , Xiao J , Surinkaew S , Naud P , Merlen C , Harada M , Qi X , Chatenet D , Fournier A , Allen BG *et al* (2017) Intracellular angiotensin‐II interacts with nuclear angiotensin receptors in cardiac fibroblasts and regulates rna synthesis, cell proliferation, and collagen secretion. J Am Heart Assoc 6: e004965 28381466 10.1161/JAHA.116.004965 PMC5533010

[emmm201910865-bib-0297] Taipale J , Miyazono K , Heldin CH , Keski‐Oja J (1994) Latent transforming growth factor‐beta 1 associates to fibroblast extracellular matrix via latent TGF‐beta binding protein. J Cell Biol 124: 171–181 8294500 10.1083/jcb.124.1.171 PMC2119892

[emmm201910865-bib-0298] Takimoto E , Champion HC , Li M , Belardi D , Ren S , Rodriguez ER , Bedja D , Gabrielson KL , Wang Y , Kass DA (2005) Chronic inhibition of cyclic GMP phosphodiesterase 5A prevents and reverses cardiac hypertrophy. Nat Med 11: 214–222 15665834 10.1038/nm1175

[emmm201910865-bib-0299] Tallquist MD , Molkentin JD (2017) Redefining the identity of cardiac fibroblasts. Nat Rev Cardiol 14: 484–491 28436487 10.1038/nrcardio.2017.57 PMC6329009

[emmm201910865-bib-0300] Tamura N , Ogawa Y , Chusho H , Nakamura K , Nakao K , Suda M , Kasahara M , Hashimoto R , Katsuura G , Mukoyama M *et al* (2000) Cardiac fibrosis in mice lacking brain natriuretic peptide. Proc Natl Acad Sci USA 97: 4239–4244 10737768 10.1073/pnas.070371497 PMC18212

[emmm201910865-bib-0301] Tanaka K , Honda M , Takabatake T (2001) Redox regulation of MAPK pathways and cardiac hypertrophy in adult rat cardiac myocyte. J Am Coll Cardiol 37: 676–685 11216996 10.1016/s0735-1097(00)01123-2

[emmm201910865-bib-0302] Teekakirikul P , Eminaga S , Toka O , Alcalai R , Wang L , Wakimoto H , Nayor M , Konno T , Gorham JM , Wolf CM *et al* (2010) Cardiac fibrosis in mice with hypertrophic cardiomyopathy is mediated by non‐myocyte proliferation and requires Tgf‐β. J Clin Invest 120: 3520–3529 20811150 10.1172/JCI42028 PMC2947222

[emmm201910865-bib-0303] Tillmanns J , Hoffmann D , Habbaba Y , Schmitto JD , Sedding D , Fraccarollo D , Galuppo P , Bauersachs J (2015) Fibroblast activation protein alpha expression identifies activated fibroblasts after myocardial infarction. J Mol Cell Cardiol 87: 194–203 26319660 10.1016/j.yjmcc.2015.08.016

[emmm201910865-bib-0304] Treibel TA , López B , González A , Menacho K , Schofield RS , Ravassa S , Fontana M , White SK , DiSalvo C , Roberts N *et al* (2018a) Reappraising myocardial fibrosis in severe aortic stenosis: an invasive and non‐invasive study in 133 patients. Eur Heart J 39: 699–709 29020257 10.1093/eurheartj/ehx353 PMC5888951

[emmm201910865-bib-0305] Treibel TA , Kozor R , Schofield R , Benedetti G , Fontana M , Bhuva AN , Sheikh A , López B , González A , Manisty C *et al* (2018b) Reverse myocardial remodeling following valve replacement in patients with aortic stenosis. J Am Coll Cardiol 71: 860–871 29471937 10.1016/j.jacc.2017.12.035 PMC5821681

[emmm201910865-bib-0306] Tsai C‐F , Yang S‐F , Chu H‐J , Ueng K‐C (2013) Cross‐talk between mineralocorticoid receptor/angiotensin II type 1 receptor and mitogen‐activated protein kinase pathways underlies aldosterone‐induced atrial fibrotic responses in HL‐1 cardiomyocytes. Int J Cardiol 169: 17–28 24120080 10.1016/j.ijcard.2013.06.046

[emmm201910865-bib-0307] Turner NA , Mughal RS , Warburton P , O'Regan DJ , Ball SG , Porter KE (2007) Mechanism of TNFalpha‐induced IL‐1alpha, IL‐1beta and IL‐6 expression in human cardiac fibroblasts: effects of statins and thiazolidinediones. Cardiovasc Res 76: 81–90 17612514 10.1016/j.cardiores.2007.06.003

[emmm201910865-bib-0308] Umare V , Pradhan V , Nadkar M , Rajadhyaksha A , Patwardhan M , Ghosh KK , Nadkarni AH (2014) Effect of proinflammatory cytokines (IL‐6, TNF‐α, and IL‐1β) on clinical manifestations in Indian SLE patients. Mediators Inflamm 2014: 385297 25548434 10.1155/2014/385297 PMC4273527

[emmm201910865-bib-0309] Umbarkar P , Singh AP , Gupte M , Verma VK , Galindo CL , Guo Y , Zhang Q , McNamara JW , Force T , Lal H (2019) Cardiomyocyte SMAD4‐dependent TGF‐β signaling is essential to maintain adult heart homeostasis. JACC Basic Transl Sci 4: 41 30847418 10.1016/j.jacbts.2018.10.003 PMC6390466

[emmm201910865-bib-0310] Urata H , Kinoshita A , Misono KS , Bumpus FM , Husain A (1990) Identification of a highly specific chymase as the major angiotensin II‐forming enzyme in the human heart. J Biol Chem 265: 22348–22357 2266130

[emmm201910865-bib-0311] Vasan RS , Xanthakis V , Lyass A , Andersson C , Tsao C , Cheng S , Aragam J , Benjamin EJ , Larson MG (2018) Epidemiology of left ventricular systolic dysfunction and heart failure in the Framingham Study. JACC Cardiovasc Imaging 11: 1 28917679 10.1016/j.jcmg.2017.08.007 PMC5756128

[emmm201910865-bib-0312] Verheule S , Sato T , Everett T IV , Engle SK , Otten D , Rubart‐von der Lohe M , Nakajima HO , Nakajima H , Field LJ , Olgin JE (2004) Increased vulnerability to atrial fibrillation in transgenic mice with selective atrial fibrosis caused by overexpression of TGF‐beta1. Circ Res 94: 1458–1465 15117823 10.1161/01.RES.0000129579.59664.9d PMC2129102

[emmm201910865-bib-0313] Verma S , Garg A , Yan AT , Gupta AK , Al‐Omran M , Sabongui A , Teoh H , Mazer CD , Connelly KA (2016) Effect of empagliflozin on left ventricular mass and diastolic function in individuals with diabetes: an important clue to the EMPA‐REG outcome trial? Diabetes Care 39: e212 27679584 10.2337/dc16-1312

[emmm201910865-bib-0314] Villari B , Vassalli G , Monrad ES , Chiariello M , Turina M , Hess OM (1995) Normalization of diastolic dysfunction in aortic stenosis late after valve replacement. Circulation 91: 2353–2358 7729021 10.1161/01.cir.91.9.2353

[emmm201910865-bib-0315] Vincenti F , Fervenza FC , Campbell KN , Diaz M , Gesualdo L , Nelson P , Praga M , Radhakrishnan J , Sellin L , Singh A *et al* (2017) A phase 2, double‐blind, placebo‐controlled, randomized study of fresolimumab in patients with steroid‐resistant primary focal segmental glomerulosclerosis. Kidney Int Rep 2: 800–810 29270487 10.1016/j.ekir.2017.03.011 PMC5733825

[emmm201910865-bib-0316] Vitiello D , Harel F , Touyz RM , Sirois MG , Lavoie J , Myers J , Ducharme A , Racine N , O'Meara E , Gayda M *et al* (2014) Changes in cardiopulmonary reserve and peripheral arterial function concomitantly with subclinical inflammation and oxidative stress in patients with heart failure with preserved ejection fraction. Int J Vasc Med 2014: 917271 24719767 10.1155/2014/917271 PMC3955597

[emmm201910865-bib-0317] Voelker J , Berg PH , Sheetz M , Duffin K , Shen T , Moser B , Greene T , Blumenthal SS , Rychlik I , Yagil Y *et al* (2017) Anti‐TGF‐β1 antibody therapy in patients with diabetic nephropathy. J Am Soc Nephrol 28: 953–962 27647855 10.1681/ASN.2015111230 PMC5328150

[emmm201910865-bib-0318] Waddingham MT , Sonobe T , Tsuchimochi H , Edgley AJ , Sukumaran V , Chen YC , Hansra SS , Schwenke DO , Umetani K , Aoyama K *et al* (2019) Diastolic dysfunction is initiated by cardiomyocyte impairment ahead of endothelial dysfunction due to increased oxidative stress and inflammation in an experimental prediabetes model. J Mol Cell Cardiol 137: 119–131 31669609 10.1016/j.yjmcc.2019.10.005

[emmm201910865-bib-0319] Walther T , Schubert A , Falk V , Binner C , Kanev A , Bleiziffer S , Walther C , Doll N , Autschbach R , Mohr FW (2001) Regression of left ventricular hypertrophy after surgical therapy for aortic stenosis is associated with changes in extracellular matrix gene expression. Circulation 104: I54–I58 11568030 10.1161/hc37t1.094777

[emmm201910865-bib-0320] Wang J , Xu N , Feng X , Hou N , Zhang J , Cheng X , Chen Y , Zhang Y , Yang X (2005) Targeted disruption of Smad4 in cardiomyocytes results in cardiac hypertrophy and heart failure. Circ Res 97: 821–828 16151019 10.1161/01.RES.0000185833.42544.06

[emmm201910865-bib-0321] Wang M , Zhao D , Spinetti G , Zhang J , Jiang L‐Q , Pintus G , Monticone R , Lakatta EG (2006a) Matrix metalloproteinase 2 activation of transforming growth factor‐beta1 (TGF‐beta1) and TGF‐beta1‐type II receptor signaling within the aged arterial wall. Arterioscler Thromb Vasc Biol 26: 1503–1509 16690877 10.1161/01.ATV.0000225777.58488.f2

[emmm201910865-bib-0322] Wang W , Huang XR , Canlas E , Oka K , Truong LD , Deng C , Bhowmick NA , Ju W , Bottinger EP , Lan HY (2006b) Essential role of Smad3 in angiotensin II‐induced vascular fibrosis. Circ Res 98: 1032–1039 16556868 10.1161/01.RES.0000218782.52610.dc PMC1450325

[emmm201910865-bib-0323] Wang Y , Wu Y , Chen J , Zhao S , Li H (2013) Pirfenidone attenuates cardiac fibrosis in a mouse model of TAC‐Induced left ventricular remodeling by suppressing NLRP3 inflammasome formation. Cardiology 126: 1–11 23839341 10.1159/000351179

[emmm201910865-bib-0324] Weber KT (1989) Cardiac interstitium in health and disease: the fibrillar collagen network. J Am Coll Cardiol 13: 1637–1652 2656824 10.1016/0735-1097(89)90360-4

[emmm201910865-bib-0325] Wei CC , Hase N , Inoue Y , Bradley EW , Yahiro E , Li M , Naqvi N , Powell PC , Shi K , Takahashi Y *et al* (2010) Mast cell chymase limits the cardiac efficacy of Ang I‐converting enzyme inhibitor therapy in rodents. J Clin Invest 120: 1229–1239 20335663 10.1172/JCI39345 PMC2846039

[emmm201910865-bib-0326] Wells RG , Gilboa L , Sun Y , Liu X , Henis YI , Lodish HF (1999) Transforming growth factor‐beta induces formation of a dithiothreitol‐resistant type I/Type II receptor complex in live cells. J Biol Chem 274: 5716–5722 10026191 10.1074/jbc.274.9.5716

[emmm201910865-bib-0327] Westermann D , Kasner M , Steendijk P , Spillmann F , Riad A , Weitmann K , Hoffmann W , Poller W , Pauschinger M , Schultheiss H‐P *et al* (2008) Role of left ventricular stiffness in heart failure with normal ejection fraction. Circulation 117: 2051–2060 18413502 10.1161/CIRCULATIONAHA.107.716886

[emmm201910865-bib-0328] Westermann D , Lindner D , Kasner M , Zietsch C , Savvatis K , Escher F , von Schlippenbach J , Skurk C , Steendijk P , Riad A *et al* (2011) Cardiac inflammation contributes to changes in the extracellular matrix in patients with heart failure and normal ejection fraction. Circ Heart Fail 4: 44–52 21075869 10.1161/CIRCHEARTFAILURE.109.931451

[emmm201910865-bib-0329] Whittaker P , Kloner RA , Boughner DR , Pickering JG (1994) Quantitative assessment of myocardial collagen with picrosirius red staining and circularly polarized light. Basic Res Cardiol 89: 397–410 7535519 10.1007/BF00788278

[emmm201910865-bib-0330] Widjaja AA , Dong J , Adami E , Viswanathan S , Ng B , Singh BK , Lim WW , Zhou J , Pakkiri LS , Shekeran SG *et al* (2019a) Redefining interleukin 11 as a regeneration‐limiting hepatotoxin. bioRxiv 10.1101/830018 [PREPRINT] 34108253

[emmm201910865-bib-0331] Widjaja AA , Singh BK , Adami E , Viswanathan S , Dong J , D'Agostino GA , Ng B , Lim WW , Tan J , Paleja BS *et al* (2019b) Inhibiting interleukin 11 signaling reduces hepatocyte death and liver fibrosis, inflammation, and steatosis in mouse models of non‐alcoholic steatohepatitis. Gastroenterology 157: 777–792 31078624 10.1053/j.gastro.2019.05.002

[emmm201910865-bib-0332] Widyantoro B , Emoto N , Nakayama K , Anggrahini Dyah W , Adiarto S , Iwasa N , Yagi K , Miyagawa K , Rikitake Y , Suzuki T *et al* (2010) Endothelial cell‐derived endothelin‐1 promotes cardiac fibrosis in diabetic hearts through stimulation of endothelial‐to‐mesenchymal transition. Circulation 121: 2407–2418 20497976 10.1161/CIRCULATIONAHA.110.938217

[emmm201910865-bib-0333] Wipff P‐J , Rifkin DB , Meister J‐J , Hinz B (2007) Myofibroblast contraction activates latent TGF‐beta1 from the extracellular matrix. J Cell Biol 179: 1311–1323 18086923 10.1083/jcb.200704042 PMC2140013

[emmm201910865-bib-0334] Woods TC , Satou R , Miyata K , Katsurada A , Dugas CM , Klingenberg NC , Fonseca VA , Navar LG (2019) Canagliflozin prevents intrarenal angiotensinogen augmentation and mitigates kidney injury and hypertension in mouse model of type 2 diabetes mellitus. Am J Nephrol 49: 331–342 30921791 10.1159/000499597 PMC6475450

[emmm201910865-bib-0335] Wu C‐K , Su M‐Y , Lee J‐K , Chiang F‐T , Hwang J‐J , Lin J‐L , Chen J‐J , Liu F‐T , Tsai C‐T (2015) Galectin‐3 level and the severity of cardiac diastolic dysfunction using cellular and animal models and clinical indices. Sci Rep 5: 17007 26582585 10.1038/srep17007 PMC4652206

[emmm201910865-bib-0336] Wu J , Subbaiah KCV , Xie LH , Jiang F , Mickelsen D (2019) EPRS regulates proline‐rich pro‐fibrotic protein synthesis during cardiac fibrosis. bioRxiv 10.1101/777490 [PREPRINT] PMC7484271 32611237

[emmm201910865-bib-0337] Wynn TA (2008) Cellular and molecular mechanisms of fibrosis. J Pathol 214: 199–210 18161745 10.1002/path.2277 PMC2693329

[emmm201910865-bib-0338] Xiang Y , Shi W , Li Z , Yang Y , Wang SY , Xiang R , Feng P , Wen L , Huang W (2019) Efficacy and safety of spironolactone in the heart failure with mid‐range ejection fraction and heart failure with preserved ejection fraction: a meta‐analysis of randomized clinical trials. Medicine 98: e14967 30921200 10.1097/MD.0000000000014967 PMC6456096

[emmm201910865-bib-0339] Xu Y , Xiao H , Luo H , Chen Y , Zhang Y , Tao L , Jiang Y , Chen Y , Shen X (2017) Inhibitory effects of oxymatrine on TGF‐β1‐induced proliferation and abnormal differentiation in rat cardiac fibroblasts via the p38MAPK and ERK1/2 signaling pathways. Mol Med Rep 16: 5354–5362 28849213 10.3892/mmr.2017.7277 PMC5647068

[emmm201910865-bib-0340] Yamazaki T , Yamashita N , Izumi Y , Nakamura Y , Shiota M , Hanatani A , Shimada K , Muro T , Iwao H , Yoshiyama M (2012) The antifibrotic agent pirfenidone inhibits angiotensin II‐induced cardiac hypertrophy in mice. Hypertens Res 35: 34–40 21866107 10.1038/hr.2011.139

[emmm201910865-bib-0341] Yang JH , Obokata M , Reddy YNV , Redfield MM , Lerman A , Borlaug BA (2020) Endothelium‐dependent and independent coronary microvascular dysfunction in patients with heart failure with preserved ejection fraction. Eur J Heart Fail 22: 432–441 31840366 10.1002/ejhf.1671

[emmm201910865-bib-0342] Yao Y , Hu C , Song Q , Li Y , Da X , Yu Y , Li H , Clark IM , Chen Q , Wang QK (2020) ADAMTS16 activates latent TGF‐β, accentuating fibrosis and dysfunction of the pressure‐overloaded heart. Cardiovasc Res 116: 956–969 31297506 10.1093/cvr/cvz187 PMC7868664

[emmm201910865-bib-0343] Ye Y , Bajaj M , Yang HC , Perez‐Polo JR , Birnbaum Y (2017) SGLT‐2 inhibition with dapagliflozin reduces the activation of the Nlrp3/ASC inflammasome and attenuates the development of diabetic cardiomyopathy in mice with type 2 diabetes. further augmentation of the effects with saxagliptin, a DPP4 inhibitor. Cardiovasc Drugs Ther 31: 119–132 28447181 10.1007/s10557-017-6725-2

[emmm201910865-bib-0344] Ye J , Wang Z , Ye D , Wang Y , Wang M , Ji Q , Huang Y , Liu L , Shi Y , Shi L *et al* (2019) Increased interleukin‐11 levels are correlated with cardiac events in patients with chronic heart failure. Mediators Inflamm 2019: 8 10.1155/2019/1575410 PMC6341241 30728748

[emmm201910865-bib-0345] Yoo SY , Jeong S‐N , Kang J‐I , Lee S‐W (2018) Chimeric adeno‐associated virus‐mediated cardiovascular reprogramming for ischemic heart disease. ACS Omega 3: 5918–5925 30023931 10.1021/acsomega.8b00904 PMC6044635

[emmm201910865-bib-0346] Yoshida K , Matsuzaki K , Mori S , Tahashi Y , Yamagata H , Furukawa F , Seki T , Nishizawa M , Fujisawa J , Okazaki K (2005) Transforming growth factor‐beta and platelet‐derived growth factor signal via c‐Jun N‐terminal kinase‐dependent Smad2/3 phosphorylation in rat hepatic stellate cells after acute liver injury. Am J Pathol 166: 1029–1039 15793284 10.1016/s0002-9440(10)62324-3 PMC1602385

[emmm201910865-bib-0347] Yu Q , Stamenkovic I (2000) Cell surface‐localized matrix metalloproteinase‐9 proteolytically activates TGF‐beta and promotes tumor invasion and angiogenesis. Genes Dev 14: 163–176 10652271 PMC316345

[emmm201910865-bib-0348] Yu X , Deng L , Wang D , Li N , Chen X , Cheng X , Yuan J , Gao X , Liao M , Wang M *et al* (2012) Mechanism of TNF‐α autocrine effects in hypoxic cardiomyocytes: initiated by hypoxia inducible factor 1α, presented by exosomes. J Mol Cell Cardiol 53: 848–857 23085511 10.1016/j.yjmcc.2012.10.002

[emmm201910865-bib-0349] Yu L , Ruifrok Willem PT , Meissner M , Bos Eelke M , van Goor H , Sanjabi B , van der Harst P , Pitt B , Goldstein Irwin J , Koerts Jasper A *et al* (2013) Genetic and pharmacological inhibition of Galectin‐3 prevents cardiac remodeling by interfering with myocardial fibrogenesis. Circ Heart Fail 6: 107–117 23230309 10.1161/CIRCHEARTFAILURE.112.971168

[emmm201910865-bib-0350] Yusuf S , Pfeffer MA , Swedberg K , Granger CB , Held P , McMurray JJ , Michelson EL , Olofsson B , Ostergren J (2003) Effects of candesartan in patients with chronic heart failure and preserved left‐ventricular ejection fraction: the CHARM‐Preserved Trial. Lancet 362: 777–781 13678871 10.1016/S0140-6736(03)14285-7

[emmm201910865-bib-0351] Zeisberg EM , Tarnavski O , Zeisberg M , Dorfman AL , McMullen JR , Gustafsson E , Chandraker A , Yuan X , Pu WT , Roberts AB *et al* (2007) Endothelial‐to‐mesenchymal transition contributes to cardiac fibrosis. Nat Med 13: 952–961 17660828 10.1038/nm1613

[emmm201910865-bib-0352] Zhang Y , Feng XH , Derynck R (1998) Smad3 and Smad4 cooperate with c‐Jun/c‐Fos to mediate TGF‐beta‐induced transcription. Nature 394: 909–913 9732876 10.1038/29814

[emmm201910865-bib-0353] Zhang X , Stewart JA Jr , Kane ID , Massey EP , Cashatt DO , Carver WE (2007) Effects of elevated glucose levels on interactions of cardiac fibroblasts with the extracellular matrix. In Vitro Cell Dev Biol Anim 43: 297–305 17849168 10.1007/s11626-007-9052-2

[emmm201910865-bib-0354] Zhang M , Brewer AC , Schröder K , Santos CXC , Grieve DJ , Wang M , Anilkumar N , Yu B , Dong X , Walker SJ *et al* (2010) NADPH oxidase‐4 mediates protection against chronic load‐induced stress in mouse hearts by enhancing angiogenesis. Proc Natl Acad Sci USA 107: 18121–18126 20921387 10.1073/pnas.1009700107 PMC2964252

[emmm201910865-bib-0355] Zhao QD , Viswanadhapalli S , Williams P , Shi Q , Tan C , Yi X , Bhandari B , Abboud HE (2015) NADPH oxidase 4 induces cardiac fibrosis and hypertrophy through activating Akt/mTOR and NFκB signaling pathways. Circulation 131: 643–655 25589557 10.1161/CIRCULATIONAHA.114.011079 PMC4568756

[emmm201910865-bib-0356] Zhou L , Cryan EV , D'Andrea MR , Belkowski S , Conway BR , Demarest KT (2003) Human cardiomyocytes express high level of Na+/glucose cotransporter 1 (SGLT1). J Cell Biochem 90: 339–346 14505350 10.1002/jcb.10631

[emmm201910865-bib-0357] Zile MR , Baicu CF , Gaasch WH (2004) Diastolic heart failure–abnormalities in active relaxation and passive stiffness of the left ventricle. N Engl J Med 350: 1953–1959 15128895 10.1056/NEJMoa032566

[emmm201910865-bib-0358] Zile MR , Baicu CF , Ikonomidis JS , Stroud RE , Nietert PJ , Bradshaw AD , Slater R , Palmer BM , Van Buren P , Meyer M *et al* (2015) Myocardial stiffness in patients with heart failure and a preserved ejection fraction: contributions of collagen and titin. Circulation 131: 1247–1259 25637629 10.1161/CIRCULATIONAHA.114.013215 PMC4390480

[emmm201910865-bib-0359] Zile MR , O'Meara E , Claggett B , Prescott MF , Solomon SD , Swedberg K , Packer M , McMurray JJV , Shi V , Lefkowitz M *et al* (2019) Effects of sacubitril/valsartan on biomarkers of extracellular matrix regulation in patients with HFrEF. J Am Coll Cardiol 73: 795–806 30784673 10.1016/j.jacc.2018.11.042

[emmm201910865-bib-0501] Zinman B , Wanner C , Lachin JM , Fitchett D , Bluhmki E , Hantel S , Mattheus M , Devins T , Johansen OE , Woerle HJ *et al* (2015) Empagliflozin, Cardiovascular Outcomes, and Mortality in Type 2 Diabetes. N Engl J Med 373: 2117–2128 26378978 10.1056/NEJMoa1504720

